# Evaluating the Cost‐Effectiveness of Antenatal Screening for Major Structural Anomalies During the First Trimester of Pregnancy: A Decision Model

**DOI:** 10.1111/1471-0528.18053

**Published:** 2025-01-21

**Authors:** Helen E. Campbell, Jehan N. Karim, Aris T. Papageorghiou, Edward C. F. Wilson, Oliver Rivero‐Arias, the ACCEPTS Study, Aris T. Papageorghiou, Zarko Alfirevic, Trish Chudleigh, Hilary Goodman, Christos Ioannou, Heather Longworth, Jehan N. Karim, Kypros H. Nicolaides, Pranav Pandya, Gordon Smith, Basky Thilaganathan, Jim Thornton, Ed Juszczak, Louise Linsell, Ed Wilson, Lisa Hinton, Jane Fisher, Elizabeth Duff, Anne Rhodes, Gil Yaz

**Affiliations:** ^1^ National Perinatal Epidemiology Unit, Nuffield Department of Population Health University of Oxford Oxford UK; ^2^ Nuffield Department of Women's & Reproductive Health University of Oxford Oxford UK; ^3^ Oxford Maternal & Perinatal Health Institute, Green Templeton College University of Oxford Oxford UK; ^4^ Peninsula Technology Assessment Group, University of Exeter Medical School University of Exeter Exeter UK

**Keywords:** cost‐effectiveness, economic modelling, first trimester, foetal anomaly, screening, ultrasound, value of information

## Abstract

**Objective:**

To assess the cost‐effectiveness of modifying current antenatal screening by adding first trimester structural anomaly screening to standard of care second trimester anomaly screening.

**Design:**

Health economic decision model.

**Setting:**

National Health Service (NHS) in England and Wales.

**Population:**

Pregnant women attending for first trimester antenatal screening.

**Methods:**

The decision model estimated pregnancy outcomes (maternal and foetal) and 20‐year costs for current screening practice and for a policy adding a protocol screening for eight major structural anomalies to the current first trimester ultrasound scan. Event probabilities, costs, and outcomes for the model were informed by meta‐analyses, published literature, and expert opinion.

**Main Outcomes Measures:**

Expected numbers of pregnancy outcomes, healthcare costs, and maternal quality‐adjusted life years (QALYs). Estimation of the incremental cost‐effectiveness ratio (ICER), likelihood of cost‐effectiveness, and a value of information (VoI) analysis assessing if further research is needed before making a decision about screening.

**Results:**

First trimester anomaly screening increased mean per woman costs by £11 (95% CI £1–£29) and maternal QALYs by 0.002065 (95% CI 0.00056–0.00358). The ICER was £5270 per QALY and the probability of cost‐effectiveness at a willingness to pay value for a QALY of £20 000, exceeded 95%. VoI analysis showed further research would be unlikely to represent value for money. The protocol would likely lead to a reduction in infant healthcare costs and QALYs.

**Conclusions:**

A protocol to screen for eight major structural anomalies during the first trimester appears to represent value for money for the NHS. The opposing implications for mothers and infants, however, raise complex, challenging, and sensitive issues.

## 1 Introduction

In most high‐income settings, standard antenatal care includes two ultrasound screens, one offered at 11–14 weeks of gestation to confirm foetal viability, establish gestational age, and identify multiple pregnancies, and one at 18–20 weeks to identify major congenital anomalies [1, 2, 3]. England and Wales have followed this mode of antenatal screening, as recommended by the National Screening Committee.

This approach to screening has been challenged, with calls to screen for several major congenital anomalies during the first trimester. It has been argued that most foetal organ development is complete by 10 weeks, that earlier detection of these structural anomalies would complement first trimester screening for Trisomies 21, 18, and 13, and that improvements in ultrasound image quality, mean a number of major structural anomalies are now identifiable during the 11–14 week screen [2, 3, 4, 5, 6, 7, 8, 9, 10]. Despite an absence of formal guidance by the National Screening Committee, some three quarters of National Health Service (NHS) centres now perform such screening, resulting in inequalities in the provision of antenatal care across England and Wales [11].

Proponents of first trimester anomaly screening point to the reassurance that an early negative scan will provide to the majority of pregnant women [12]. For the minority receiving a positive result, an earlier diagnosis allows parents additional time for genetic testing, to receive input from multidisciplinary specialists, and ultimately to make the best decision for their family [13]. If this is to continue with the pregnancy, there will be more time to plan for the birth of the child. If the decision is to terminate the pregnancy, women will likely face lower likelihoods of procedure‐related complications, of needing to consent to foetocide, and of grief and post‐traumatic psychological distress, than if the termination had taken place during the second trimester [13, 14, 15, 16, 17, 18].

In contrast, those cautious about first trimester anomaly screening have voiced concerns that it could increase pregnancy terminations. Uncertainty also surrounds false positive screening findings and their implications [10]. Questions exist around early screening for anomalies that may resolve spontaneously with advancing gestational age. The small size of certain anatomical structures (e.g., the heart and the lungs), also means some parents will face anxiety if suspected anomalies cannot be confirmed until later gestations.

Consideration must also be given to whether first trimester anomaly screening represents value for money for the NHS. Population screening is costly, healthcare resources are scarce, and so it is pertinent to explore the impact on healthcare costs and outcomes; adoption will undoubtedly mean fewer resources to invest elsewhere in the NHS.

A health economic assessment was one objective of a research programme commissioned by the National Institute for Health and Care Research (NIHR) in England in response to the changing antenatal screening landscape. Recommendations from this work were for anomaly screening to be performed at 12–14 weeks gestation, primarily using trans‐abdominal ultrasound and at a minimum, targeting eight major anomalies: anencephaly, body stalk anomaly, major cardiac anomalies, encephalocele, exomphalos/omphalocele, holoprosencephaly, gastroschisis, and lower urinary tract obstruction (LUTO) [19].

To assess the health economic implications of implementing this anomaly screening protocol as part of the current first trimester (‘dating’) ultrasound screen, a decision analytic model was developed to predict the potential impact upon healthcare costs and outcomes. This article reports the results of this modelling exercise.

## 2 Methods

### 2.1 Model Scope

The cohort modelled was the general population of pregnant women attending for first trimester antenatal screening in England and Wales with a mean age of 30 years. We modelled only singleton pregnancies and screening with 2D ultrasound. The time horizon for the analysis was 20 years and the perspective was that of the NHS in England and Wales with costs expressed in 2019/2020 UK £. Outcomes were maternal quality‐adjusted life years (QALYs) with utility (quality of life) levels associated with women's health outcomes used to weight the durations for which the impact of those health outcomes persisted [20]. Utility is measured on a scale where 0 is equivalent to death and 1 to perfect health [21]. Costs and QALYs arising beyond the first 12 months were discounted at a rate of 3.5% [22]. Additional analyses considered the potential impact upon infant costs and QALYs.

Given the size and complexity of the model, detailed descriptions of its development and assumptions, and its input data, are provided in the Supporting Information, with abridged detail and appropriate signposting provided here.

### 2.2 Model Structure

**FIGURE 1:**
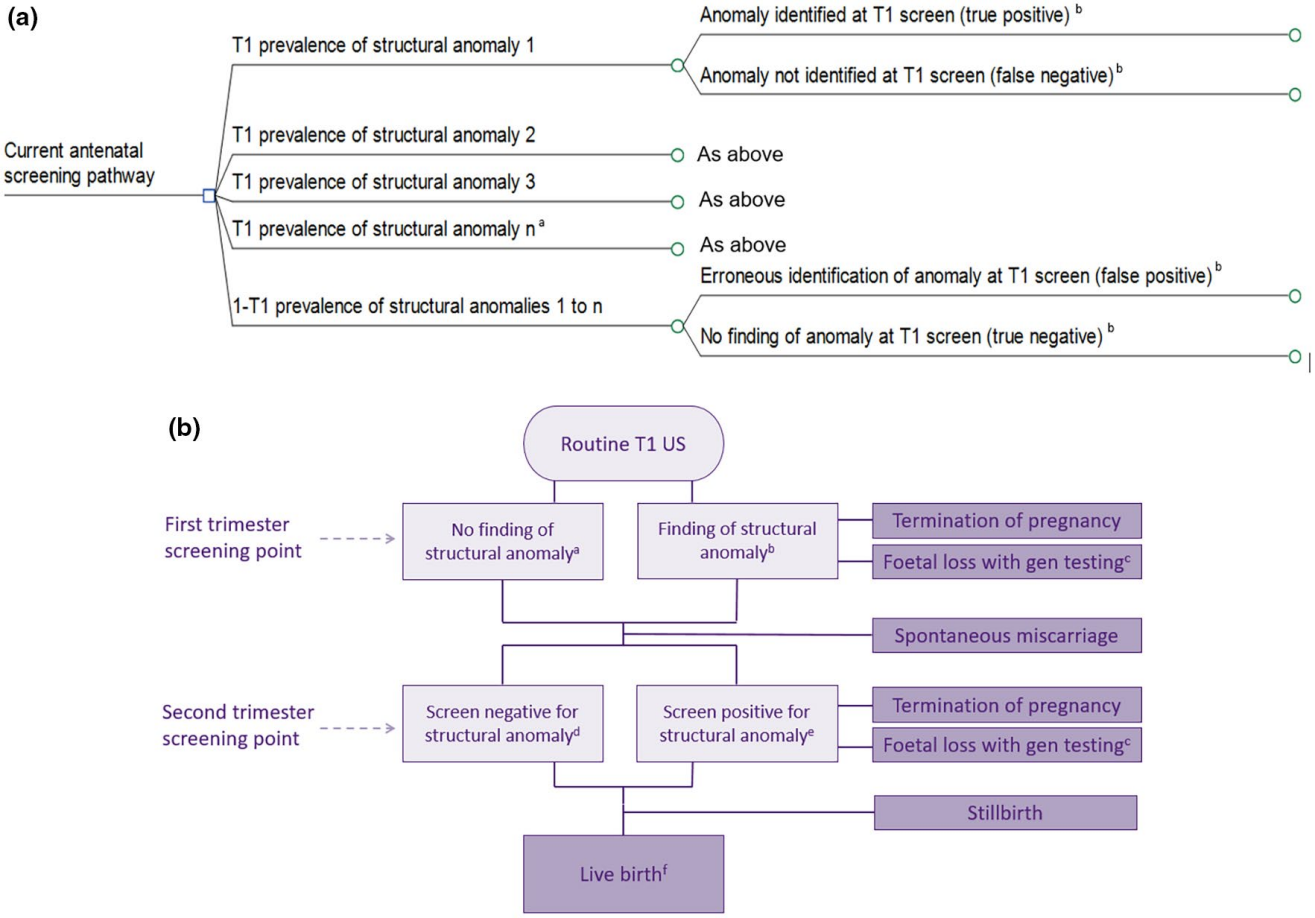
Overview of the structure of the decision tree model. Panel (a) Starting structure of decision tree. T1, first trimester; US, ultrasound. ^a^For brevity, branches for each individual anomaly are not shown. ^b^For ease, screening outcome terms (true positive, false positive etc.) are used despite no formal T1 anomaly screening currently taking place. Panel (b) Modelled screening and pregnancy outcomes along the antenatal pathway. ^a^Can be true negative or false negative (see panel [a] above). ^b^Can be true positive or false positive (see panel [a] above). ^c^Women screening positive for a structural anomaly with a strong genetic association enter a genetic testing sub‐tree (see Section 2 of supplementary file) and if consenting to testing face a risk of post‐procedural foetal loss. ^d^Can also be true negative or false negative finding. ^e^Can also be true positive or false positive finding. ^f^Live birth can be with or without an anomaly.

The model was conceived and built using TreeAge software (see Section 1 of the Data S1) [23]. A decision tree mapped out screening points and findings, and pregnancy outcomes along the current antenatal pathway (from 12 to 40 weeks' gestation). Figure 1, panel (a) shows the starting structure of the tree, beginning with first trimester prevalence branches for each of the eight anomalies and a single branch for women without an affected foetus (Section 1 of the Data S1 gives further detail on the anomalies modelled). Without formal first trimester anomaly screening, a level of case finding during the first trimester scan still exists, and so the prevalence branches are followed by screening outcome branches: true positive, false negative, false positive, and true negative findings. For each anomaly, individual sub‐trees (shown in Section 2 of the Data S1) model events following each of these four screening outcomes. Additionally, as five of the anomalies have a strong genetic association (holoprosencephaly, encephalocele, exomphalos/omphalocele, LUTO, and major cardiac anomaly), a further sub‐tree was developed to model the offer of invasive genetic testing and its associated risk for foetal loss, following a positive screen (Section 2 of the Data S1).

Figure 1, panel (b) illustrates the pregnancy outcomes included in the decision tree. They comprised: live birth with an anomaly, live birth without an anomaly, spontaneous miscarriage, spontaneous stillbirth, first trimester pregnancy termination, second trimester pregnancy termination, and first and second trimester foetal loss following genetic testing. Individual Markov models (shown in Section 3 of the Data S1) simulated the expected 20‐year healthcare costs and QALYs for women of each pregnancy outcome. Separate Markov models were required for women delivering live babies with each type of structural anomaly because a mother's level of well‐being is linked to that of her infant and the anomalies are heterogeneous with regard to infant mortality and morbidity.

### 2.3 Model Input Parameters

Where possible, model input parameters were entered as distributions rather than as single point estimates, to reflect the uncertainty inherent in sample data informing their values [24].

#### 2.3.1 Event Probabilities

Section 4 of the Data S1 presents the event probabilities used within the decision tree. Anomaly prevalences were informed by systematic reviews and meta‐analyses of first trimester anomaly screening studies [25, 26]. Current practice first trimester true positive screening probabilities were informed by UK registry data, supplemented by data from the systematic reviews in the ‘no protocol/current practice’ arm of studies [25, 26, 27]. First trimester false positive probabilities with current practice were estimated using literature identified during the systematic reviews (prioritising studies confirming false positive diagnoses via post‐mortem or postnatal examination) and by expert opinion [25, 26].

The systematic literature reviews also predominantly provided data for the screening outcome sub‐trees on the first trimester event probabilities for women carrying babies affected by each type of anomaly [25, 26, 28]. Second trimester event probabilities for women with affected babies were informed predominantly by UK foetal anomaly registry data [28]. General population data were used in the model for women carrying unaffected babies [29, 30].

Age and gender matched lifetable data informed annual maternal mortality risks across all Markov models [31]. For women giving birth to a baby with an anomaly, mortality risks were augmented (Section 5 of the Data S1) [32].

#### 2.3.2 Utilities

A full description of the modelling of utility within the decision tree is provided in Sections 6 and 7 of the Data S1. Women's underlying levels of utility were adjusted for the impact of the sequences of events they experience while moving through the model, for example, the immediate detrimental impacts of anomaly diagnoses and foetal loss, and the lasting impact of a false positive diagnosis.

Utility adjustments implemented in each of the Markov models are described in Section 8 of the Data S1. Underlying maternal utility was decremented over an appropriate duration, for the ongoing negative psychological impact of the pregnancy outcome experienced. These adjusted utilities weighted expected survival each year to facilitate the estimation of QALYs over 20 years.

#### 2.3.3 Costs

Sections 9 and 10 of the Data S1 describe the approach to costing within the model. Unit costs associated with events in the decision tree (e.g., ultrasound scans, diagnostic tests, spontaneous foetal losses, pregnancy terminations, and deliveries) were sourced predominantly from the National Schedule of NHS Costs [33]. Within the Markov models, costs included were for the annual treatment of ongoing negative psychological symptoms.

#### 2.3.4 Adding Protocolised First Trimester Anomaly Screening

The current practice arm of the model was replicated and branches permitting women to accept or decline a first trimester anomaly screening invitation were added prior to the prevalence branches. The acceptance rate (91%) was informed by responses from 1167 low‐risk pregnant women surveyed for the project [34]. Women declining the invitation followed the current practice pathway. Section 11 of the Data S1 details how the event probabilities, utilities, and costs were amended within the screening arm of the model. Briefly, and informed by the systematic reviews, women accepting a screening invitation, benefited from improved true positive detection rates for all eight anomalies, but also faced an increase in sonographer false positive rates for certain anomalies [25, 26]. All subsequent event probabilities remained as per the current practice arm of the model.

Two separate cost components associated with the protocol were included in the model; additional sonographer training to ensure proficiency in assessing parts of the foetal anatomy affected by the anomalies (£1.34 per scan), and 10 min of additional screening time required for the assessment (£10.80 per scan). Utilities in the anomaly screening arm were as per the current practice arm of the model with the exception of the addition of a transient (8‐week) first trimester utility increment of 0.01, included to reflect the likely beneficial albeit temporary effect of reassurance women receive following a negative anomaly screen [35, 36, 37].

### 2.4 Running the Model

The model was analysed probabilistically by running 10 000 simulations [24]. For each run, a new set of values from the model's input parameter distributions was sampled and cost‐effectiveness re‐calculated. For women in the general population carrying babies affected by the anomalies in the protocol, we predicted: expected numbers of live births, first trimester terminations, spontaneous miscarriages, second trimester terminations, stillbirths, and pregnancy losses following genetic testing. For the screening cohort as a whole, the expected mean per woman costs and maternal QALYs from the decision tree were added to those from the Markov model. When comparing between model arms, cost and QALY differences and 95% confidence intervals were calculated.

Cost‐effectiveness was expressed using the incremental cost‐effectiveness ratio (ICER) whereby the additional costs with first trimester anomaly screening were divided by the additional QALYs the policy generates. The ICER is interpreted as the additional cost of generating an additional QALY if implementing the protocol and was compared to a threshold value of £20 000 per QALY, which is considered to represent society's maximum willingness to pay for a QALY [38]. Uncertainty around the cost‐effectiveness results was explored using cost‐effectiveness acceptability curves (CEACs) [39]. Model results were also scaled up to a general population level assuming a cohort of 616 307 women per year (the total number of live births and stillbirths in England and Wales in 2020) attend for first trimester screening [40].

A deterministic sensitivity analysis on key study parameters was performed (see Section 12 of the Data S1). Analyses included increasing the additional screening time required to 20 min, and removing the temporary reassurance utility increment received by women following a negative screen.

Value of information (VoI) analysis explored the implications of the uncertainty surrounding the model's 175 input parameters by quantifying the expected consequences (the opportunity loss) of making the wrong decision (i.e., of implementing anomaly screening when it is in fact not cost‐effective) [41, 42]. This loss is equivalent to the expected gain if uncertainty across all model parameters could be removed and so is referred to as the Expected Value of Perfect Information (EVPI). Also estimated was the Expected Value of Partial Perfect Information (EVPPI) were the gains from eliminating uncertainty for groups of model parameters (e.g., screening performance probabilities, maternal utilities, screening costs, etc.) were explored. Such gains were compared to the expected costs of performing the additional research to reduce the uncertainty. If research costs exceeded the potential gains, the investment in additional research was not considered cost‐effective. Section 13 of the Data S1 provides details of the VoI analysis.

### 2.5 Infant Costs and QALYs

A further set of Markov models was developed to simulate expected 20‐year healthcare costs and QALYs of live born infants in each arm of the decision tree (see Section 14 of the Data S1). Separate models were constructed for infants born with each type of anomaly, with a genetic anomaly alone, and without an anomaly.

Anomaly specific mortality risks were used in the Markov models to estimate expected infant survival. General population norm infant utility levels were decremented for the expected quality of life impact of the anomaly affecting the child and the resulting utility levels were used to weight survival and generate infant QALYs. Treatment costs in the first days/years after birth were included in the models, along with longer‐term costs associated with the monitoring and further management of surviving children. The expected 20‐year costs and QALYs generated by these models were attached as secondary pay‐offs (using gamma distributions) to their corresponding live birth endpoints in the decision tree model. Zero infant healthcare costs and QALYs were assigned to those pregnancies ending prematurely without the live birth of a baby.

## 3 Results

Women with babies affected by the eight structural anomalies made up around 0.7% of all pregnant women attending for screening. Table 1 shows the pregnancy outcomes predicted for these women in each arm of the model at a general population level.

**TABLE 1:** Predicted numbers of pregnancy outcomes for women who have babies affected by any of the eight structural anomalies within the protocol, in England and Wales^a^.

Pregnancy outcome	Protocol *n* (SE)	Current practice *n* (SE)	Mean difference (95% CI)
Live birth	1878.60 (124.20)	2142.07 (89.54)	−263.47 (−503.77 to −48.06)
T1 termination	1686.82 (168.51)	1003.28 (112.55)	683.54 (374.65–1017.91)
Spontaneous miscarriage of foetus	245.32 (42.96)	263.40 (49.25)	−18.08 (−50.48 to 15.45)
T2 termination	392.42 (41.13)	778.85 (58.24)	−386.43 (−501.53 to −279.98)
Spontaneous late foetal loss/stillbirth	90.79 (9.26)	106.48 (9.98)	−15.69 (−25.09 to −7.91)
Foetal loss following genetic testing	5.12 (2.47)	5.00 (1.68)	0.12 (−1.71 to 3.28)

Abbreviations: CI, confidence interval; SE, standard error; T1, first trimester; T2, second trimester.

^a^ Numbers are based on a general population of 616 307 pregnant women attending for first trimester screening in England and Wales during a year.

The model estimates the protocol could lead to a 50% reduction in the number of second trimester terminations (from 779 to 392). By providing these women with information about their baby's condition at an earlier stage, the decision to terminate remains unchanged, but is now made during the first trimester. Almost three fifths of the predicted increase in first trimester terminations (from 1003 to 1686) with the protocol can be attributed to these earlier terminations. The remainder occur in women who may have previously continued with their pregnancy and had a live birth. Table 1 shows an estimated 263 fewer births of babies with an anomaly in the screening arm.

Among women with babies unaffected by an anomaly, there was no difference between the model arms in the numbers predicted to receive a foetal medicine false positive diagnosis following first trimester screening.

**FIGURE 2:**
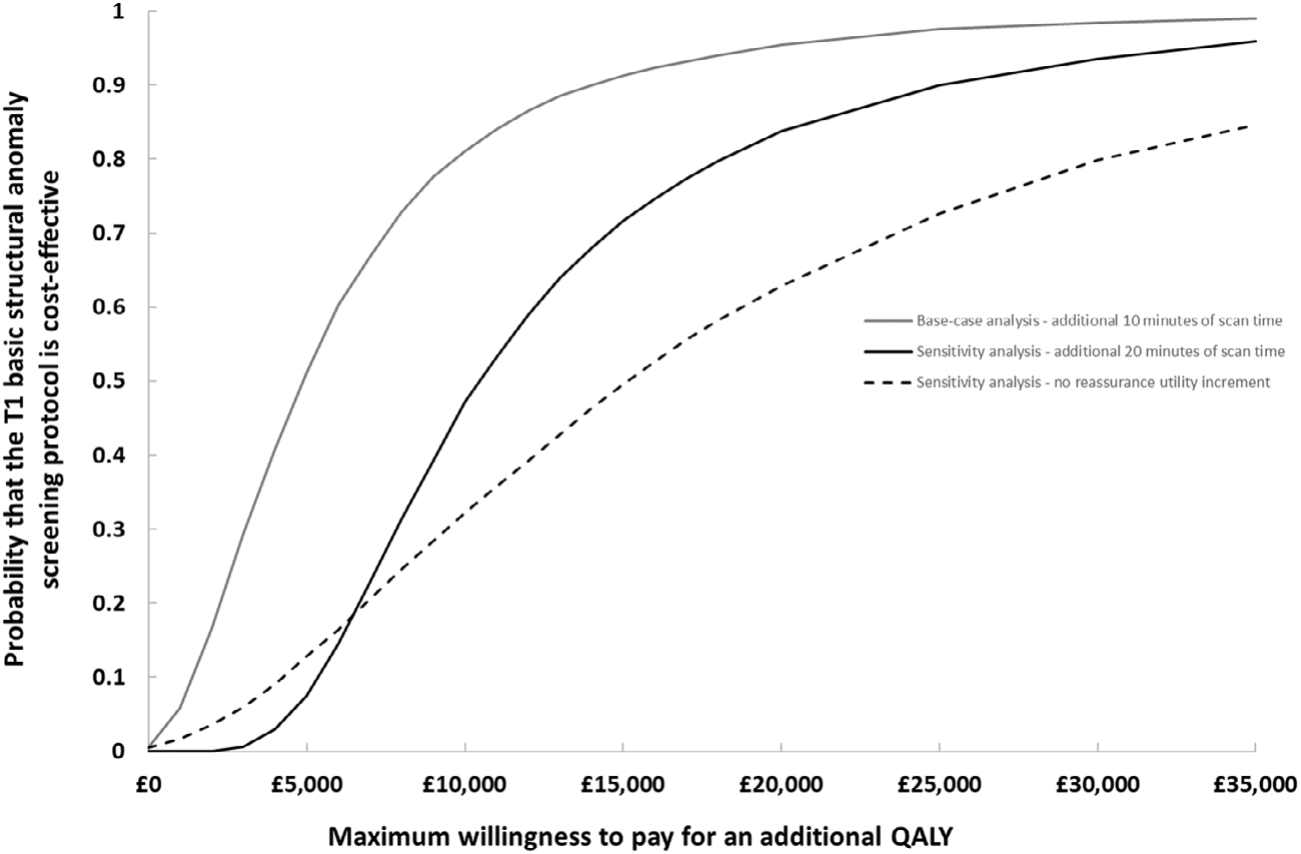
Cost‐effectiveness acceptability curves showing for the base‐case and key sensitivity analyses, the probability that the protocol is cost‐effective for different values of maximum willingness to pay for a QALY.

Table 2 shows estimated 20‐year mean total costs and maternal QALYs per pregnant woman, with these figures also scaled to a general population level. The protocol was associated with a mean cost increase of £11 (95% CI £1–£29) per woman and a mean QALY gain of 0.002065 (95% CI 0.00056–0.00358), resulting in an ICER of £5270 per QALY gained. Figure 2 plots the CEACs, showing the probability that the protocol is cost effective for different maximum willingness to pay values for a QALY, for both the base case and key sensitivity analyses. Base‐case results (solid grey line) suggested the probability of cost‐effectiveness at a willingness to pay value of £20 000 per QALY to be just over 95%.

**TABLE 2:** Estimated 20‐year mean costs and maternal QALYs per pregnant woman attending for antenatal screening, and total costs and maternal QALYs for all pregnant women attending for screening in England and Wales (*n* = 616 307).

	Protocol	Current practice	Mean difference (95% CI)
Per woman results			
Mean (SE) cost per woman	£4210 (£27)	£4199 (£26)	£11 (£1–£29)
Mean (SE) QALYs per woman	13.656 (0.083)	13.654 (0.083)	0.002065 (0.00056–0.00358)
Incremental cost‐effectiveness ratio			£5270 (£513‐£24 736)
Population level results			
Total (SE) Costs	£2 594 541 358 (£16 632 351)	£2 587 832 615 (£16 059 304)	£6 708 742 (£688 280–£17 598 285)
Total (SE) QALYs	8 416 545 (50935)	8 415 272 (50937)	1273 (348–2208)

Abbreviations: CI, confidence interval; SE, standard error.

Doubling the additional scanning time from 10 to 20 min increased the mean cost difference per woman to £22 (95% CI £10–£387), and the ICER from £5270 to £10 514. The probability of cost‐effectiveness at £20 000 per QALY was 83.7% (solid black line in Figure 2). Removing the 8‐week reassurance utility increment (0.01) assigned to screened women with a negative scan, reduced the mean QALY gain by two‐thirds to 0.000674 (95% CI 0.00020–0.00134). The ICER increased to £16 147 and the probability of the protocol being cost‐effective at £20 000 per QALY was 62.9% (dashed black line in Figure 2).

The EVPI for the number of women undergoing first trimester screening in England and Wales each year over a period of 20 years was estimated at £3 461 151 (£0.28 per woman). Although the expected cost of research to eliminate uncertainty across all 175 model parameters is unlikely to be < £3 461 151, EVPPI for the groups of parameters shown in Section 13 of the Data S1 was still conducted. Two groups contributed most to the per woman EVPPI: the screening performance probabilities (58 parameters) and the parameter for the additional cost associated with the anomaly screening. The former contributed 43% and for the screening population over 20 years the EVPPI was £87 836. The latter contributed 48% and the associated population EVPPI was £98 787.

Table 3 shows the expected 20‐year costs and QALYs for infants in the general population affected by the anomalies in the protocol. Significant changes to infant healthcare costs and QALYs were predicted with first trimester structural anomaly screening.

**TABLE 3:** Estimated 20‐year total costs and QALYs for infants affected by the eight structural anomalies in a population of 616 307 pregnant women attending for first trimester screening^a^.

	Protocol	Current practice	Mean difference (95% CI)
Total (SE) costs	£143 077 691 (£12 180 642)	£162 307 600 (£11 164 783)	−£19 229 909 (−£38 376 249 to −£2 440 806)
Total (SE) QALYs	18 442 (1259)	20 671 (960)	−2229 (−4522 to −198)

Abbreviations: CI, confidence interval; SE, standard error.

^a^ Figures calculated based on the assumption that all women have singleton pregnancies.

## 4 Discussion

### 4.1 Main Findings

In this study, we report on the development of a model simulating the short‐ and long‐term costs and consequences of modifying current antenatal screening pathways to include a protocol to screen for eight structural foetal anomalies during the first trimester ‘dating’ ultrasound screen.

The model predicts that with first trimester anomaly screening, around half of all parents currently choosing a pregnancy termination after a foetal anomaly diagnosis at the 20‐week anomaly scan, would make the same decision but now at an earlier gestational age. For a smaller proportion, it is predicted that first trimester anomaly screening would change their decision to continue with the pregnancy. With current practice, these women would not have had their baby's anomaly detected until the second trimester. They may not have wanted or may have felt unable to choose a termination at a more advanced gestational age when they would have been visibly pregnant, would have felt their baby move, and may have developed a greater sense of attachment.

The cost of implementing the protocol in practice is likely to be low because the necessary infrastructure is already in place via the NHS antenatal screening programme. The model assumes an additional 10 min of screening time is needed; however, this may be an overestimate; a survey of current practice we undertook across NHS sites found 75% were already routinely assessing parts of the foetal anomaly within the context and time constraints of the first trimester ‘dating’ scan [11]. Additional training costs to ensure sonographer competency with the protocol were also estimated; while for the purposes of the model, they were apportioned across all women screened over a period of 10 years (see Section 11 of the Data S1); in practice, the investment in staff training would be a larger upfront cost.

Two‐thirds of the QALY gain with first trimester anomaly screening arose from the ‘reassurance’ utility increment women receive following a negative first trimester anomaly scan. While the magnitude of this increment is small and transitory, it is received by around 90% of women in the protocol arm. While we could identify no studies reporting a ‘reassurance’ utility increment for use in the model, evidence from several published sources suggested a normal prenatal ultrasound screening result does provide reassuring qualities for women [35, 36, 37]. Furthermore, in a survey of low‐risk pregnant women conducted for this project, 86% in favour of a first trimester anomaly scan cited early reassurance as one of the main reasons that they would accept a screening invitation [34]. Guided by expert opinion we adopted a conservative approach by including a small utility increment (0.01) over a period of 8 weeks (up to the second trimester screening point).

Model results suggested a 95% probability that first trimester anomaly screening would be cost‐effective and that decision uncertainty was low. Through VoI analysis, population EVPPI estimates for groups of parameters associated with the additional costs of screening, and screening performance were shown to be greatest at £98 787 and £87 836, respectively. However, we considered it unlikely that additional research for either could be conducted for less than these figures. We concluded it would therefore not be a cost‐effective use of resources to conduct research to reduce this uncertainty further.

From the outset, we were acutely aware of the complexities and sensitivities involved in evaluating an antenatal screening programme that could increase maternal QALYs, yet potentially decrease infant QALYs. Previously, researchers working in the field have been criticised for either ignoring the implications for the infant, or for quantifying only the future cost savings arising as a result of the termination of a foetus with a disability [43, 44]. In taking the latter approach, Petrou noted that analysts ascribe the foetus or unborn child a future human status, and so should, by convention, also include some valuation of the health forgone [43].

To be both comprehensive and transparent, we estimated the potential impact of first trimester anomaly screening upon infant costs and QALYs. However, we could not, address the unresolved methodological issues around how to reconcile such opposing effects of antenatal screening [45, 46, 47]. We chose not to aggregate maternal and infant costs and QALYs to generate an overall estimate of cost‐effectiveness because such an approach would imply that the termination of a foetus with an anomaly, and the resulting loss of QALYs, is universally considered a devastating harm. For some in society this will be the case, however for others, including some ‘screen positive’ women, the feeling may be that a termination is ultimately in the best interests of the child and is not a negative outcome. Indeed, in a recent systematic review of health economic studies of antenatal and newborn screening programmes, Png et al. [47] noted that what constitutes a benefit or a harm will vary by stakeholder. We felt strongly that it was not our place to make such a value judgement and so reported the impact of screening for mothers and infants separately. Conclusions around cost‐effectiveness were based upon maternal costs and QALYs which are not subject to the same degree of uncertainty with regard to societal preferences.

### 4.2 Strengths and Limitations

This modelled analysis has a number of strengths. The structure of the model, by containing separate pathways for each of the anomalies in the protocol, recognises their heterogeneity and also permits a level of flexibility which can be used to explore the potential of alternative protocols including subsets of the anomalies modelled here. A further strength is that the first trimester anomaly screening parameters (true and false positive rates) that drive the changes to pregnancy outcomes, were estimated from two large meta‐analyses representing 63 studies (*n* = 328 262 foetuses [25]); and 52 studies (*n* = 526 322 foetuses [26]), respectively. Finally, the model captures the implications of screening, subsequent decisions, and pregnancy outcomes at both screening time points and into the future. It recognises the intrinsic link between mother and child and following a live birth, links the health outcomes of the mother to the prognosis of her child. We are unaware of any other antenatal screening model encompassing a similar level of complexity and detail.

As with any modelling study, limitations exist. Firstly, a number of simplifying assumptions relating to the structure of the model and estimation of some parameters were necessary, and we have documented and justified these (see Sections 1 and 2 of the Data S1). Assumptions made were often conservative towards screening. For example, costing the need for an extra 10 min for anomaly screening is possibly an overestimate; three quarters of NHS sites already routinely assess parts of the foetal anomaly during the current first trimester ‘dating’ scan. Similarly, when incorporating the reassuring effects of a negative anomaly scan, we opted to use only a small utility increment maintained for just 8 weeks. The implications of these assumptions, which affect parameters assigned to all screened women in the model, are that the cost‐effectiveness results reported may be conservative in nature. An absence of utility data meant it was also necessary to make assumptions about the impact of some screening and pregnancy outcomes upon maternal wellbeing. We attempted to be considered in our approach to this, for example, utilising existing literature on the proportions of women experiencing psychological symptoms (e.g., anxiety/depression) following each pregnancy outcome and then by applying published utility decrements for these conditions to the relevant proportions of women. Overall and despite the assumptions required by the model, VoI analysis demonstrated that decision uncertainty around the model's results was low and that further research to reduce uncertainty would probably not be a cost‐effective use of scarce resources.

Second, the NHS perspective adopted for the analysis may be considered narrow. The implications of first trimester screening for structural anomalies will be far reaching, touching partners, wider family members, and employers. Third, in some instances, we were forced to utilise data from countries comparable to the United Kingdom in terms of their GDP and healthcare provision. On occasions when no data were available, we sought expert advice from obstetricians working within the NHS. Fourth, the time horizon for the analysis was restricted to 20 years on account of the limited availability of data pertaining to the long‐term implications (for mothers and infants) of some of the rarer anomalies included in the model. Had it been possible to model over a longer duration, it is unlikely that the overall cost‐effectiveness results would have been altered substantially. Findings were driven mainly by the cost of additional screening time and by the reassurance provided by a negative scan; both of these parameters are assigned only once, to around 90% of women in the screening arm of the model, in the first weeks of the analysis. Further, and with the combined anomaly prevalence at 0.7%, the implications of first trimester anomaly screening will continue beyond 20 years for only a small proportion of all screened pregnant women. Fifth, and given the rarity of conditions being modelled, it was challenging to estimate infant costs and QALYs as few studies report data of the management, surveillance and outcomes for sizeable cohorts of children born with these anomalies. As a result, infant healthcare costs (and their associated uncertainty) in particular are likely to be underestimated. Finally, the generalisability of our cost‐effectiveness results must be considered. The model's focus is upon singleton pregnancies only, yet between 1% and 2% of women present with multiple pregnancies each year. It is likely that cost‐effectiveness results for this group of women could differ from those reported here; anomaly prevalence rates are known to be higher in multiple pregnancies and it is possible that the psychological impact of the various pregnancy outcomes modelled may be different for women carrying more than one foetus [48, 49]. It is hypothesised that the results presented here will be generalisable to settings where the anomaly screening programme is delivered using similar procedures as the one currently followed by the NHS. If this is not the case, however, a model adaptation will be required to confirm the likely cost‐effectiveness of implementing the protocol in those settings.

## 5 Conclusions

Through the development of a substantial health economic model, this study has shown that the addition of a protocol to screen for eight major structural foetal anomalies during the current first trimester ultrasound scan is likely to represent a cost‐effective use of NHS resources. Decision uncertainty appears low and VoI analyses suggested further research to reduce uncertainty around model parameters would not offer value for money. The work has also recognised the sensitivities and complexities involved in evaluating an antenatal screening programme for foetal anomalies and in acknowledging the opposing effects for mother and infant sought to be comprehensive and transparent in reporting the potential impact for both.

## Author Contributions

H.E.C. built and populated the health economic model, generated the results, drafted and revised the manuscript. J.N.K. provided critical clinical input into the design of the health economic model, provided clinical input data, and helped draft and revise the manuscript. A.T.P. was the principal investigator for the NIHR project, provided clinical expertise around the design and population of the model, and read and helped revise the manuscript. E.C.F.W. provided guidance on the health economic modelling and the design of the VoI analysis, and read and helped revise the manuscript. O.R.‐A. was the lead economist, supervised the health economic modelling, conducted the VoI analysis, and helped draft and revise the manuscript.

## Ethics Statement

The authors have nothing to report.

## Conflicts of Interest

The authors declare no conflicts of interest.

## Supporting information


Data S1.

## Data Availability

Previously published data used to generate the model results are detailed in the Supporting Information.

## References

[1] P. Ward and P. Soothill , “Fetal Anomaly Ultrasound Scanning: The Development of a National Programme for England,” Obstetrician & Gynaecologist 13, no. 4 (2011): 211–217.

[2] National Collaborating Centre for Women's and Children's Health , “National Institute for Health and Clinical Excellence: Guidance. Antenatal Care: Routine Care for the Healthy Pregnant Woman,” (2008).

[3] L. J. Salomon , Z. Alfirevic , C. M. Bilardo , et al., “ISUOG Practice Guidelines: Performance of First‐Trimester Fetal Ultrasound Scan,” Ultrasound in Obstetrics & Gynecology: The Official Journal of the International Society of Ultrasound in Obstetrics and Gynecology 41, no. 1 (2013): 102–113. 23280739 10.1002/uog.12342

[4] A. Abuhamad and R. Chaoui , First Trimester Ultrasound Diagnosis of Fetal Abnormalities, 1st ed., (Philadelphia, Pennsylvania, Philadelphia: Lippincott Williams & Wilkins, 2017).

[5] J. C. Donnelly and F. D. Malone , “Early Fetal Anatomical Sonography,” Best Practice & Research. Clinical Obstetrics & Gynaecology 26, no. 5 (2012): 561–573. 22776410 10.1016/j.bpobgyn.2012.06.002

[6] A. P. Souka , A. Pilalis , Y. Kavalakis , Y. Kosmas , P. Antsaklis , and A. Antsaklis , “Assessment of Fetal Anatomy at the 11–14‐Week Ultrasound Examination,” Ultrasound in Obstetrics & Gynecology: The Official Journal of the International Society of Ultrasound in Obstetrics and Gynecology 24, no. 7 (2004): 730–734. 15586371 10.1002/uog.1775

[7] C. Luchi , M. Schifano , C. Sacchini , et al., “Detailed Fetal Anatomy Assessment in the First Trimester at 11, 12 and 13 Weeks of Gestation,” Journal of Maternal‐Fetal and Neonatal Medicine 25, no. 6 (2012): 675–678. 21699438 10.3109/14767058.2011.587058

[8] A. Syngelaki , A. Hammami , S. Bower , V. Zidere , R. Akolekar , and K. H. Nicolaides , “Diagnosis of Fetal Non‐Chromosomal Abnormalities on Routine Ultrasound Examination at 11–13 Weeks' Gestation,” Ultrasound in Obstetrics & Gynecology: The Official Journal of the International Society of Ultrasound in Obstetrics and Gynecology 54, no. 4 (2019): 468–476. 31408229 10.1002/uog.20844

[9] Y. Liao , H. Wen , S. Ouyang , et al., “Routine First‐Trimester Ultrasound Screening Using a Standardized Anatomical Protocol,” American Journal of Obstetrics and Gynecology 224, no. 4 (2021): 396.e1–396.e15. 10.1016/j.ajog.2020.10.037 33127430

[10] J. N. Karim , N. W. Roberts , L. J. Salomon , and A. T. Papageorghiou , “Systematic Review of First‐Trimester Ultrasound Screening for Detection of Fetal Structural Anomalies and Factors That Affect Screening Performance,” Ultrasound in Obstetrics & Gynecology: The Official Journal of the International Society of Ultrasound in Obstetrics and Gynecology 50, no. 4 (2017): 429–441. 27546497 10.1002/uog.17246

[11] J. Karim , P. Pandya , A. McHugh , and A. T. Papageorghiou , “OC23.06: Significant Variation in Practice for First Trimester Anatomy Assessment: Results From a Nationwide Survey,” Ultrasound in Obstetrics & Gynecology 54 (2019): 60–61.

[12] F. Bardi , M. Bakker , M. J. A. Kenkhuis , et al., “Psychological Outcomes, Knowledge and Preferences of Pregnant Women on First‐Trimester Screening for Fetal Structural Abnormalities: A Prospective Cohort Study,” PLoS One 16, no. 1 (2021): e0245938. 33503072 10.1371/journal.pone.0245938 PMC7840026

[13] Royal College of Obstetricians and Gynaecologists , Termination of Pregnancy for Fetal Abnormality in England, Scotland and Wales (London, UK: Royal College of Obstetricians and Gynaecologists, 2010).

[14] Royal College of Obstetricians and Gynaecologists , Best Practice in Abortion Care. Making Abortion Safe—RCOG's Global Initiative to Advocate for Women's Health (London, UK: Royal College of Obstetricians and Gynaecologists, 2022).

[15] Department of Health and Social Care , “Abortion Statistics, England and Wales,” (2010), https://www.gov.uk/government/statistics/abortion‐statistics‐england‐and‐wales‐2010.

[16] B. Stewart , S. C. Kane , and J. Unterscheider , “Medical Termination of Pregnancy for Fetal Anomaly at or Beyond 20 Weeks' Gestation‐What Are the Maternal Risks?,” Prenatal Diagnosis 42, no. 12 (2022): 1562–1570. 36156270 10.1002/pd.6241

[17] M. J. Korenromp , G. C. M. L. Christiaens , J. van den Bout , et al., “Long‐Term Psychological Consequences of Pregnancy Termination for Fetal Abnormality: A Cross‐Sectional Study,” Prenatal Diagnosis 25, no. 3 (2005): 253–260. 15791682 10.1002/pd.1127

[18] V. Davies , J. Gledhill , A. McFadyen , B. Whitlow , and D. Economides , “Psychological Outcome in Women Undergoing Termination of Pregnancy for Ultrasound‐Detected Fetal Anomaly in the First and Second Trimesters: A Pilot Study,” Ultrasound in Obstetrics & Gynecology: The Official Journal of the International Society of Ultrasound in Obstetrics and Gynecology 25, no. 4 (2005): 389–392. 15791695 10.1002/uog.1854

[19] J. Karim , S. Gordijn , and A. T. Papageorghiou , “OC18.08: Developing a First Trimester Anomaly Screening Protocol for the UK: A Consensus Procedure,” Ultrasound in Obstetrics & Gynecology 58, no. S1 (2021): 54–55.

[20] Y. B. Vergel and M. Sculpher , “Quality‐Adjusted Life Years,” Practical Neurology 8, no. 3 (2008): 175–182. 18502950 10.1136/pn.2007.140186

[21] S. J. Whitehead and S. Ali , “Health Outcomes in Economic Evaluation: The QALY and Utilities,” British Medical Bulletin 96, no. 1 (2010): 5–21. 21037243 10.1093/bmb/ldq033

[22] HM Treasury , The Green Book. Central Government Guidance on Appraisal and Evaluation (London, UK: HM Treasury, 2023).

[23] TreeAge Software LLC , TreeAge pro Healthcare (Williamstown, Massachusetts: TreeAge Software LLC, 2022).

[24] A. Briggs , M. Sculpher , and K. Claxton , Decision Modelling for Health Economic Evaluation (Oxford, UK: Oxford University Press, 2006).

[25] J. N. Karim , E. Bradburn , N. Roberts , and A. T. Papageorghiou , “First‐Trimester Ultrasound Detection of Fetal Heart Anomalies: Systematic Review and Meta‐Analysis,” Ultrasound in Obstetrics & Gynecology: The Official Journal of the International Society of Ultrasound in Obstetrics and Gynecology 59, no. 1 (2022): 11–25. 34369613 10.1002/uog.23740 PMC9305869

[26] J. Karim , D. Di Mascio , N. Roberts , and A. T. Papageorghiou , “Detection of Non‐Cardiac Fetal Abnormalities by Ultrasound at 11–14 Weeks: Systematic Review and Meta‐Analysis,” Ultrasound in Obstetrics & Gynecology: The Official Journal of the International Society of Ultrasound in Obstetrics and Gynecology 64 (2024): 15–27, 10.1002/uog.27649. 38547384

[27] European Network of Population Based Registries for the Epidemiological Surveillance of Congenital Anomalies EUROCAT , “Prenatal Detection Rates Charts and Tables 2015–2019,” (2022), https://eu‐rd‐platform.jrc.ec.europa.eu/eurocat/eurocat‐data/prenatal‐screening‐and‐diagnosis_en.

[28] European Network of Population Based Registries for the Epidemiological Surveillance of Congenital Anomalies EUROCAT , “Prevalence Charts and Tables for Congenital Fetal Anomalies (UK) 2011–2018,” (2021), https://eu‐rd‐platform.jrc.ec.europa.eu/eurocat/eurocat‐data/prevalence_en.

[29] E. S. Draper, I. D. Gallimore , L. K. Smith , J. J. Kurinczuk , et al., MBRRACE‐UK Perinatal Mortality Surveillance Report, UK Perinatal Deaths for Births From January to December 2017 (Leicester, UK: Infant Mortality and Morbidity Studies, 2019).

[30] L. J. Salomon , A. Sotiriadis , C. B. Wulff , A. Odibo , and R. Akolekar , “Risk of Miscarriage Following Amniocentesis or Chorionic Villus Sampling: Systematic Review of Literature and Updated Meta‐Analysis,” Ultrasound in Obstetrics & Gynecology: The Official Journal of the International Society of Ultrasound in Obstetrics and Gynecology 54, no. 4 (2019): 442–451. 31124209 10.1002/uog.20353

[31] Office for National Statistics , “National Life Tables: England and Wales 2017–2019,” (2020), https://www.ons.gov.uk/peoplepopulationandcommunity/birthsdeathsandmarriages/lifeexpectancies/datasets/nationallifetablesenglandandwalesreferencetables/current.

[32] A. E. Fuller , E. Horváth‐Puhó , J. G. Ray , V. Ehrenstein , H. T. Sørensen , and E. Cohen , “Mortality Among Parents of Children With Major Congenital Anomalies,” Pediatrics 147, no. 5 (2021): e2020028571. 33811179 10.1542/peds.2020-028571

[33] NHS England , “National Schedule of NHS Costs 2019/20,” (2021), https://www.england.nhs.uk/national‐cost‐collection/#nccdata2.

[34] J. N. Karim , R. Craik , L. M. Davidson , et al., “Acceptability of the First Trimester Anomaly Scan Amongst Parents in the UK,” BJOG: International Journal of Obstetetrics and Gynaecology 128 (2021): 271–281.

[35] G. M. Thomas , J. Roberts , and F. E. Griffiths , “Ultrasound as a Technology of Reassurance? How Pregnant Women and Health Care Professionals Articulate Ultrasound Reassurance and Its Limitations,” Sociology of Health & Illness 39, no. 6 (2017): 893–907. 28326555 10.1111/1467-9566.12554

[36] J. Garcia , L. Bricker , J. Henderson , et al., “Women's Views of Pregnancy Ultrasound: A Systematic Review,” Birth 29, no. 4 (2002): 225–250. 12431263 10.1046/j.1523-536x.2002.00198.x

[37] S. Clement , J. Wilson , and J. Sikorski , “Women's Experiences of Antenatal Ultrasound Scans,” in Psychological Perspectives on Pregnancy and Childbirth, ed. S. Clement (Edinburgh: Churchill Livingstone, 1998), 7–24.

[38] National Institute for Health and Care Excellence , “NICE Health Technology Evaluations: The Manual. Process and Methods,” (2022), https://www.nice.org.uk/process/pmg36/chapter/committee‐recommendations2022.

[39] E. Fenwick and S. Byford , “A Guide to Cost‐Effectiveness Acceptability Curves,” British Journal of Psychiatry 187, no. 2 (2005): 106–108. 10.1192/bjp.187.2.106 16055820

[40] Office for National Statistics , “Births in England and Wales: 2020 Live Births, Stillbirths and the Intensity of Childbearing, Measured by the Total Fertility Rate,” (2020), https://www.ons.gov.uk/peoplepopulationandcommunity/birthsdeathsandmarriages/livebirths/bulletins/birthsummarytablesenglandandwales/2020.

[41] E. C. F. Wilson , “A Practical Guide to Value of Information Analysis,” PharmacoEconomics 33, no. 2 (2015): 105–121. 25336432 10.1007/s40273-014-0219-x

[42] E. Fenwick , L. Steuten , S. Knies , et al., “Value of Information Analysis for Research Decisions‐an Introduction: Report 1 of the ISPOR Value of Information Analysis Emerging Good Practices Task Force,” Value in Health: The Journal of the International Society for Pharmacoeconomics and Outcomes Research 23, no. 2 (2020): 139–150. 32113617 10.1016/j.jval.2020.01.001

[43] S. Petrou , “Methodological Limitations of Economic Evaluations of Antenatal Screening,” Health Economics 10, no. 8 (2001): 775–778. 11747056 10.1002/hec.636

[44] S. Petrou , J. Henderson , T. Roberts , and M. A. Martin , “Recent Economic Evaluations of Antenatal Screening: A Systematic Review and Critique,” Journal of Medical Screening 7, no. 2 (2000): 59–73. 11002445 10.1136/jms.7.2.59

[45] S. E. Little , V. Janakiraman , A. Kaimal , T. Musci , J. Ecker , and A. B. Caughey , “The Cost‐Effectiveness of Prenatal Screening for Spinal Muscular Atrophy,” American Journal of Obstetrics and Gynecology 202, no. 3 (2010): 253.e1–253.e7. 10.1016/j.ajog.2010.01.032 20207244

[46] J. Karnon , E. Goyder , P. Tappenden , et al., “A Review and Critique of Modelling in Prioritising and Designing Screening Programmes,” Health Technology Assessment 11, no. 52 (2007): 11520. 10.3310/hta11520 18031651

[47] M. E. Png , M. Yang , N. Roberts , S. Taylor‐Phillips , O. Rivero‐Arias , and S. Petrou , “Methods for Evaluating the Benefits and Harms of Antenatal and Newborn Screening Programmes Adopted by Health Economic Assessments: Protocol for a Systematic Review,” BMJ Open 11, no. 8 (2021): e048031. 10.1136/bmjopen-2020-048031 PMC8386210 34429311

[48] J. E. Shin , H. S. Ko , J. Y. Bae , et al., “Congenital Anomalies in Multiple Pregnancy: A Literature Review,” Obstetrical & Gynecological Survey 79, no. 3 (2024): 167–175. 38482746 10.1097/OGX.0000000000001251

[49] F. D'Antonio , A. Familiari , B. Thilaganathan , et al., “Sensitivity of First‐Trimester Ultrasound in the Detection of Congenital Anomalies in Twin Pregnancies: Population Study and Systematic Review,” Acta Obstetricia et Gynecologica Scandinavica 95, no. 12 (2016): 1359–1367. 27622859 10.1111/aogs.13017

