# Supplementary material for: Evaluating the Cost‐Effectiveness of Antenatal Screening for Major Structural Anomalies During the First Trimester of Pregnancy: A Decision Model
Source: BJOG. 2025 Jan 21;132(5):638–47. doi: 10.1111/1471-0528.18053 (PMC11879910; doi:10.1111/1471-0528.18053)
Supplement: Supplementary file 1 — Data S1. [file BJO-132-638-s001.docx]

**Supplementary file accompanying paper:**

**Evaluating the cost-effectiveness of antenatal screening for major structural anomalies during the first trimester of pregnancy: a decision model. Campbell HE, Karim J, Papageorgiou AT, Wilson E, Rivero-Arias O for the ACCEPTS Study.**

**Table of Contents**

[1. Model development and assumptions 3](#_Toc184288851)

[2. Screening outcome sub-trees 4](#_Toc184288852)

[2.1 Genetic testing sub-tree 4](#_Toc184288853)

[2.2 First trimester true positive screen (T1 TP) sub-tree 5](#_Toc184288854)

[2.3 First trimester false negative screen (T1 FN) sub-tree 6](#_Toc184288855)

[2.4 First trimester false positive screen (T1 FP) sub-tree 7](#_Toc184288856)

[2.5 First trimester true negative screen (T1 TN) sub-tree 8](#_Toc184288857)

[3. Maternal Markov models 8](#_Toc184288858)

[4. Decision tree event probabilities 10](#_Toc184288859)

[4.1 Anomaly prevalences 10](#_Toc184288860)

[4.2 First trimester screening outcome probabilities with current practice 11](#_Toc184288861)

[4.3 Event probabilities for the genetic testing sub-tree 13](#_Toc184288862)

[4.4 Event probabilities for the T1 TP pregnancy sub-tree 14](#_Toc184288863)

[4.5 Event probabilities for the T1 FN pregnancy sub-tree 17](#_Toc184288864)

[4.6 Event probabilities for the T1 FP pregnancy sub-tree 21](#_Toc184288865)

[4.7 Event probabilities for the T1 TN pregnancy sub-tree 22](#_Toc184288866)

[5. Markov model event probabilities 23](#_Toc184288867)

[6. Underlying utility levels for the decision tree 24](#_Toc184288868)

[7. Utility adjustments within the decision tree 24](#_Toc184288869)

[7.1 Utility adjustments for the T1 TP pregnancy sub-tree 25](#_Toc184288870)

[7.2 Utility adjustments for the T1 FN pregnancy sub-tree 27](#_Toc184288871)

[7.3 Utility adjustments for the T1 FP pregnancy sub-tree 29](#_Toc184288872)

[7.4 Utility adjustments for the T1 TN pregnancy sub-tree 35](#_Toc184288873)

[8. Utility adjustments within the maternal Markov models 39](#_Toc184288874)

[8.1 Live birth maternal Markov models 39](#_Toc184288875)

[8.2 Stillbirth maternal Markov model 45](#_Toc184288876)

[8.3 Second trimester termination maternal Markov model 48](#_Toc184288877)

[8.4 Second trimester fetal loss with genetic testing maternal Markov model 48](#_Toc184288878)

[8.5 Spontaneous miscarriage maternal Markov model 48](#_Toc184288879)

[8.6 First trimester termination maternal Markov model 49](#_Toc184288880)

[8.7 First trimester fetal loss with genetic testing maternal Markov model 49](#_Toc184288881)

[9. Costs for the decision tree 49](#_Toc184288882)

[10. Costs for the maternal Markov models 53](#_Toc184288883)

[10.1 Live birth maternal Markov models 53](#_Toc184288884)

[10.2 Stillbirth maternal Markov model 56](#_Toc184288885)

[10.3 Second trimester termination maternal Markov model 56](#_Toc184288886)

[10.4 Second trimester fetal loss with genetic testing maternal Markov model 56](#_Toc184288887)

[10.5 Spontaneous miscarriage maternal Markov model 57](#_Toc184288888)

[10.6 First trimester termination maternal Markov model 57](#_Toc184288889)

[10.7 First trimester fetal loss with genetic testing maternal Markov model 57](#_Toc184288890)

[11. Implementing first trimester anomaly screening in the model 57](#_Toc184288891)

[11.1 First trimester screening outcome probabilities with the protocol 57](#_Toc184288892)

[11.2 Additional screening costs 60](#_Toc184288893)

[11.3 Utility impact 61](#_Toc184288894)

[12. Deterministic Sensitivity Analyses 61](#_Toc184288895)

[13. Value of Information (VoI) Analysis 64](#_Toc184288896)

[14. Infant Markov models 67](#_Toc184288897)

[15. Members of the Assessing Clinical and Cost Effectiveness of Prenatal first-Trimester anomaly Screening (ACCEPTS) group 69](#_Toc184288898)

[16. References 69](#_Toc184288899)

1. **Model development and assumptions**

In developing the model, various structures were considered, including one using combined anomaly prevalence and screening performance statistics. However, findings from the project’s systematic reviews of first anomaly trimester screening demonstrated anomaly heterogeneity with regards to screening characteristics, fetal outcomes, genetic associations, permanence, and clinical management.^1, 2^ A decision was therefore made to model pregnancy pathways by individual anomaly type.

A decision was also taken to assume fetuses would present with only a single anomaly; a scarcity of published data on multiple malformations would make it challenging to accurately model combinations of anomalies and the order of their detection.

We also recognized that the first trimester screening options for genetic syndromes and for structural fetal anomalies are inextricably linked. Many fetuses diagnosed with Down’s, Edwards’ and Patau’s syndromes after the concurrent combined screening test will be subsequently found to have a structural anomaly (for example, a cardiac anomaly) and vice-versa. The model was designed so that the finding of a structural anomaly with a strong genetic association at any gestational age would lead to the offer of genetic testing to parents. However, we were unable to model the potential impact of a high-risk combined screening test on the early identification of structural fetal anomalies.

To avoid further increasing the size of the model, we modelled a true positive screen as one identified by a sonographer and subsequently confirmed by a fetal medicine specialist. Additionally, women with a ‘sonographer false positive (FP)’ screen that was subsequently corrected by a fetal medicine specialist, were classified as having a true negative finding within the model and incurred only the cost of an additional fetal medicine scan. Only when correction of the screening error does not occur, a false positive screen (termed a ‘fetal medicine FP’ within the model) remains and directs subsequent care / decisions.

Finally, the work that preceded the health economic modelling component of the project identified ectopia cordis as one of the eight major anomalies for inclusion in the protocol. We instead modelled the broader category of major cardiac anomalies, which are amongst the most common structural congenital anomaly in low risk populations (4 per 1000 fetuses) and encompass ectopia cordis.^3-5^ The commonly used definition of a major cardiac abnormality as a malformation assumed to be lethal, or requiring surgery or interventional cardiac catheterization during the first year of life, was followed. Holoprosencephaly was included in the model as the ‘alobar’ sub-type; the most common and severe form of the anomaly.

1. **Screening outcome sub-trees**

For each of the screening outcomes (true positive, false negative, false positive, and true negative) a sub-tree modelled the occurrence of subsequent events. The structure of each sub-tree was standardised across the different anomalies (albeit populated with anomaly-specific event probabilities). A genetic testing sub-tree was also developed to model the genetic testing pathway for women whose fetuses screen positive for a structural anomaly with a strong genetic association. Each sub-tree is illustrated below.

- 1. *Genetic testing sub-tree*

Following a positive screening result (during either trimester), women are offered a genetic test that they accept or decline (see Figure S1). We assumed testing with chorionic villus sampling during the first trimester and amniocentesis during the second trimester. We assumed the finding of a structural anomaly with a known genetic association would be akin to receiving a very high chance combined screening test result and thus have the same high genetic testing acceptance rates.^6^ Women who declined genetic testing were assumed to have a euploid fetus.

**Figure S1 - Genetic diagnostic testing sub-tree for women screening positive for a structural anomaly with a strong genetic association.**


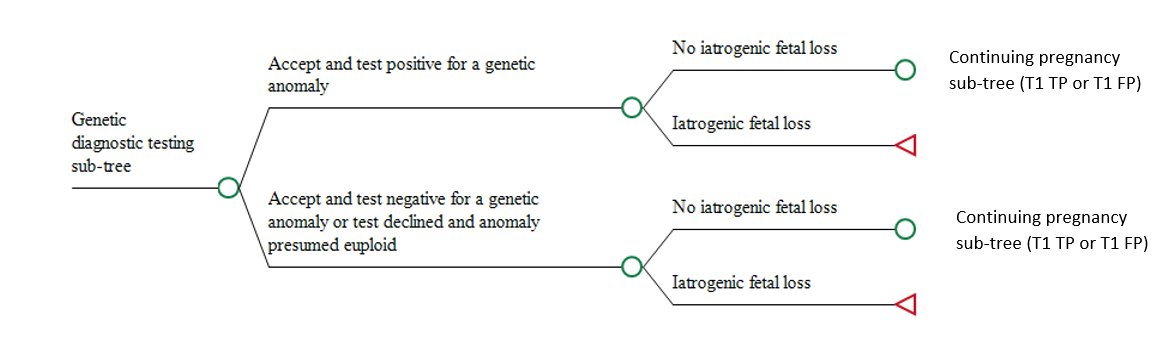


Abbreviations: T1 first trimester, TP true positive, FP false positive.

A red triangle denotes end of tree pathway

- 1. *First trimester true positive screen (T1 TP) sub-tree*

Figure S2 shows the true positive screening sub-tree replicated for each anomaly. Women screening positive for an anomaly without a genetic association enter this sub-tree following their first trimester screening result. Women screening positive for an anomaly with a genetic association, first enter the genetic testing sub-tree. These women are stratified based upon whether the genetic test was positive or negative (or unknown), and then subsequently enter this sub-tree if not suffering a fetal loss.

**Figure S2 First trimester true positive screen sub-tree**


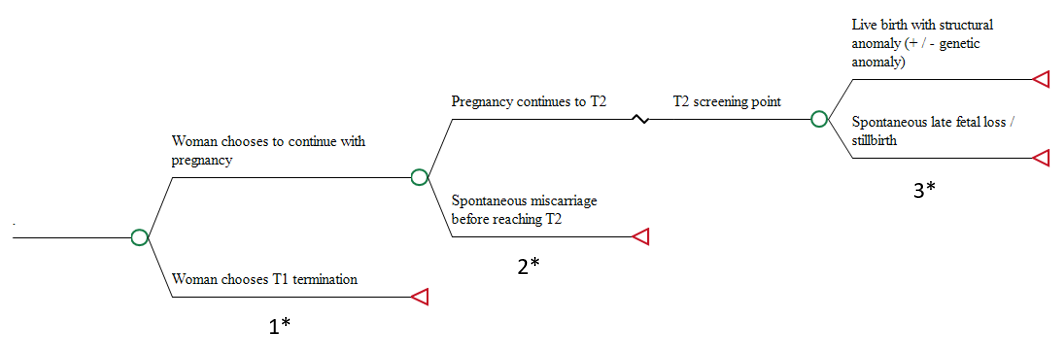


Abbreviations: T1 first trimester, T2 second trimester,

A red triangle denotes end of tree pathway

*Probabilities for numbered events are shown in Table S4

The model assumes that if a woman chose not to terminate her pregnancy during the first trimester, she would not choose a termination during the second trimester, even if she received further information about her baby’s condition.

- 1. *First trimester false negative screen (T1 FN) sub-tree*

The first trimester false negative screening sub-tree (Figure S3) is also replicated for each anomaly. Women who undergo routine second trimester anomaly screening, may have their baby’s anomaly detected, may undergo genetic testing, and will make decisions around the continuation of their pregnancy, or, the anomaly may remain undetected (a further false negative finding).

**Figure S3 First trimester false negative screen sub-tree**


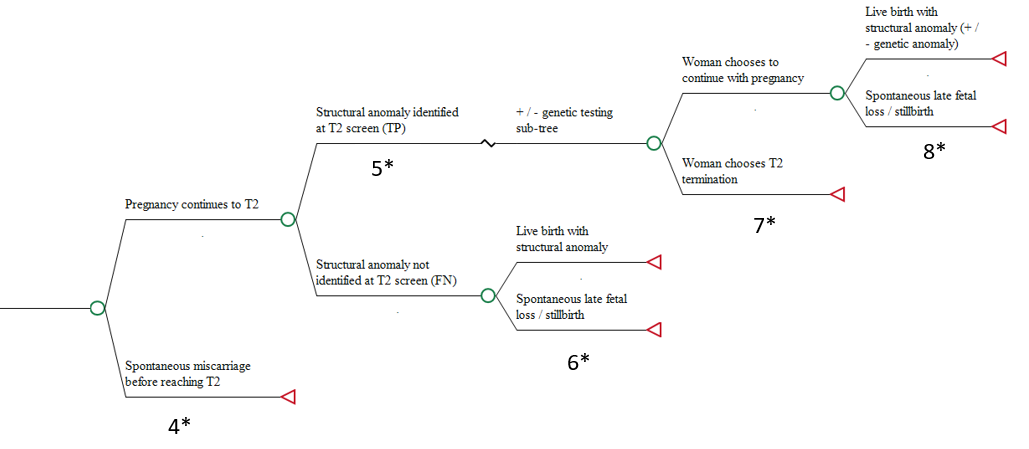


Abbreviations: T2 second trimester, TP true positive, FN false negative

*Probabilities for numbered events are shown in Table S5

- 1. *First trimester false positive screen (T1 FP) sub-tree*

Figure S4 shows the false positive screening sub-tree. This pathway features in the model only once and combines the first trimester false positives for all anomalies in the protocol. Women with false positive screens for anomalies with a genetic association, enter the sub-tree after first passing through the genetic testing sub-tree.

**Figure S4 First trimester false positive screen sub-tree**

**
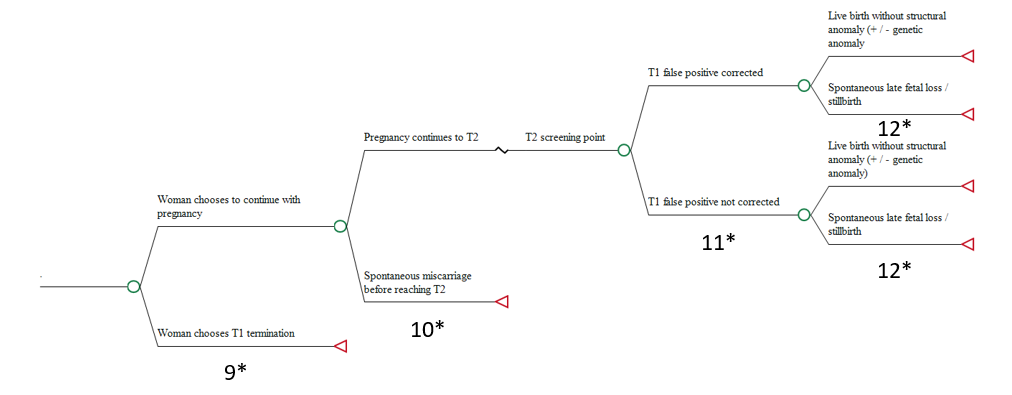
**

Abbreviations: T1 first trimester, T2 second trimester, TP true positive, FN false negative

*Probabilities for numbered events are shown in Table S6

At the point of second trimester anomaly screening, when women may or may not have their FP screening finding corrected by further fetal medicine screening. Again it is assumed that women choosing not to terminate their pregnancies during the first trimester following the (false) positive screening result (and any genetic diagnostic testing), would not choose a termination during the second trimester if the error remained uncorrected.

- 1. *First trimester true negative screen (T1 TN) sub-tree*

Figure S5 shows the first trimester true negative screening sub-tree which also features in the model only once. In the second trimester, a small proportion of women may have a structural anomaly erroneously identified by a sonographer (second trimester sonographer false positive result). This error may be corrected by a fetal medicine specialist, but if left uncorrected, a woman may make subsequent decisions about her pregnancy on the assumption that her baby has an anomaly.

**Figure S5 First trimester true negative screen sub-tree
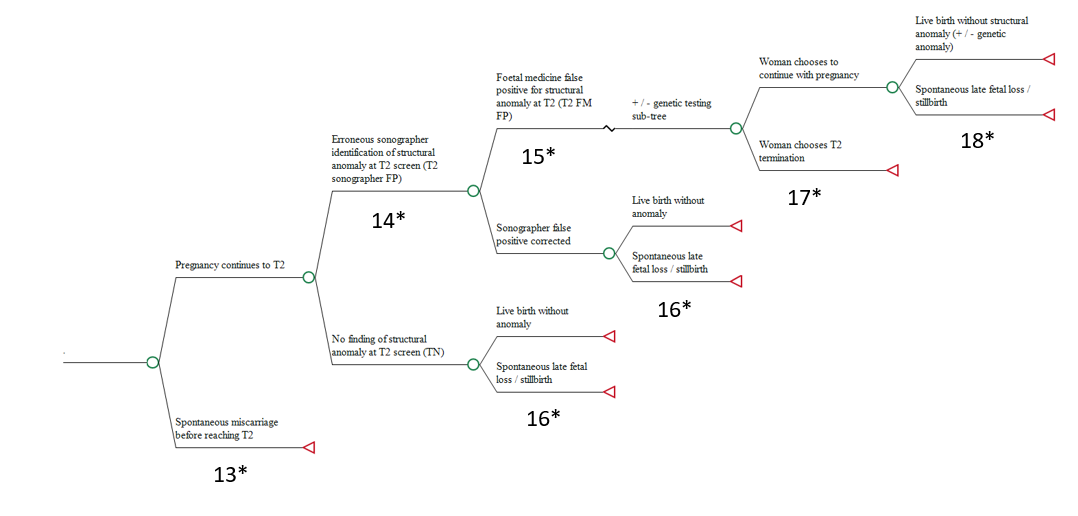
**

Abbreviations: T2 second trimester, FP false positive, TN true negative, FM fetal medicine

*Probabilities for numbered events are shown in Table S7

1. **Maternal Markov models**

Individual Markov models simulated the longer-term implications for mothers of each of the pregnancy outcomes in the decision tree model (Figure 1 panel (b) in the main paper).

For live births with an anomaly, maternal Markov models were developed for each anomaly type (with and without an accompanying genetic anomaly where appropriate). Models were also developed for mothers of infants born without a structural anomaly and with a genetic anomaly alone. In total, 14 maternal Markov models were constructed.

Each Markov model used the same two state structure shown in Figure S6. Women enter the model in the alive health state and each subsequent year (over a period of 20 years), can remain here or move to the deceased health state. Each year women accrue costs and QALYs related to their pregnancy outcome. QALYs are calculated by adjusting underlying levels of maternal quality of life for the ongoing negative psychological symptoms experienced following the pregnancy outcomes. Further, and because a mother’s level of wellbeing is intrinsically linked to that of her baby, for women with a live birth, the corresponding maternal Markov models linked maternal quality of life over time to the prognosis and morbidity likely to be experienced by the infants.

Costs included in the maternal Markov models were for the mental health care women may receive for the negative psychological symptoms arising as a result of their pregnancy outcome.

**Figure S6 Maternal Markov model structure**


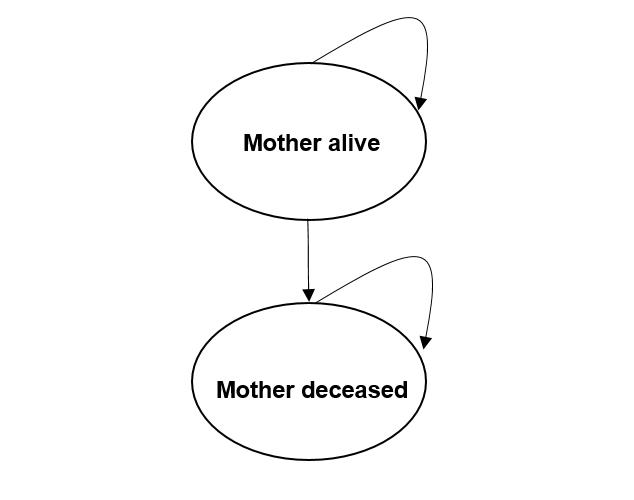


1. **Decision tree event probabilities**
   1. *Anomaly prevalences*

The first trimester prevalence estimates for each structural anomaly were informed by the project’s systematic reviews and are shown in Table S1 below.^1, 2^

**Table S1 First trimester anomaly prevalence estimates used in the model**

| **Anomaly** | **Prevalence**  **(95% CI)** | **Distribution type and parameters*** | **Source** |
| --- | --- | --- | --- |
| Anencephaly | 0.000887  (0.000678 to 0.001125) | Beta, α=60.48, β=68190.23 -α | Karim et al.2024^1^ |
| Body stalk anomaly | 0.000185  (0.00009 to 0.000314 | Beta, α=9.51, β=51378.38 -α | Karim et al.2024^1^ |
| Major cardiac anomalies | 0.0041  (0.0039 to 0.0043) | Beta, α=1607.80, β=392147.16 -α | Karim et al.2022^2^ |
| Encephalocele | 0.000155  (0.000096 to 0.000228) | Beta, α=21.15, β=136458.75 -α | Karim et al.2024^1^ |
| Exomphalos / Omphalocele | 0.000674  (0.000496 to 0.000879) | Beta, α=47.27, β=70130.79 -α | Karim et al.2024^1^ |
| Holoprosencephaly | 0.000342  (0.000215 to 0.0005) | Beta, α=22.122, β=64684.77 -α | Karim et al.2024^1^ |
| Gastroschisis | 0.000300  (0.000236 to 0.000372) | Beta, α=73.45, β=244823.49 -α | Karim et al.2024^1^ |
| LUTO | 0.000334  (0.000296 to 0.000372) | Beta, α=290.29, β=869138.02 -α | Malin et al. 2012^7^ |

Abbreviations: CI confidence interval, LUTO lower urinary tract infection

*Estimated from mean and standard error using method of moments approach

One minus the sum of the prevalences gave the proportion of women whose babies were unaffected by any of the eight structural anomalies in the protocol (estimated to be 0.993).

## First trimester screening outcome probabilities with current practice

Table S2 shows current practice first trimester true positive probabilities for each anomaly. False positive rates for current practice are also shown.

As described previously, we distinguished between sonographer and fetal medicine false positives, with the latter being the proportion of sonographer false positives referred for specialist fetal medicine screening that remain uncorrected. We utilised both published studies and expert opinion, to determine these two separate false positive rates. Standard errors for each rates were informed by the ‘combined’ false positive rates within the literature.

When considering the false positive rates for exomphalos and for LUTO (identified during the first trimester via megacystis), we acknowledged that a significant proportion of these anomalies (in euploid fetuses) are known to spontaneously resolve as the pregnancy advances. At the time of first trimester screening, the “spontaneous resolution” of these anomalies cannot be predicted. In reality, a woman presenting with a euploid fetus and one of these anomalies, would be counselled regarding the high rate of spontaneous resolution. However, within the model, these cases were considered and costed as false positives.

**Table S2 First trimester screening outcomes with current practice**

| **Anomaly type,**  **Current practice T1 screening performance** | **Mean (SE)*** | **Distribution type and parameters** | **Source** |
| --- | --- | --- | --- |
| Major cardiac anomaly |  |  |  |
| T1 TP | 0.1351 (0.0373) | Beta, α=11.21, β=82.99 – α† | Karim et al. 2022^2^ |
| T1 FP – sonographer | 0.000032 (0.0000178) | Beta, α=3.23, β=100,993.12 – α† | Karim et al. 2022^2^ / expert opinion |
| T1 FP - fetal medicine | 0.00004 (0.0000178) | Beta, α=5.05, β=126,240.64 – α† | Karim et al. 2022^2^ / expert opinion |
| Acrania |  |  |  |
| T1 TP | 0.7037 (0.0213) | Beta, α=323,  β=459 – α | EUROCAT (UK regions, 2015-2019)^8^ |
| T1 FP – sonographer | 0.00001 (0.000005) | Beta, α=4.0, β=399,995 – α† | Karim et al. 2024^1^ / expert opinion |
| T1 FP - fetal medicine | 0.00000 | -- | Karim et al. 2024^1^ / expert opinion |
| Exomphalos |  |  |  |
| T1 TP | 0.5669 (0.0213) | Beta, α=305,  β=538 – α | EUROCAT (UK regions, 2015-2019)^8^ |
| T1 FP – sonographer‡ | 0.0018 (0.0000181) | Beta, α=9,872.00, β=5,484,446.97 – α† | Karim et al. 2024^1^ / expert opinion |
| T1 FP - fetal medicine‡ | 0.0015 (0.0000181) | Beta, α=6,857.62, β=4,571,745.89 – α† | Karim et al. 2024^1^ / expert opinion |
| Gastroschisis |  |  |  |
| T1 TP | 0.8335 (0.0124) | Beta, α=751,  β=901 – α | EUROCAT (UK regions, 2015-2019)^8^ |
| T1 FP – sonographer | 0.00002 (0.0000072) | Beta, α=7.72, β=385,793.75 – α† | Karim et al. 2024^1^ / expert opinion |
| T1 FP - fetal medicine | 0.000005 (0.0000072) | Beta, α=0.482, β=96,449.14 – α† | Karim et al. 2024^1^ / expert opinion |
| Alobar holoprosencephaly |  |  |  |
| T1 TP | 0.4196 (0.2477) | Beta, α=1.246,  β=2.97 – α† | Karim et al. 2024^1^ |
| T1 FP – sonographer | 0.00001 (0.000008) | Beta, α=1.56, β=156,247.44 – α† | Karim et al. 2024^1^ / expert opinion |
| T1 FP - fetal medicine | 0.000005 (0.000008) | Beta, α=0.391, β=78,123.61 – α† | Karim et al. 2024^1^ / expert opinion |
| Lower Urinary Tract Obstruction |  |  |  |
| T1 TP | 0.3307 (0.0500) | Beta, α=28.95, β=87.54 – α† | Karim et al. 2024^1^ / expert opinion |
| T1 FP – sonographer‡ | 0.0009 (0.000056) | Beta, α=285.06, β=286,730.51 – α† | Karim et al. 2024^1^ / expert opinion |
| T1 FP - fetal medicine‡ | 0.0005 (0.000056) | Beta, α=79.68, β=159,358.06 – α† | Karim et al. 2024^1^ / expert opinion |
| Encephalocele |  |  |  |
| T1 TP | 0.4495 (0.0373) | Beta, α=79.50, β=176.86 – α† | Karim et al. 2024^1^ / expert opinion |
| T1 FP – sonographer | 0.000005 (0.0000052) | Beta, α=0.925, β=184,909.32 – α† | Karim et al. 2024^1^ / expert opinion |
| T1 FP - fetal medicine | 0.000001 (0.0000052) | Beta, α=0.037, β=36,981.21 – α† | Karim et al. 2024^1^ / expert opinion |
| Body Stalk Anomaly |  |  |  |
| T1 TP | 0.9851 (0.0109) | Beta, α=120.72, β=122.54 – α† | Karim et al. 2024^1^ / expert opinion |
| T1 FP – sonographer | 0.00001 (0.0000065) | Beta, α=2.37, β=236,683.02 – α† | Karim et al. 2024^1^ / expert opinion |
| T1 FP - fetal medicine | 0.00000 | - | Karim et al. 2024^1^ / expert opinion |

Abbreviations: T1 first trimester, TP true positive, FP false positive, SE standard error

*Standard errors could only be estimated for the FP rate of screening overall (sonographer plus fetal medicine FPs) for each anomaly. These estimates were thus used to reflect uncertainty around both sonographer and fetal medicine FP rates.

†Estimated from mean and standard error using methods of moments approach.

‡Anomaly is present at the time of screening but spontaneously resolves as the pregnancy progresses and so is considered a T1 false positive finding. The difference between T1 sonographer and T1 fetal medicine FPs is accounted for by some anomalies resolving between being identified by a sonographer and being screened by a fetal medicine specialist.

- 1. *Event probabilities for the genetic testing sub-tree*

Table S3 shows the event probabilities for the genetic testing sub-tree shown in Figure S1.

**Table S3 Event probabilities for the genetic testing sub-tree**

| **Description** | **Mean (SE)** | **Distribution type and parameters** | **Source** |
| --- | --- | --- | --- |
| Woman accepts invitation for genetic testing* | 0.8479 (0.019) | Beta, α=301, β=355-α | Spencer et al. 2003^6^ |
| Structural anomaly is accompanied by a genetic anomaly in T1: |  |  |  |
| Major cardiac anomaly | 0.2132 (0.010)† | Beta, α=363, β=1,703-α | NCARDRS 2019 Report^5^ |
| Exomphalos/Omphalocele | 0.4965 (0.060) | Beta, α=34.3, β=69.1-α‡ | Karim et al. 2024^1^ |
| Alobar holoprosencephaly | 0.7838 (0.067) | Beta, α=29, β=37-α | Syngelaki et al. 2017^9^ |
| LUTO (and megacystis) | 0.2069 (0.034) | Beta, α=30, β=145-α | Liao et al. 2003^10^ |
| Encephalocele | 0.1407 (0.021)† | Beta, α=37, β=263-α | EUROCAT 2011-2018^4^ |
| Structural anomaly is accompanied by a genetic anomaly in T2: |  |  |  |
| Major cardiac anomaly | 0.2132 (0.010)† | Beta, α=363, β=1,703-α | NCARDRS 2019 Report^5^ |
| Exomphalos/Omphalocele | 0.3832 (0.016) | Beta, α=338, β=882-α | EUROCAT UK 2011-18^4^ |
| Alobar holoprosencephaly | 0.4421 (0.027) | Beta, α=149, β=337-α | EUROCAT UK 2011-18^4^ |
| LUTO | 0.1600 (0.036) | Beta, α=16, β=100-α | Malin et al. 2012^7^ |
| Encephalocele | 0.1407 (0.021)† | Beta, α=37, β=263-α | EUROCAT 2011-2018^4^ |
| A genetic anomaly is present without a structural anomaly | 0.0040 (0.00005) | Beta, α=7,977, β=1,982,731-α | NCARDRS 2018 Report^11^ |
| Genetic testing results in a fetal loss in T1 | 0.0020 (0.001)§ | Beta, α=2.3, β=1,127.4-α‡ | Salomon et al. 2019^12^ |
| Genetic testing results in a fetal loss in T2 | 0.0030 (0.001) | Beta, α=9.5, β=3,177.9-α‡ | Salomon et al. 2019^12^ |

Abbreviations: T1 First trimester, T2 Second trimester, SE standard error, LUTO Lower Urinary Tract Obstruction.

*Based on screening risk of >1:150.

†Probability of accompanying genetic anomaly assumed to be same in first and second trimesters given lack of informative data.

‡Estimated from mean and standard error using method of moments approach.

§SE estimated from reported confidence interval after setting lower bound to zero.

## Event probabilities for the T1 TP pregnancy sub-tree

Table S4 shows the event probabilities for the first trimester true positive screen sub-tree shown in Figure S2. These probabilities are the same for both arms of the model.

**Table S4 Event probabilities for the first trimester true positive screen sub-tree**

| **Event probability number* and description** | **Mean (SE)** | **Distribution type and parameters** | **Source** |
| --- | --- | --- | --- |
| **[1] First trimester termination following a true positive screening result** | | | |
| Major cardiac anomaly |  |  |  |
| With genetic anomaly | 0.8732 (0.0392) | Beta α=62, β=71 - α | Hartge et al. 2012^13^, Eleftheariades et al. 2012^14^, Orlandi et al. 2014^15^, Sainz et al. 2018^16^ |
| Without genetic   anomaly† | 0.7080 (0.0426) | Beta α=80, β=113 - α | Minnella et al. 2020^17^ |
| Acrania | 1.0000 | n/N = 82/82 | Syngelaki et al. 2011^18^  Grande 2012^19^  Liao 2021^20^ |
| Exomphalos/Omphalocele |  |  |  |
| With genetic anomaly | 0.7929 (0.0220) | Beta α=268, β=338 - α | EUROCAT 2011-2018^4^ |
| Without genetic   anomaly† | 0.1582 (0.0591) | Beta α=5.87, β=37.1 - α‡ | Karim et al. 2024^1^ |
| Gastroschisis | 0.0526 (0.0499) | Beta α=1, β=19 - α | Syngelaki et al. 2011^18^ |
| Alobar holoprosencephaly |  |  |  |
| With genetic anomaly | 1.0000 | n/N = 15/15 | Kagan et al. 2010^21^ |
| Without genetic   anomaly† | 1.0000 | n/N = 29/29 | Kagan et al. 2010^21^ |
| LUTO |  |  |  |
| With genetic anomaly | 0.8667 (0.0611) | Beta α=26, β=30 - α | Liao et al. 2003^10^ |
| Without genetic   anomaly† | 0.3684 (0.0450) | Beta α=42, β=114- α | Liao et al. 2003^10^ |
| Encephalocele |  |  |  |
| With genetic anomaly | 0.7838 (0.0668) | Beta α=29, β=37 - α | EUROCAT 2011-2018^4^ |
| Without genetic   anomaly† | 0.7434 (0.0290) | Beta α=168, β=226 - α | EUROCAT 2011-2018^4^ |
| Body Stalk Anomaly | 0.9747 (0.0176) | Beta α=77, β=79 - α | Syngelaki 2011^18^  Liao 2021^20^  Murphy 2011^22^  Daskalakis 1997^23^ |
| **[2] Spontaneous miscarriage before the second trimester, in women with a true positive screening result who chose to continue with their pregnancy§** | | | |
| Major cardiac anomaly |  |  |  |
| With genetic anomaly | 0.8571 (0.1237) | Beta α=6, β=7 - α | Hartge et al. 2012^13^ |
| Without genetic   anomaly† | 0.2727 (0.0764) | Beta α=9, β=33 - α | Minnella et al. 2020^17^ |
| Acrania | NA** | -- | -- |
| Exomphalos/Omphalocele |  |  |  |
| With genetic anomaly | 0.1000 (0.0060) | Beta α=249.9, β=2,499 - α‡ | Morris 2008^24^/ expert opinion |
| Without genetic   anomaly† | 0.0500 (0.0279) | Beta α=3, β=60 - α | Kagan et al. 2010^21^ |
| Gastroschisis | 0.0058 (0.00059)†† | Beta α=96.07, β=16,564.24 - α‡ | Expert opinion / Salomon et al. 2019^12^ |
| Alobar holoprosencephaly |  |  |  |
| With genetic anomaly | NA** | -- | -- |
| Without genetic   anomaly† | NA** | -- | -- |
| LUTO |  |  |  |
| With genetic anomaly | 0.1158 (0.0064)‡‡ | Beta α=290, β=2,505 - α | Expert opinion / EUROCAT 2011-2018^4^ |
| Without genetic   anomaly† | 0.1111 (0.0368) | Beta α=8, β=72 - α | Liao et al. 2003^10^ |
| Encephalocele |  |  |  |
| With genetic anomaly | 0.1158 (0.0064)‡‡ | Beta α=290, β=2,505 - α | Expert opinion / EUROCAT 2011-2018^4^ |
| Without genetic   anomaly† | 0.0058 (0.00059)†† | Beta α=96.07, β=16,564.24 - α‡ | Expert opinion / Salomon et al. 2019^12^ |
| Body Stalk Anomaly | 0.5000 (0.0500) | Beta α=49.5, β=99 - α‡ | Expert Opinion |
| **[3] Stillbirth in women with a true positive screening result who chose to continue with their pregnancy and did not suffer a spontaneous miscarriage§** | | | |
| Major cardiac anomaly |  |  |  |
| With genetic anomaly | 0.1048 (0.0211) | Beta α=22, β=210 - α | Garne et al. 2001^25^ |
| Without genetic   anomaly† | 0.0149 (0.0030) | Beta α=24, β=1,614 - α | Garne et al. 2001^25^ |
| Acrania | NA** | -- |  |
| Exomphalos/Omphalocele |  |  |  |
| With genetic anomaly | 0.3000 (0.0544) | Beta α=21, β=70 - α | EUROCAT 2011-2018^4^ |
| Without genetic   anomaly† | 0.1107 (0.0194) | Beta α=29, β=262 - α | EUROCAT 2011-2018^4^ |
| Gastroschisis | 0.0293 (0.0065) | Beta α=20, β=683 - α | EUROCAT 2011-2018^4^ |
| Alobar holoprosencephaly |  |  |  |
| With genetic anomaly | NA** | -- | -- |
| Without genetic   anomaly† | NA** | -- | -- |
| LUTO |  |  |  |
| With genetic anomaly | 1.0000 | n/N = 4/4 | Liao et al. 2003^10^ |
| Without genetic   anomaly† | 0.1864 (0.0357) | Beta α=22, β=118 - α | Malin et al. 2012^7^ |
| Encephalocele |  |  |  |
| With genetic anomaly | 0.1250 (0.1102) | Beta α=1, β=8 - α | EUROCAT 2011-2018^4^ |
| Without genetic   anomaly† | 0.1207 (0.0424) | Beta α=7, β=58 - α | EUROCAT 2011-2018^4^ |
| Body Stalk Anomaly | 1.0000 | n/N = 2/2 | Syngelaki 2011^18^  Liao 2020^20^  Murphy 2011^22^  Daskalakis 1997^23^ |

Abbreviations: SE Standard error, NA Not applicable, LUTO Lower Urinary Tract Obstruction.

*Event probability numbers correspond to those in Figure S2.

†Women not genetically tested were assumed to have a euploid fetus and the same termination rate as women with a negative genetic test result.

‡Estimated from mean and standard error using method of moments approach.

§Probabilities are conditioned upon preceding events.

**All pregnancies affected by this anomaly were terminated during the first trimester.

††In the absence of data and informed by expert opinion, the miscarriage rate was assumed to be as for the general population of pregnant women.

‡‡In the absence of informative data, the miscarriage rate was assumed to be driven by the co-existing genetic anomaly.

- 1. *Event probabilities for the T1 FN pregnancy sub-tree*

Table S5 shows the event probabilities for the first trimester false negative screen sub-tree shown in Figure S3. These probabilities are the same for both arms of the model.

**Table S5 Event probabilities for the first trimester false negative screen sub-tree**

| **Event probability number* and description** | **Mean (SE)** | **Distribution type** | **Parameters** | **Source** |
| --- | --- | --- | --- | --- |
| **[4] Spontaneous miscarriage before reaching the second trimester, in women whose anomaly went undiagnosed during the first trimester** | | | | |
| Major cardiac anomaly |  |  |  |  |
| With genetic anomaly | 0.0845 (0.0328) | Beta | α=6, β=71 - α | Hartge et al. 2012^13^, Eleftheriades et al. 2012^14^, Orlandi et al. 2014^15^, Sainz et al. 2018^16^ |
| Without genetic   anomaly | 0.0796 (0.0254) | Beta | α=9, β=113 - α | Minnella et al. 2020^17^ |
| Acrania | 0.0058 (0.00059)† | Beta | α=96.07, β=16,564.24 - α‡ | Salomon et al. 2019^12^ |
| Exomphalos/Omphalocele |  |  |  |  |
| With genetic anomaly | 0.1000 (0.0060) | Beta | α=249.9, β=2,499 - α‡ | Morris 2008^24^/ expert opinion |
| Without genetic   anomaly | 0.0500 (0.0279) | Beta | α=3, β=60 - α | Kagan et al. 2010^21^ |
| Gastroschisis | 0.0058 (0.00059)† | Beta | α=96.07, β=16,564.24 - α‡ | Salomon et al. 2019^12^ |
| Alobar holoprosencephaly |  |  |  |  |
| With genetic anomaly | 0.1158 (0.0064)§ | Beta | α=290, β=2,505 - α | EUROCAT 2011-2018^4^ |
| Without genetic   anomaly | 0.0058 (0.00059)† | Beta | α=96.07, β=16,564.24 - α‡ | Salomon et al. 2019^12^ |
| LUTO |  |  |  |  |
| With genetic anomaly | 0.1158 (0.0064)§ | Beta | α=290, β=2,505 - α | EUROCAT 2011-2018^4^ |
| Without genetic   anomaly | 0.1111 (0.0368) | Beta | α=8, β=72 - α | Liao et al. 2003^10^ |
| Encephalocele |  |  |  |  |
| With genetic anomaly | 0.1158 (0.0064)§ | Beta | α=290, β=2,505 - α | EUROCAT 2011-2018^4^ |
| Without genetic   anomaly | 0.0058 (0.00059)† | Beta | α=96.07, β=16,564.24 - α‡ | Salomon et al. 2019^12^ |
| Body Stalk Anomaly | 0.5000 (0.0500) | Beta | α=49.5, β=99 - α‡ | Expert opinion |
| **[5] True positive second trimester screening result in women whose anomaly went undiagnosed during the first trimester** | | | | |
| Major cardiac anomaly | 0.5000 (0.0069) | Beta | α=2,593, β=5,186 - α | EUROCAT 2015-2019^8^ |
| Acrania | 0.9541 (0.0070) | Beta | α=853, β=894 - α | EUROCAT 2015-2019^8^ |
| Exomphalos/Omphalocele | 0.9853 (0.0051) | Beta | α=538, β=546 - α | EUROCAT 2015-2019^8^ |
| Gastroschisis | 0.9772 (0.0049) | Beta | α=901, β=922 - α | EUROCAT 2015-2019^8^ |
| Alobar holoprosencephaly | 0.9175 (0.0251) | Beta | α=109.32, β=119.15 - α‡ | Karim et al. 2024^1^ |
| LUTO | 0.5070 (0.0296) | Beta | α=144, β=284 - α | Malin et al. 2012^7^ |
| Encephalocele | 0.8994 (0.0365) | Beta | α=60.18, β=66.91 - α‡ | Karim et al. 2024^1^ |
| Body Stalk Anomaly | 0.9851 (0.0109) | Beta | α=120.72, β=122.54 - α‡ | Karim et al. 2024^1^ |
| **[6] Stillbirth in women whose anomaly went undiagnosed during the first trimester and who then received a false negative screening result during the second trimester**** | | | | |
| Major cardiac anomaly | As in Table S4†† | As in Table S4†† | As in Table S4†† | As in Table S4†† |
| Acrania | 0.4937 (0.0559) | Beta | α=39, β=79 - α | EUROCAT 2011-2018^4^ |
| Exomphalos/Omphalocele | As in Table S4†† | As in Table S4†† | As in Table S4†† | As in Table S4†† |
| Gastroschisis | As in Table S4 | As in Table S4 | As in Table S4 | As in Table S4 |
| Alobar holoprosencephaly (Without genetic anomaly) | 0.1818 (0.0575) | Beta | α=8, β=44 - α | EUROCAT 2011-2018^4^ |
| LUTO | As in Table S4†† | As in Table S4†† | As in Table S4†† | As in Table S4†† |
| Encephalocele | As in Table S4†† | As in Table S4†† | As in Table S4†† | As in Table S4†† |
| Body Stalk Anomaly | As in Table S4 | As in Table S4 | As in Table S4 | As in Table S4 |
| **[7] Second trimester termination in women whose anomaly remained undetected during the first trimester but then who received a true positive screening result during the second trimester** | | | | |
| Major cardiac anomaly |  |  |  |  |
| With genetic anomaly | 0.8049 (0.0276) | Beta | α=165, β=205 - α | Garne et al. 2005^26^ |
| Without genetic   anomaly | 0.2952 (0.0277) | Beta | α=80, β=271 - α | Garne et al. 2005^26^ |
| Acrania | 0.9241 (0.0082) | Beta | α=962, β=1,041 - α | EUROCAT 2011-2018^4^ |
| Exomphalos/Omphalocele |  |  |  |  |
| With genetic anomaly | 0.7929 (0.0220) | Beta | α=268, β=338 - α | EUROCAT 2011-2018^4^ |
| Without genetic   anomaly | 0.5184 (0.0214) | Beta | α=282, β=544 - α | EUROCAT 2011-2018^4^ |
| Gastroschisis | 0.0869 (0.0103) | Beta | α=65, β=748 - α | EUROCAT 2011-2018^4^ |
| Alobar holoprosencephaly |  |  |  |  |
| With genetic anomaly | 0.9060 (0.0238) | Beta | α=135, β=149 - α | EUROCAT 2011-2018^4^ |
| Without genetic   anomaly | 0.7660 (0.0308) | Beta | α=144, β=188 - α | EUROCAT 2011-2018^4^ |
| LUTO |  |  |  |  |
| With genetic anomaly | 0.8667 (0.0611) | Beta | α=26, β=30 - α | Liao 2003^10^ |
| Without genetic   anomaly | 0.3723 (0.0352) | Beta | α=70, β=188 - α | Malin et al. 2012^7^ |
| Encephalocele |  |  |  |  |
| With genetic anomaly | 0.7838 (0.0668) | Beta | α=29, β=37 - α | EUROCAT 2011-2018^4^ |
| Without genetic   anomaly | 0.7434 (0.0290) | Beta | α=168, β=226 - α | EUROCAT 2011-2018^4^ |
| Body Stalk Anomaly | 0.9747 (0.0176) | Beta | α=77, β=79 - α | Syngelaki 2011^18^  Liao 2021^20^  Murphy 2011^22^  Daskalakis 1997^23^ |
| **[8] Stillbirth in women whose anomaly remained undetected during the first trimester but who then received a true positive screening result during the second trimester and chose to continue with their pregnancy** | | | | |
| Major cardiac anomaly |  |  |  |  |
| With genetic anomaly | As in Table S4 | As in Table S4 | As in Table S4 | As in Table S4 |
| Without genetic   anomaly | As in Table S4 | As in Table S4 | As in Table S4 | As in Table S4 |
| Acrania | 0.4937 (0.0559) | Beta | α=39, β=79 - α | EUROCAT 2011-2018^4^ |
| Exomphalos/Omphalocele |  |  |  |  |
| With genetic anomaly | As in Table S4 | As in Table S4 | As in Table S4 | As in Table S4 |
| Without genetic   anomaly | As in Table S4 | As in Table S4 | As in Table S4 | As in Table S4 |
| Gastroschisis | As in Table S4 | As in Table S4 | As in Table S4 | As in Table S4 |
| Alobar holoprosencephaly |  |  |  |  |
| With genetic anomaly | 0.4286 (0.1278) | Beta | α=6, β=14 - α | EUROCAT 2011-2018^4^ |
| Without genetic   anomaly | 0.1818 (0.0575) | Beta | α=8, β=44 - α | EUROCAT 2011-2018^4^ |
| LUTO |  |  |  |  |
| With genetic anomaly | As in Table S4 | As in Table S4 | As in Table S4 | As in Table S4 |
| Without genetic   anomaly | As in Table S4 | As in Table S4 | As in Table S4 | As in Table S4 |
| Encephalocele |  |  |  |  |
| With genetic anomaly | As in Table S4 | As in Table S4 | As in Table S4 | As in Table S4 |
| Without genetic   anomaly | As in Table S4 | As in Table S4 | As in Table S4 | As in Table S4 |
| Body Stalk Anomaly | As in Table S4 | As in Table S4 | As in Table S4 | As in Table S4 |

Abbreviations: SE Standard error, LUTO Lower Urinary Tract Obstruction.

*Event probability numbers correspond to those in Figure S3.

†In the absence of data and informed by expert opinion, the miscarriage rate was assumed to be as for the general population of pregnant women.

‡Estimated from mean and standard error using method of moments approach.

§In the absence of data, the miscarriage rate was assumed to be driven by the co-existing genetic anomaly.

**All anomalies not detected in the first or second trimester were assumed not to have any accompanying genetic anomaly.

††Relevant value from Table S4 is that for structural anomaly without a genetic anomaly.

- 1. *Event probabilities for the T1 FP pregnancy sub-tree*

Table S6 shows the event probabilities for the first trimester false positive screen sub-tree shown in Figure S4. These probabilities are the same for both arms of the model.

**Table S6** **Event probabilities for the first trimester false positive screen sub-tree**

| **Event probability number* and description** | **Mean (SE)** | **Distribution type** | **Parameters** | **Source** |
| --- | --- | --- | --- | --- |
| **[9] First trimester termination following a false positive screening result** | | | | |
| For each structural anomaly (+ / - a genetic anomaly) see Table S4† | See Table S4 | See Table S4 | See Table S4 | See Table S4 |
| **[10] Spontaneous miscarriage before the second trimester, in women with a false positive structural anomaly screening result who chose to continue with their pregnancy** | | | | |
| With genetic anomaly | 0.1158 (0.0064) | Beta | α=290, β=2,505 - α | EUROCAT 2011-2018^4^ |
| Without genetic anomaly | 0.0058 (0.00059) | Beta | α=96.07, β=16,564.24 - α‡ | Salomon et al. 2019^12^ |
| **[11] First trimester false positive screen for a structural anomaly is corrected at the second trimester screening point, in women who chose to continue with their pregnancy and did not suffer a spontaneous miscarriage§** | | | | |
| Major cardiac anomaly | 0.9500 (0.005) | Beta | α=1,804.05, β=1,899 - α‡ | Expert opinion |
| Acrania | NA** | -- | -- | -- |
| Exomphalos/Omphalocele | 0.9250 (0.005) | Beta | α=2,565.95, β=2,774 - α‡ | Kagan 2010^21^/ expert opinion |
| Gastroschisis | 1.0000 | -- | -- | Expert opinion |
| Alobar holoprosencephaly | NA†† | -- | -- | -- |
| LUTO | 0.9000 (0.005) | Beta | α=3,239.1, β=3,599 - α‡ | Kagan 2010^21^/ expert opinion |
| Encephalocele | 1.0000 | -- | -- | Expert opinion |
| Body Stalk Anomaly | NA** | -- | -- | Expert opinion |
| **[12] Stillbirth in women with a false positive screening result who chose to continue with their pregnancy and did not suffer a spontaneous miscarriage** | | | | |
| With genetic anomaly | 0.0711‡‡ | Log-normal | Mean=2.8160, SE=0.0856 | EUROCAT 2011-2018^4^ |
| Without genetic anomaly | 0.0046 (0.00008) | Beta | α=3,368, β= 738,332 - α | Draper et al. 2019^27^ |

Abbreviations: SE standard error, NA not applicable, LUTO Lower Urinary Tract Obstruction.

*Event probability numbers correspond to those in Figure S4.

†For each model arm (and + / - a genetic anomaly) weighted average first trimester termination probabilities were estimated by combining the proportion of fetal medicine FPs accounted for by each anomaly (data in Tables S2 and S21) and corresponding anomaly-specific first trimester termination probabilities (Table S4).

‡Estimated from mean and standard error using method of moments approach.

§For each model arm (and + / - a genetic anomaly) weighted average second trimester FP correction probabilities were estimated by combining data on the proportion of FP screens for each anomaly type reaching this point with their corresponding second trimester correction probabilities.

**All sonographer false positives for these anomalies are corrected by fetal medicine specialists in the first trimester.

††All pregnancies thought to be affected by this structural anomaly were assumed to be terminated during the first trimester. ‡‡Estimated by applying an odds ratio for the increased risk of stillbirth with Down’s syndrome (shown in adjacent columns) to the underlying odds of a stillbirth in the general population (calculated using data from the row immediately below).

- 1. *Event probabilities for the T1 TN pregnancy sub-tree*

Table S7 shows the event probabilities for the first trimester true negative screen sub-tree shown in Figure S5. These probabilities are the same for both arms of the model.

**Table S7** **Event probabilities for the first trimester true negative screen sub-tree**

| **Event probability number* and description** | **Mean (SE)** | **Distribution type** | **Parameters** | **Source** |
| --- | --- | --- | --- | --- |
| **[13] Spontaneous miscarriage before the second trimester, in women without a structural anomaly and a true negative finding (with screening) or no finding (with current practice)** | | | | |
| Spontaneous miscarriage | 0.0058 (0.00059) | Beta | α=96.07, β=16,564.24 - α† | Salomon et al. 2019^12^ |
| **[14] Second trimester sonographer false positive screen for a structural anomaly in women who did not suffer a spontaneous miscarriage‡** | | | | |
| Major cardiac anomaly | 0.0211 (0.0014) | Beta | α=210, β=9,959 - α | Expert opinion |
| Acrania | 0.0000 | -- | -- | Karim et al. 2024^1^ / expert opinion |
| Exomphalos/Omphalocele | 0.0010 (0.0010) | Beta | α=1, β=1,000 - α | Kagan 2010^21^ / expert opinion |
| Gastroschisis | 0.00002 (0.00004) | Beta | α=0.2, β=10,000 - α | Karim et al. 2024^1^ / expert opinion |
| Alobar holoprosencephaly | 0.00002 (0.00004) | Beta | α=0.2, β=10,000 - α | Karim et al. 2024^1^ / expert opinion |
| LUTO | 0.00002 (0.00004) | Beta | α=0.2, β=10,000 - α | Karim et al. 2024^1^ / expert opinion |
| Encephalocele | 0.00001 (0.00003) | Beta | α=0.1, β=10,000 - α | Karim et al. 2024^1^ / expert opinion |
| Body Stalk Anomaly | 0.00001 (0.00003) | Beta | α=0.1, β=9,998 - α | Karim et al. 2024^1^ / expert opinion |
| **[15] Second trimester fetal medicine false positive screen in women who did not suffer a spontaneous miscarriage and who received a second trimester sonographer false positive screen and presented to the fetal medicine clinic§** | | | | |
| Major cardiac anomaly | 0.0004 (0.0002) | Beta | α=4, β=10,000 - α | Karim et al. 2022^2^ / expert opinion |
| Acrania | NA | -- | -- | -- |
| Exomphalos/Omphalocele | 1.0000 | -- | -- | Kagan 2010^21^ / expert opinion |
| Gastroschisis | 0.0000 | -- | -- | Expert opinion |
| Alobar holoprosencephaly | 0.0000 | -- | -- | Expert opinion |
| LUTO | 0.0000 | -- | -- | Expert opinion |
| Encephalocele | 0.0000 | -- | -- | Expert opinion |
| Body Stalk Anomaly | 0.0000 | -- | -- | Expert opinion |
| **[16] Stillbirth in women with a second trimester true negative anomaly screening result and those for whom a false positive sonographer screen is correct by fetal medicine** | | | | |
| General population risk | 0.0046 (0.00008) | Beta | α=3,368, β= 738,332 - α | Draper et al. 2019^27^ |
| **[17] Second trimester termination in women without an anomaly but who received a fetal medicine false positive screening result following routine second trimester anomaly screening**** | | | | |
| Second trimester termination probabilities relevant only for major cardiac anomaly and exomphalos - see Table S5 | See Table S5 | See Table S5 | See Table S5 | See Table S5 |
| **[18] Stillbirth in women with a second trimester false positive structural anomaly screening result and who chose to continue with their pregnancy** | | | | |
| With genetic anomaly | 0.0711†† | Log-normal | Mean=2.8160, SE=0.0856 | EUROCAT 2011-2018^4^ |
| Without genetic anomaly | 0.0046  (0.00008) | Beta | α=3,368, β= 738,332 - α | Draper et al. 2019^27^ |

Abbreviations: SE standard error, NA not applicable.

*Event probability numbers correspond to those in Figure S5.

†Estimated from mean and standard error using method of moments approach.

‡Entered in the model as the sum of the second trimester sonographer false positives for each anomaly.

§Combined with the second trimester sonographer false positives in the table to estimate a weighted average second trimester fetal medicine false positive probability.

**Weighted average second trimester termination probability estimated.

††Estimated by applying an odds ratio for the increased risk of stillbirth with Down’s syndrome (shown in adjacent columns) to the underlying odds of a stillbirth in the general population (calculated using data from the row immediately below).

1. **Markov model event probabilities**

Age and gender matched lifetable data were used to model annual maternal mortality risks across all Markov models.^28^ For women delivering a live baby with an anomaly, annual mortality was adjusted to reflect the increased mortality risk observed for mothers raising a child with a congenital anomaly.^29^ Table S8 shows these data.

**Table S8 Annual maternal mortality risks and adjustments within the Markov models**

| **Parameter** | **Mean (SE)** | **Distribution type** | **Parameters** | **Source** |
| --- | --- | --- | --- | --- |
| *Underlying maternal mortality* | | | | |
| Underlying annual mortality risk | Age and sex adjusted life table data | -- | -- | ONS 2017-2019^28^ |
| *Adjustments following a live birth of a baby with an anomaly* | | | | |
| Increased risk (hazard ratio) of mortality in mothers of children with major congenital anomalies. Years 0-10 | 1.27 (0.13) | Log Normal | Mean=0.239 SE=0.103 | Fuller et al. 2021^29^ |
| Increased risk (hazard ratio) of mortality in mothers of children with major congenital anomalies. Years 11-20 | 1.25 (0.10) | Log Normal | Mean=0.223  SE=0.078 | Fuller et al. 2021^29^ |

Abbreviation: ONS Office for National Statistics

# Underlying utility levels for the decision tree

Women’s underlying utility levels were assumed to match the UK three-level EQ-5D index population norms for females aged 25 to 34^30^. This parameter was entered into the model using a beta distribution with a mean of 0.93 and a standard error of 0.007.

# Utility adjustments within the decision tree

In adjusting underlying quality of life for the events women experience in the different first trimester screening outcome sub-trees (Figures S2 to S5), two key assumptions were made. Firstly, following a positive screen (true or a false positive) we assumed the utility impact to be the same, regardless of the structural anomaly identified. Secondly, for structural anomalies accompanied by a genetic anomaly, we assumed the latter would become of primary concern to a woman and so modelled the maternal quality of life impact of a diagnosis of a genetic anomaly.

Each sequence of events modelled was given an identifying code combining a sequence number (e.g. S1) and the pregnancy trimester at which the sequence occurred (e.g. T1). The following tables show the utility adjustments made. Unless otherwise stated, all adjustments were entered into the model using beta distributions.

## Utility adjustments for the T1 TP pregnancy sub-tree

Table S9 shows the utility adjustments within the first trimester true positive screening sub-tree (Figure S2). First trimester event sequences are described in the first column, with associated utility adjustments (both utility multipliers and decrements were used to reduce underlying utility) in the second column. For women whose pregnancy ended during the first trimester through termination or following genetic testing, the associated utility decrement was assumed to persist for their remaining time within the decision tree. This was also true for women continuing with a pregnancy following a first trimester true positive diagnosis, unless they experienced a spontaneous miscarriage or a stillbirth. Miscarriage was assumed to occur at 16 weeks’ gestation and stillbirth, at 30 weeks’ gestation. For these women, we used the utility adjustments in Table S9 until the fetal loss, and then switched to using a utility adjustment for the miscarriage / stillbirth. Tables S10 and S11 show the miscarriage and stillbirth utility adjustments.

**Table S9 Utility adjustments used within the T1 TP pregnancy sub-tree**

| **Sequence No.**  **Screening / testing outcomes and reproductive decisions at T1** | **T1 Mean (SE) utility adjustment** | **Source of T1 utility adjustment** | **Sequence No.**  **Screening / testing outcomes and reproductive decisions at T2** | **T2 Mean (SE) utility adjustment** |
| --- | --- | --- | --- | --- |
| S1T1.  Screen +ve for structural anomaly (TP), test +ve for a genetic anomaly, continue with pregnancy | 0.655*  (0.021) | Kuppermann et al. 2016^31^ | NA  Outcomes / decisions unchanged from T1 | Unchanged from T1 |
| S2T1.  Screen +ve for structural anomaly (TP), test +ve for a genetic anomaly, terminate pregnancy | 0.772*  (0.021) | Kuppermann et al. 2016^31^ | -- | -- |
| S3T1.  Screen +ve for structural anomaly (TP), test +ve for a genetic anomaly, fetal loss due to testing | 0.744*  (0.021) | Kuppermann et al. 2016^31^ | -- | -- |
| S4T1.  Screen +ve for structural anomaly (TP), test –ve for a genetic anomaly (or fetus presumed euploid), continue with pregnancy | 0.272†  (0.033) | Kuppermann et al. 2016^31^, Kaasen et al. 2017^32^, Sobocki et al. 2007^33^ | NA  Outcomes / decisions unchanged from T1 | Unchanged from T1 |
| S5T1.  Screen +ve for structural anomaly (TP), test –ve for a genetic anomaly (or fetus presumed euploid), terminate pregnancy | 0.156†  (0.019) | Kuppermann et al.^31^ Kaasen et al. 2017^32^, Sobocki et al. 2007^33^ | -- | -- |
| S6T1.  Screen +ve for structural anomaly (TP), test –ve for a genetic anomaly (or fetus presumed euploid), fetal loss due to testing | 0.183†  (0.022) | Kuppermann et al.^31^ Kaasen et al. 2017^32^, Sobocki et al. 2007^33^ | -- | -- |

Abbreviations: T1 first trimester, T2 second trimester, SE standard error, TP true positive, +ve positive, -ve negative, NA not applicable.

*Used as a multiplier to adjust underlying utility levels

†Used as a decrement to adjust underlying utility levels. Decrements were estimated in a series of steps using the sources shown. Details available from the authors upon request.

**Table S10 Estimated maternal impact of miscarriage**

| **Description** | **Mean (SE)** | **Distribution type** | **Parameters** | **Source** |
| --- | --- | --- | --- | --- |
| Proportion of women experiencing moderate anxiety / depression in the six months following a miscarriage | 0.3000 (0.035) | Beta | α = 51.13, β = 170.43 – α* | Farren et al. 2018^34^,  Author Assumption |
| Utility decrement - moderate anxiety / depression | 0.355 (0.033) | Beta | α = 74.29, β = 209.26 – α* | Kind et al. 1999^30^, Sobocki et al. 2007^33^ |

Abbreviation: SE standard error

*Estimated from the mean and standard error using the methods of moments approach.

Informed by a systematic review, we inferred that 30% of women suffering a miscarriage would experience moderate levels of anxiety and depression in the short-term.^34^ Using EQ-5D data from a cohort of Swedish individuals with moderate depression in a primary care setting, we estimated a utility decrement of 0.355 (SE=0.033) (see Table S10).^33^ We applied this decrement to the proportion of women suffering with moderate anxiety / depression following a miscarriage for their remaining 24 weeks in the decision tree.

**Table S11 Estimated maternal impact of stillbirth**

| **Description** | **Mean (SE)** | **Distribution type** | **Parameters** | **Source** |
| --- | --- | --- | --- | --- |
| Proportion of women experiencing moderate anxiety / depression immediately following a stillbirth | 0.581 (0.0227) | Beta | α=275, β=473-α | Inferred using Redshaw et al. 2014^35^ and Heazell et al. 2016^36^ |
| Utility decrement - moderate anxiety / depression | 0.355 (0.033) | Beta | α = 74.29, β = 209.26 – α* | Kind et al.1999^30^, Sobocki et al. 2007^33^ |
| Utility decrement - mild anxiety / depression | 0.250 (0.027) | Beta | α = 64.05, β = 256.20 – α* | Kind et al.1999 ^30^, Sobocki et al. 2007^33^ |

Abbreviation: SE standard error

*Estimated from the mean and standard error using the methods of moments approach.

Informed by the literature, we inferred that 58% of women suffering a stillbirth at 30 weeks would suffer with moderate levels of depression during their remaining 10 weeks in the decision tree (see Table S11).^35, 36^ For these women we assumed a utility decrement of 0.355 (SE=0.033) estimated as for miscarriage. For the remaining 42% of women we assumed mild levels of depression and a utility decrement of 0.250 (SE=0.027) estimated using EQ-5D data from the same Swedish study.^33^

## Utility adjustments for the T1 FN pregnancy sub-tree

Table S12 shows the utility adjustments used within the first trimester false negative sub-tree. Adjustments varied depending upon whether the first trimester anomaly screening protocol had been implemented (scenario S7T1) or not (scenario S8T1). For the former, where a negative (albeit a false negative) anomaly screening finding is reported to the woman, published evidence suggests this will have reassuring qualities.^37-40^ We were guided by expert opinion, and implemented a small reassurance utility increment (0.01) using a uniform distribution with minimum and maximum values of 0 and 0.02 respectively. As data suggest the reassuring qualities are likely to be transient, we assumed the effect to last only until the point of second trimester anomaly screening (eight weeks later) or until the point of miscarriage if this occurred prior to a woman reaching the second trimester.

Sequence S8T1 in Table S12 had no associated utility adjustment and was used in the current practice arm and for women declining the invitation for first trimester anomaly screening.

A false negative first trimester anomaly screen (S7T1) or indeed no screen and finding (S8T1) may be altered during the second trimester. These sequences could be followed in the second trimester by any one of the range of true positive sequences first shown in Table S9 (now denoted S1T2 to S6T2 to reflect the second trimester screening point). For those true positive sequences ending with a pregnancy termination (S2T2 and S5T2) or a fetal loss following genetic testing (S3T2 and S6T2), a further utility decrement was added to reflect the increased levels of distress experienced by women suffering pregnancy loss during the second trimester.^41, 42^ We estimated this utility decrement with a uniform distribution and minimum and maximum values of 0.01 and 0.044 respectively.^43-46^ The utility adjustments for sequences S1T2 to S6T2 in Table S12 were assigned to women from the point of second trimester screening until the end of the decision tree (a duration of 20 weeks). Women continuing with their pregnancy and suffering a stillbirth had their utility adjusted as described above. Women with a second false negative screening finding (S7T2) received a further 8-week reassurance utility increment.

## Utility adjustments for the T1 FP pregnancy sub-tree

Women receiving a first trimester fetal medicine false positive screen (sequences S9T1 to S14T1 in Table S13), followed the same first trimester sequences and thus experienced the same levels of quality of life as women receiving a true positive first trimester screening finding (sequences S1T1 to S6T1 in Table S9).

For women receiving a genetic anomaly diagnosis following a false positive first trimester anomaly screen (sequence S9T1), we maintained the utility decrement for the remainder of the pregnancy because the presence of the genetic anomaly was assumed to continue to negatively affect a woman’s utility.

For women without a concomitant diagnosis of a genetic anomaly (S12T1), and whose first trimester false positive screening result is corrected by second trimester screening (S10T2 in Table S13), we removed the utility decrement from the first trimester but assumed women’s quality of life would not return to normal levels. After correction of a false positive screening result, women can continue to experience slightly elevated levels of anxiety and so we implemented a small utility decrement of 0.006 for the remainder of the pregnancy.^31, 47, 48^ We assumed this value to be the midpoint of a uniform distribution with minimum and maximum values of 0 and 0.012 respectively.

**Table S12** **Utility adjustments used within the T1 FN pregnancy sub-tree**

| **US screening outcome at T1** | **Sequence No.**  **Screening / testing outcomes and reproductive decisions at T1** | **T1 Mean (SE) utility adjustment** | **US screening outcome at T2** | **Sequence No.**  **Screening / testing outcomes and reproductive decisions at T2** | **T2 Mean (SE) utility adjustment** |
| --- | --- | --- | --- | --- | --- |
| **False negative finding** | S7T1.  Screen –ve for structural anomaly (FN), continue with pregnancy | Uniform distribution with min=0 and max=0.02* | **True positive finding** | S1T2.  Screen +ve for structural anomaly (TP), test +ve for a genetic anomaly, continue with pregnancy | As for S1T1 (see Table S9) |
|  |  |  |  | S2T2.  Screen +ve for structural anomaly (TP), test +ve for a genetic anomaly, terminate pregnancy | As for S2T1 (see Table S9) less decrement based on uniform distribution with min=0.01 and max=0.044† |
|  |  |  |  | S3T2.  Screen +ve for structural anomaly (TP), test +ve for a genetic anomaly, fetal loss due to testing | As for S3T1 (see Table S9) less decrement based on uniform distribution with min=0.01 and max=0.044† |
|  |  |  |  | S4T2.  Screen +ve for structural anomaly (TP), test –ve for a genetic anomaly (or fetus presumed euploid), continue with pregnancy | As for S4T1 (see Table S9) |
|  |  |  |  | S5T2.  Screen +ve for structural anomaly (TP), test –ve for a genetic anomaly (or fetus presumed euploid), terminate pregnancy | As for S5T1 (see Table S9) less decrement based on uniform distribution with min=0.01 and max=0.044† |
|  |  |  |  | S6T2.  Screen +ve for structural anomaly (TP), test –ve for a genetic anomaly (or fetus presumed euploid), fetal loss due to testing | As for S6T1 (see Table S9) less decrement based on uniform distribution with min=0.01 and max=0.044† |
|  |  |  | **False negative finding** | S7T2.  Screen –ve for structural anomaly (FN), continue with pregnancy | Uniform distribution with min=0 and max=0.02* |
| **No finding** | S8T1. No anomaly screening protocol is implemented, structural anomaly is not identified | 0.000 | **True positive finding** | S1T2.  Screen +ve for structural anomaly (TP), test +ve for a genetic anomaly, continue with pregnancy | As for S1T1 (see Table S9) |
|  |  |  |  | S2T2.  Screen +ve for structural anomaly (TP), test +ve for a genetic anomaly, terminate pregnancy | As for S2T1 (see Table S9) less decrement based on uniform distribution with min=0.01 and max=0.044† |
|  |  |  |  | S3T2.  Screen +ve for structural anomaly (TP), test +ve for a genetic anomaly, fetal loss due to testing | As for S3T1 (see Table S9) less decrement based on uniform distribution with min=0.01 and max=0.044† |
|  |  |  |  | S4T2.  Screen +ve for structural anomaly (TP), test –ve for a genetic anomaly (or fetus presumed euploid), continue with pregnancy | As for S4T1 (see Table S9) |
|  |  |  |  | S5T2.  Screen +ve for structural anomaly (TP), test –ve for a genetic anomaly (or fetus presumed euploid), terminate pregnancy | As for S5T1 (see Table S9) less decrement based on uniform distribution with min=0.01 and max=0.044† |
|  |  |  |  | S6T2.  Screen +ve for structural anomaly (TP), test –ve for a genetic anomaly (or fetus presumed euploid), fetal loss due to testing | As for S6T1 (see Table S9) less decrement based on uniform distribution with min=0.01 and max=0.044† |
|  |  |  | **False negative finding** | S7T2.  Screen –ve for structural anomaly (FN), continue with pregnancy | Uniform distribution with min=0 and max=0.02* |

Abbreviations: T1 first trimester, T2 second trimester, FN false negative, TP true positive, +ve positive, -ve negative

*Utility increment associated with reassurance from a negative anomaly scan (applied for eight weeks). Informed by expert opinion

†Additional utility decrement associated with second trimester termination / second trimester fetal loss from genetic testing.^43-46^

**Table S13 Utility adjustments used within the T1 FP pregnancy sub-tree**

| **US screening outcome at T1** | **Sequence No.**  **Screening / testing outcomes and reproductive decisions at T1** | **T1 Mean (SE) utility adjustment** | **US screening outcome at T2** | **Sequence No.**  **Screening / testing outcomes and reproductive decisions at T2** | **T2 Mean (SE) utility adjustment** |
| --- | --- | --- | --- | --- | --- |
| **False positive finding** | S9T1.  Screen +ve for structural anomaly (FP), test +ve for a genetic anomaly, continue with pregnancy | As for S1T1  (see Table S9) | **True negative finding** | S8T2.  Screen –ve for structural anomaly (TN), known genetic anomaly, continue with pregnancy | Unchanged from T1 |
|  |  |  | **False positive finding** | S9T2.  Screen +ve for structural anomaly (FP), known genetic anomaly, continue with pregnancy | Unchanged from T1 |
|  | S10T1.  Screen +ve for structural anomaly (FP), test +ve for a genetic anomaly, terminate pregnancy | As for S2T1  (see Table S9) | -- | -- | -- |
|  | S11T1.  Screen +ve for structural anomaly (FP), test +ve for a genetic anomaly, fetal loss due to testing | As for S3T1  (see Table S9) | -- | -- | -- |
|  | S12T1.  Screen +ve for structural anomaly (FP), test –ve for a genetic anomaly (or fetus presumed euploid), continue with pregnancy | As for S4T1  (see Table S9) | **True negative finding** | S10T2.  Screen –ve for structural anomaly (TN), previously tested negative for genetic anomaly (or fetus presumed euploid), continue with pregnancy | Population norm less decrement based on a uniform distribution with min=0 and max=0.012* |
|  |  |  | **False positive finding** | S11T2.  Screen +ve for structural anomaly (FP) previously tested negative for genetic anomaly (or fetus presumed euploid), continue with pregnancy | Unchanged from T1 |
|  | S13T1.  Screen +ve for structural anomaly (FP), test –ve for a genetic anomaly (or fetus presumed euploid), terminate pregnancy | As for S5T1  (see Table S9) |  | -- | -- |
|  | S14T1.  Screen +ve for structural anomaly (FP), test –ve for a genetic anomaly (or fetus presumed euploid), fetal loss due to testing | As for S6T1  (see Table S9) |  | -- | -- |

Abbreviations: T1 first trimester, T2 second trimester, SE standard error, FP false positive, TN true negative, +ve positive, -ve negative

*Ongoing utility decrement following correction of a first trimester false positive screening result.^31^

## Utility adjustments for the T1 TN pregnancy sub-tree

We applied the eight-week ‘reassurance’ utility increment to women receiving a true negative result on their first trimester anomaly screen (S15T1 in Table S14). For women without a structural anomaly and no formal first trimester anomaly screening and no false incidental findings (scenario S16T1 in Table S14), we made no adjustment to underlying utility levels.

With second trimester anomaly screening, and as shown in Table S14, some women may receive a fetal medicine false positive finding, (S9T2 and S11T2 to S15T2). Utility levels for these women were adjusted for the various scenario outcomes with the same utility decrements and multipliers used for women with a true positive finding during the second trimester (scenarios S1T2 to S6T2 in Table S12). Most women without an anomaly however would receive a true negative diagnosis and were thus assigned the reassurance increment for the next eight weeks (S16T2 in Table S14).

**Table S14 Utility adjustments used within the T1 TN pregnancy sub-tree**

| **US screening outcome at T1** | **Screening / testing outcomes and reproductive decisions at T1** | **T1 Mean (SE) utility adjustment** | **US screening outcome at T2†** | **Screening / testing outcomes and reproductive decisions at T2** | **T2 Mean (SE) utility adjustment** |
| --- | --- | --- | --- | --- | --- |
| **True negative (TN) finding** | S15T1. Screen –ve for structural anomaly (TN), continue with pregnancy | Uniform distribution with min=0 and max=0.02* | **False positive (FP) finding** | S9T2. Screen +ve for structural anomaly (FP), test +ve for a genetic anomaly, continue with pregnancy | As for S1T2 (see Table S12) |
|  |  |  |  | S12T2. Screen +ve for structural anomaly (FP), test +ve for a genetic anomaly, terminate pregnancy | As for S2T2 (see Table S12) |
|  |  |  |  | S13T2. Screen +ve for structural anomaly (FP), test +ve for a genetic anomaly, iatrogenic loss due to testing | As for S3T2 (see Table S12) |
|  |  |  |  | S11T2. Screen +ve for structural anomaly (FP), test –ve for a genetic anomaly (or fetus presumed euploid), continue with pregnancy | As for S4T2 (see Table S12) |
|  |  |  |  | S14T2. Screen +ve for structural anomaly (FP), test –ve for a genetic anomaly (or fetus presumed euploid), terminate pregnancy | As for S5T2 (see Table S12) |
|  |  |  |  | S15T2. Screen +ve for structural anomaly (FP), test –ve for a genetic anomaly (or fetus presumed euploid), iatrogenic loss due to testing | As for S6T2 (see Table S12) |
|  |  |  | **True negative (TN) finding** | S16T2. Screen –ve for structural anomaly (TN), continue with pregnancy | Uniform distribution with min=0 and max=0.02* |
| **No finding** | S16T1. No anomaly screening protocol is implemented, so no true negative finding as such | 0.000 | **False positive (FP) finding** | S9T2. Screen +ve for structural anomaly (FP), test +ve for a genetic anomaly, continue with pregnancy | As for S1T2 (see Table S12) |
|  |  |  |  | S12T2. Screen +ve for structural anomaly (FP), test +ve for a genetic anomaly, terminate pregnancy | As for S2T2 (see Table S12) |
|  |  |  |  | S13T2. Screen +ve for structural anomaly (FP), test +ve for a genetic anomaly, iatrogenic loss due to testing | As for S3T2 (see Table S12) |
|  |  |  |  | S11T2. Screen +ve for structural anomaly (FP), test –ve for a genetic anomaly (or fetus presumed euploid), continue with pregnancy | As for S4T2 (see Table S12) |
|  |  |  |  | S14T2. Screen +ve for structural anomaly (FP), test –ve for a genetic anomaly (or fetus presumed euploid), terminate pregnancy | As for S5T2 (see Table S12) |
|  |  |  |  | S15T2. Screen +ve for structural anomaly (FP), test –ve for a genetic anomaly (or fetus presumed euploid), iatrogenic loss due to testing | As for S6T2 (see Table S12) |
|  |  |  | **True negative (TN) finding** | S16T2. Screen –ve for structural anomaly (TN), continue with pregnancy | Uniform distribution with min=0 and max=0.02* |

Abbreviations: T1 first trimester, T2 second trimester, FP false positive, TN true negative, +ve positive, -ve negative

*Utility increment associated with reassurance from a negative anomaly scan (applied for eight weeks). Informed by expert opinion.

# Utility adjustments within the maternal Markov models

## Live birth maternal Markov models

We assumed that each year, a woman’s utility following a live birth would depend upon whether her baby had been born with an anomaly, and the implications of that anomaly for the child. Within each of the live birth maternal Markov models we utilised published infant survival data to determine the proportion of women expected to suffer the loss of their baby each year (see Table S15). Women receiving a diagnosis of body stalk anomaly were assumed to select termination or suffer a spontaneous fetal loss, and so a Markov model for this condition was not required (see Table S4).

Following a live birth, we modelled the maternal utility impact of an infant death during a given year and for a woman’s remaining time in the model. We estimated a decrement from underlying utility levels of 0.8649 for the first year following the loss of an infant (see Table S16).^30, 49-51^ A standard error for the decrement of 0.03 was assumed in the absence of variability / precision data.

Systematic reviews report variability in the proportions of women experiencing negative psychological consequences over time following the loss of a child.^52, 53^ We thus created a parameter to represent the duration (in years) required for a mother’s levels of quality of life to recover to close to underlying norm values. This parameter was entered into the model using a uniform distribution with minimum and maximum values of 5 and 20 years respectively (see Table S16). We assumed a constant annual rate of maternal utility improvement from the decrement observed immediately following the loss of the infant.

**Table S15 Annual mortality risks for infants born with and without various anomalies**

|  | **Annual probability of infant death** | | | | | | | | | | | | | | | |
| --- | --- | --- | --- | --- | --- | --- | --- | --- | --- | --- | --- | --- | --- | --- | --- | --- |
| **Year (Infant age)** | **Major cardiac anomaly*†** | **Source** | **Acrania** | **Source** | **Omphalocele (no genetic anomaly)** | **Source** | **Omphalocele (with genetic anomaly)** | **Source** | **Gastroschisis** | **Source** | **Alobar holoprosencephaly** | **Source** | **LUTO (no genetic anomaly)** | **Source** | **LUTO (with genetic anomaly)** | **Source** |
| 1 | 0.1578 | Estimated from data in Tennant et al. 2010^54^ | 1.000 | Baird and Sadovnick 1984^55^ | 0.0809 | Springett et al. 2014^56^ | 0.7333 | Springett et al. 2014^56^ | 0.0399 | Bradnock et al. 2011^57^ | 1.000 | Bullen et al. 2001^58^ | 0.2292 | Malin et al. 2012^7^{Malin, 2012 #43} | 0.2292 | Malin et al. 2012^7^ |
| 2 | 0.0072 |  | NA |  | 0.0002 | Office for National Statistics 2017-2019^28^ | 0.008 | Tennant et al. 2010^54^ | 0.0002 | Office for National Statistics 2017-2019^28^ | NA |  | 0.0002 | Office for National Statistics 2017-2019^28^ | 0.008 | Tennant et al. 2010^54^ |
| 3 | 0.0072 |  | NA |  | 0.0001 |  | 0.008 |  | 0.0001 |  | NA |  | 0.0001 |  | 0.008 |  |
| 4 | 0.0073 |  | NA |  | 0.0001 |  | 0.008 |  | 0.0001 |  | NA |  | 0.0001 |  | 0.008 |  |
| 5 | 0.0073 |  | NA |  | 0.0001 |  | 0.008 |  | 0.0001 |  | NA |  | 0.0001 |  | 0.008 |  |
| 6 | 0.0008 |  | NA |  | 0.0001 |  | 0.002 |  | 0.0001 |  | NA |  | 0.0001 |  | 0.002 |  |
| 7 | 0.0008 |  | NA |  | 0.0001 |  | 0.002 |  | 0.0001 |  | NA |  | 0.0001 |  | 0.002 |  |
| 8 | 0.0008 |  | NA |  | 0.0001 |  | 0.002 |  | 0.0001 |  | NA |  | 0.0001 |  | 0.002 |  |
| 9 | 0.0008 |  | NA |  | 0.0001 |  | 0.002 |  | 0.0001 |  | NA |  | 0.0001 |  | 0.002 |  |
| 10 | 0.0008 |  | NA |  | 0.0001 |  | 0.002 |  | 0.0001 |  | NA |  | 0.0001 |  | 0.002 |  |
| 11 | 0.0020 |  | NA |  | 0.0001 |  | 0.002 |  | 0.0001 |  | NA |  | 0.0001 |  | 0.002 |  |
| 12 | 0.0020 |  | NA |  | 0.0001 |  | 0.002 |  | 0.0001 |  | NA |  | 0.0001 |  | 0.002 |  |
| 13 | 0.0020 |  | NA |  | 0.0001 |  | 0.002 |  | 0.0001 |  | NA |  | 0.0001 |  | 0.002 |  |
| 14 | 0.0020 |  | NA |  | 0.0001 |  | 0.002 |  | 0.0001 |  | NA |  | 0.0001 |  | 0.002 |  |
| 15 | 0.0020 |  | NA |  | 0.0001 |  | 0.002 |  | 0.0001 |  | NA |  | 0.0001 |  | 0.002 |  |
| 16 | 0.0025 |  | NA |  | 0.0001 |  | 0.001 |  | 0.0001 |  | NA |  | 0.0001 |  | 0.001 |  |
| 17 | 0.0025 |  | NA |  | 0.0002 |  | 0.001 |  | 0.0002 |  | NA |  | 0.0002 |  | 0.001 |  |
| 18 | 0.0025 |  | NA |  | 0.0002 |  | 0.001 |  | 0.0002 |  | NA |  | 0.0002 |  | 0.001 |  |
| 19 | 0.0025 |  | NA |  | 0.0003 |  | 0.001 |  | 0.0003 |  | NA |  | 0.0003 |  | 0.001 |  |
| 20 | 0.0025 |  | NA |  | 0.0003 |  | 0.001 |  | 0.0003 |  | NA |  | 0.0003 |  | 0.001 |  |

**Table S15 continued**

|  | **Annual probability of infant death** | | | | | | | | | |
| --- | --- | --- | --- | --- | --- | --- | --- | --- | --- | --- |
| **Year (Infant age)** | **Encephalocele (no genetic anomaly)** | **Source** | **Encephalocele (with genetic anomaly)** | **Source** | **Body stalk anomaly** | **Source** | **Genetic anomaly only‡** | **Source** | **No congenital anomaly§** | **Source** |
| 1 | 0.3570 | Estimated from data in Tennant et al. 2010 and Office for National Statistics 2017-2019^28, 54^ | 0.3570 | Estimated from data in Tennant et al. 2010^54^ | NA | NA | 0.1715 | Estimated from data in Tennant et al. 2010^54^ | 0.0039 | Office for National Statistics 2017-2019^28^ |
| 2 | 0.0556 |  | 0.0556 |  | NA |  | 0.008 |  | 0.0002 |  |
| 3 | 0.0589 |  | 0.0589 |  | NA |  | 0.008 |  | 0.0001 |  |
| 4 | 0.0626 |  | 0.0626 |  | NA |  | 0.008 |  | 0.0001 |  |
| 5 | 0.0667 |  | 0.0667 |  | NA |  | 0.008 |  | 0.0001 |  |
| 6 | 0.0001 |  | 0.0022 |  | NA |  | 0.002 |  | 0.0001 |  |
| 7 | 0.0001 |  | 0.0022 |  | NA |  | 0.002 |  | 0.0001 |  |
| 8 | 0.0001 |  | 0.0022 |  | NA |  | 0.002 |  | 0.0001 |  |
| 9 | 0.0001 |  | 0.0022 |  | NA |  | 0.002 |  | 0.0001 |  |
| 10 | 0.0001 |  | 0.0022 |  | NA |  | 0.002 |  | 0.0001 |  |
| 11 | 0.0001 |  | 0.0020 |  | NA |  | 0.002 |  | 0.0001 |  |
| 12 | 0.0001 |  | 0.0020 |  | NA |  | 0.002 |  | 0.0001 |  |
| 13 | 0.0001 |  | 0.0020 |  | NA |  | 0.002 |  | 0.0001 |  |
| 14 | 0.0001 |  | 0.0020 |  | NA |  | 0.002 |  | 0.0001 |  |
| 15 | 0.0001 |  | 0.0020 |  | NA |  | 0.002 |  | 0.0001 |  |
| 16 | 0.0001 |  | 0.0008 |  | NA |  | 0.001 |  | 0.0001 |  |
| 17 | 0.0002 |  | 0.0008 |  | NA |  | 0.001 |  | 0.0002 |  |
| 18 | 0.0002 |  | 0.0008 |  | NA |  | 0.001 |  | 0.0002 |  |
| 19 | 0.0003 |  | 0.0008 |  | NA |  | 0.001 |  | 0.0003 |  |
| 20 | 0.0003 |  | 0.0008 |  | NA |  | 0.001 |  | 0.0003 |  |

Abbreviation: NA Not applicable

*Estimated by synthesising survival data reported by Tennant et al. for the main types of cardiac anomaly identified by NCARDS as ‘major’

†Also used to model mortality risk for infants born with this structural anomaly and a genetic anomaly

‡Based on survival data reported by Tennant et al. for infants born with a chromosomal anomaly.^54^

§Based upon national life table data reported by the Office for National Statistics.^28^

**Table S16 Utility-related parameters used to populate the live birth maternal Markov models**

| **Parameter** | **Mean (SE)** | **Distribution type** | **Parameters** | **Source** |
| --- | --- | --- | --- | --- |
| Decrement in maternal utility in the first year following the death of a child | 0.8649 (0.030) | Beta | α=111.43, β=128.83-α§ | Chung et al. 2021^49^  Odibo et al. 2006^50^  Song et al. 2010^59^ |
| Years taken for a mother’s utility to recover to underlying levels following the death of a child | 12.500 (4.317) | Uniform | Min = 5 years  Max = 20 years | Author assumption |
| For mothers with surviving infants: multiplier used to decrement maternal utility each year following the birth of an infant with a genetic anomaly (+ / - a structural anomaly) or with a neurodevelopmental disability | 0.621 (0.021) | Beta | α=330.80, β=532.69-α§ | Kuppermann et al. 2016^31^ |
| For mothers with surviving infants: multiplier used to decrement maternal utility during the first year of life of an infant with a structural anomaly only* | 0.670 (0.084) | Beta | α=20.32, β=30.34-α † | D’Souza et al. 2019^60^ |
| Proportion of infants with encephalocele affected by neurodevelopmental disability | 0.600 (0.0581) | Beta | α=42, β=70-α | Da Silva et al. 2015^61^ |
| Annual proportion of infants with LUTO developing end stage renal failure requiring dialysis and kidney transplantation during first six years of life | 0.056 | -- | -- | Biard et al. 2005^62^  Berte et al. 2018^63^ |
| Additional utility decrement for mothers whose infants are born with LUTO and develop renal failure requiring dialysis and kidney transplant | 0.060 (0.016) | Beta | α=13.16, β=219.31-α§ | Wu et al. 2020^64^ |
| Proportion of mothers with post-natal depression during the first year following the birth of a baby without an anomaly‡ | 0.078 (0.0029) | Beta | α=650, β=8,323-α | Heron et al. 2004^65^ |
| Utility decrement applied to women suffering post-natal depression in the years following the birth of an infant without an anomaly | 0.355 (0.033) | Beta | α=74.29, β=209.26-α§ | Kind et al. 1999^30^ / Sobocki et al. 2007^33^ |

Abbreviation: SE Standard Error

*The multiplier is increased by a constant amount in years 2 and 3, and by year 4 takes on a value of 1 (i.e. there is no further detrimental impact to underlying maternal utility levels).

†Parameters estimated from median and inter-quartile range.

‡Proportion is reduced each year at a constant rate such that after three years no women are suffering with depression.

§Estimated from the mean and standard error using the methods of moments approach.

For women whose babies survived each year, underlying utility was adjusted according to published evidence on the maternal quality of life impact of raising a child with a congenital anomaly.^66-68^ We considered the impact of each type of anomaly upon maternal utility as described below.

Of the five non-fatal anomalies, four have strong genetic associations (major cardiac anomaly, omphalocele, encephalocele, and LUTO). For women with these anomalies who were also diagnosed with a genetic anomaly, we assumed the genetic anomaly would exert the greater and more prolonged impact. For each year their child survived, maternal utility was decremented using a utility multiplier of 0.621 (SE=0.021) (row three of Table S16).^31^

The same four non-lethal anomalies can also occur without a genetic anomaly and in addition, gastroschisis is without a strong genetic association. As these anomalies are likely to be managed with corrective surgery soon after birth, we decremented maternal utility using a multiplier of 0.670 (SE=0.084) in the first year following birth (row four of Table S16). This accounted for the significantly increased risk of psychological problems that parents experience following their infant’s surgery.^67, 69^ Within the Markov models for isolated major cardiac anomaly, gastroschisis and omphalocele, the first year maternal utility decrement was reduced to zero (via means of an annual linear decline) by the end of year three to reflect the generally positive prognoses of these children following surgery, and thus the declining negative maternal impact.^54, 67, 70-73^

Amongst children born with an isolated encephalocele, as many as 60% will suffer developmental delay over the longer-term (row five of Table S16).^61, 74-76^ Within the isolated encephalocele Markov model, the underlying utility of these mothers was decremented using the same utility multiplier for mothers of babies with a genetic anomaly. For the remaining 40% of women, utility was modelled as for mothers of infants with an isolated major cardiac anomaly, an omphalocele, or gastroschisis.

Around one third of infants born with LUTO will likely develop chronic renal failure leading to end stage renal disease, renal dialysis and ultimately, kidney transplantation.^62, 63^ We assumed renal failure leading to end stage renal disease occurred at a constant annual rate over the first six years of the model (row 6 of Table S16). Utility for the mothers affected was reduced further using a utility decrement of 0.06 (SE=0.016) estimated using SF-12 responses from 54 parents of infants with rare genetic kidney diseases.^64^ As some children will suffer with ongoing morbidity following transplant, we maintained this decrement for the remainder of a woman’s time in the Markov model.^77^ For women whose infants did not develop renal failure, utility was modelled as for mothers of infants with an isolated major cardiac anomaly, an omphalocele, or gastroschisis.

For women whose children are born and survive each year with a genetic syndrome but no structural anomaly, underlying maternal utility levels were adjusted using the multiplier of 0.621 (SE=0.021). For women whose babies are unaffected by an anomaly, we assumed just under 8% would suffer with symptoms of depression following the birth of their baby.^65^ Underlying utility levels for these women were reduced using the utility decrement estimated for moderate depression (mean=0.355, SE=0.033).^33^ We reduced the proportion of women affected year on year, such that after three years, no women had symptoms.

## Stillbirth maternal Markov model

Table S17 shows the proportion of women experiencing differing levels of psychological symptoms and the associated maternal utility decrements used within the first year of the stillbirth maternal Markov model. Beyond the first year and to acknowledge that some women will recover in the years immediately following their stillbirth, whilst others will endure symptoms for longer, we decremented utility for all women between years 2 and 5 by applying the average utility multiplier shown in Table S17.

**Table S17 Utility-related parameters used to populate the maternal Markov models for pregnancy loss**

| **Event ending pregnancy**  **Parameters** | **Mean (SE)** | **Distribution type** | **Parameters** | **Source** |
| --- | --- | --- | --- | --- |
| *Stillbirth – first year*  Proportion of women with significant negative psychological symptoms* | 0.350 (0.030) | Beta | α=166, β=473-α† | Redshaw et al. 2014 ^35^, Heazell et al. 2016^36^ |
| Utility decrement with significant negative psychological symptoms | 0.355 (0.033) | Beta | α = 74.29, β = 209.26 – α‡ | Kind et al. 1999^30^, Sobocki et al. 2007^33^ |
| Utility decrement with negative psychological symptoms | 0.250 (0.027) | Beta | α = 64.05, β = 256.20 – α‡ | Kind et al.1999^30^, Sobocki P et al. 2007^33^ |
| *Stillbirth – years two to five* |  |  |  |  |
| Multiplier used to decrement utility for all women | 0.880 (0.011) | Beta | α = 767.12, β = 871.73 – α‡ | Kaimal et al. 2015^78^, Heazell et al. 2016^36^ |
| *Second trimester termination – first year* |  |  |  |  |
| Proportion of women with PTSD / negative psychological symptoms | 0.410 (0.011) | Beta | α=9, β=22-α† | Davies et al.2005^42^ |
| Multiplier used to decrement maternal utility for PTSD / negative psychological symptoms | 0.630 (0.013) | Beta | α = 868.32, β = 1378.29 – α‡ | Le et al. 2013^79^ |
| *Second trimester termination – years two to five* |  |  |  |  |
| Proportion of women with PTSD / negative psychological symptoms | See footnote§ | -- | -- | Author assumption |
| Multiplier used to decrement maternal utility for PTSD / negative psychological symptoms | 0.630 (0.013) | Beta | α = 868.32, β = 1378.29 – α‡ | Le et al. 2013^79^ |
| *Additional utility decrement pertaining to termination in the second trimester (years 1 to 20)* | 0.005 (0.003) | Uniform | Min=0.000, Max=0.010 | Author assumption |
| *Spontaneous miscarriage – first year* |  |  |  |  |
| Proportion of women suffering with moderate anxiety / depression | 0.15 (0.035) | Beta | α = 15.46, β = 87.62 – α‡ | Farren et al. 2018 ^34^ |
| Utility decrement with moderate anxiety / depression | 0.355 (0.033) | Beta | α = 74.29, β = 209.26 – α‡ | Kind et al. 1999^30^, Sobocki P et al. 2007^33^ |

Abbreviations: SE standard error, PTSD post-traumatic stress disorder.

*Remaining women (proportion=0.650) were assumed to be suffering with milder psychological symptoms and assigned utility decrement of 0.250.

†Numbers inferred using data from publication.

‡Estimated from the mean and standard error using the methods of moments approach.

§Proportion reduced by constant amount year on year until reaches zero at the end of year five.

## Second trimester termination maternal Markov model

Table S17 shows the proportion of women expected to suffer from post-traumatic stress (PTS) / psychiatric disorders at 12 months following a pregnancy termination.^42^ Utility for these women was decremented using a published utility score for PTS also shown in Table S17.^79^ We assumed that over five years maternal utility levels would recover at a constant annual rate.^41^ We also included an additional decrement following a second trimester termination, as when compared with a first trimester termination studies have shown trends towards increased levels of maternal grief, feelings of doubt and PTS.^41, 42^ In the absence of informative data, we implemented a small decrement, using a uniform distribution with minimum and maximum values of 0 and 0.01 respectively. This was maintained over the 20-year time horizon.

## Second trimester fetal loss with genetic testing maternal Markov model

We identified no studies assessing the longer-term psychological impact for mothers suffering a fetal loss following genetic testing. We thus maintained the utility decrements assigned to women in the decision tree model suffering such a loss in the second trimester (a duration of 20 weeks), for the first 32 weeks in the Markov model. Beyond this, we applied the same utility decrement assumptions made for women undergoing a second trimester termination (see above).

## Spontaneous miscarriage maternal Markov model

Informed by the published literature on the duration of negative psychological consequences following a miscarriage, we assumed that half of the 30% of women suffering moderate anxiety and depression in the 24 weeks following a miscarriage (within the decision tree), would still have moderate anxiety and depression for the first 28 weeks in the Markov model.^34^ For these women we again assumed a utility decrement for moderate depression of 0.355 (SE=0.033). Beyond 28 weeks and for the remaining 20-year time horizon of the model, we assumed no further utility decrement.

## First trimester termination maternal Markov model

For women who had undergone a first trimester termination, utility was adjusted in the same way as for women undergoing a second trimester termination, less the additional utility decrement assigned following termination at a more advanced gestational age.

## First trimester fetal loss with genetic testing maternal Markov model

For women suffering a first trimester fetal loss after genetic testing, utility was adjusted in the same way as for women suffering the same event in the second trimester, less the additional utility decrement assigned following the loss of a baby at a more advanced gestational age.

# Costs for the decision tree

Table S18 shows the unit costs associated with each of the events women experience as they move along the pathways within the decision tree model.

**Table S18 Unit Costs (2019/20 UK£) used in the decision tree part of the model.**

| **Event** | **Cost estimate (pounds)** | **Source** |
| --- | --- | --- |
| Ante-natal routine US scan | £125 | National Schedule of NHS Costs - Year 2019-20.^80^ Outpatients. Currency Code NZ21Z, Service Code 501 (obstetrics). |
| Fetal medicine US scan | £136 | National Schedule of NHS Costs - Year 2019-20.^80^ Outpatients. Currency Code NZ22Z, Service Code 501 (obstetrics). |
| Invasive genetic diagnostic test | £364 | National Schedule of NHS Costs - Year 2019-20.^80^ Outpatients. Currency Code NZ72Z, Service Code 501 (obstetrics). |
| Ante-natal echocardiogram | £144 | National Schedule of NHS Costs - Year 2019-20.^80^ Outpatients. Currency Code EC21Z, Service Code 501 (obstetrics). |
| First trimester termination | £1617 | National Schedule of NHS Costs - Year 2019-20.^80^ Total activity. Weighted average of Currency Codes MA51Z (Surgical abortion 14-20 weeks gestation) and MA54Z (Medical abortion 14-20 weeks gestation). |
| Spontaneous miscarriage | £622 | National Schedule of NHS Costs - Year 2019-20.^80^ Total activity. Currency Code MB08B. |
| First trimester iatrogenic fetal loss with genetic testing | £1311 | National Schedule of NHS Costs - Year 2019-20.^80^ Total activity. Currency Code MA54Z (Medical abortion or miscarriage care 14-20 weeks gestation). |
| Second trimester termination | £2553 | National Schedule of NHS Costs - Year 2019-20.^80^ Total activity. Weighted average of Currency Codes MA50Z (Surgical abortion over 20 weeks gestation) and MA53Z (Medical abortion over 20 weeks gestation). |
| Spontaneous stillbirth | £3681 | National Schedule of NHS Costs - Year 2019-20.^80^ Total activity. Weighted average across all delivery Currency Codes |
| Second trimester iatrogenic fetal loss with genetic testing | £2635 | National Schedule of NHS Costs - Year 2019-20.^80^ Total activity. Currency Code MA53Z (Medical abortion or miscarriage care over 20 weeks gestation). |
| Delivery | £3681 | National Schedule of NHS Costs - Year 2019-20.^80^ Total activity. Weighted average across all delivery Currency Codes |
| Post-stillbirth* | £1012 | Campbell et al. 2018 inflated using Curtis LA & Burns A. Unit Costs of Health and Social Care 2020^81, 82^ |

Abbreviation: US ultrasound.

*Includes cost of post-mortem and suite of investigations routinely performed following a stillbirth.

Women screening positive during the first trimester and leaving the routine screening pathway, were assumed to receive a further fetal medicine scan prior to any second trimester screening. In addition, and for major cardiac anomalies, exomphalos, gastroschisis, encephalocele, and LUTO, we costed a first trimester fetal echocardiogram. For screen positive women not suffering a miscarriage, during the second trimester, a fetal medicine screen was costed *in lieu* of a routine ultrasound scan. Women who then went on to have a live birth were assumed to have been screened by fetal medicine a further twice before their baby arrived. For screen positive women who suffered a stillbirth, only one further fetal medicine scan was costed. For all stillbirths in the model, the delivery of the baby was included as part of the costing process, as were the subsequent investigations / post-mortem.

When a pregnancy ended without a live birth, healthcare costs were included for the management of negative psychological consequences women may suffer immediately following the loss of their baby, up until the end of the decision tree time horizon (longer-term implications were captured in the maternal Markov models). Table S19 shows for each pregnancy loss event, the time point at which events were assumed to occur, and the proportions of women estimated to have psychological consequences significant enough to require medical intervention.

Based upon UK Psychiatric Morbidity Survey data, we assumed that only 64.5% (n=49/76) of women with negative psychological consequences would be in contact with health care services.^83^ This probability (implemented with a beta distribution) was applied to the proportions in Table S19, to determine the proportion of women receiving treatment. The expected annual cost of treatment for depression (including inpatient care, GP consultations, community mental health services, medication and residential care) was estimated to be £2,440 (after inflation to 2019/2020 prices)^83^. Dividing this cost by 52 gave a weekly cost estimate which was then assigned to women receiving such treatment from the point their pregnancy ended until the end of the decision tree time horizon (durations shown in the final column of Table S19).

**Table S19 - Proportions of women with clinically significant negative psychological symptoms immediately following a pregnancy loss event**

| **Pregnancy loss event** | **Assumed weeks’ gestation at time of loss** | **Mean proportion (SE) of women with clinically significant psychological symptoms following loss** | **Distribution and parameters** | **Source** | **Remaining time in decision tree following loss** |
| --- | --- | --- | --- | --- | --- |
| Spontaneous  stillbirth | 30 weeks* | 0.581 (0.0226) | Beta, α=275, β=473-α | Inferred using Redshaw et al. 2014^35^ and Heazell et al. 2016^36^ | 10 weeks |
| Second trimester termination / fetal loss with genetic testing | 20 weeks | First 6 weeks 0.875 (0.0802)‡ | Beta, α=14, β=16-α | Davies et al. 2005^42^ | 20 weeks |
|  |  | Post 6 weeks 0.571 (0.1278)‡ | Beta, α=8, β=14-α | Davies et al. 2005^42^ |  |
| Spontaneous miscarriage | 16 weeks† | 0.300 (0.035) | Beta, α=51.13, β=170.43-α§ | Farren et al. 2018^34^ /  Author Assumption | 24 weeks |
| First trimester termination / fetal loss with genetic testing | 12 weeks | First 6 weeks 0.429 (0.1278)‡ | Beta, α=6, β=14-α | Davies et al. 2005^42^ | 28 weeks |
|  |  | Post 6 weeks 0.417 (0.1367)‡ | Beta, α=5, β=12-α | Davies et al. 2005^42^ |  |

Abbreviation: SE standard error.

*Midpoint between 20-week anomaly scan and end of tree time horizon at 40 weeks

†Midpoint between first and second trimester scans

‡Proportion of women in Davies at el. with a cut off score >18 (indicating psychiatric morbidity) on the Impact of Event Scale (IES) following first and second trimester terminations.

§Estimated from mean and standard error using methods of moments approach.

# Costs for the maternal Markov models

## Live birth maternal Markov models

As with the modelling of utility, we assumed the impact upon a woman’s mental health and thus her need for treatment would depend upon whether her baby had been born with an anomaly, and if so, the implications of that anomaly for the child’s prognosis and survival.

Following the loss of a child during a model cycle (determined using the infant annual mortality rates shown in Table S15) we used the published literature to estimate the proportions of women in years one and two with psychological symptoms clinically significant enough to warrant medical intervention (shown in Table S20).^84^ Given the uncertainty around the duration for which symptoms persist, we used the same parameter representing uncertainty around the duration (in years) required for a mother’s quality of life to recover to underlying norm values following the death of her infant (see Table S16). For each value sampled from this distribution, we assumed a constant annual rate of reduction in the proportion of women affected by at two years.

When costing healthcare provided for significant maternal psychological symptoms following the death of an infant, we used the same healthcare engagement probability and treatment cost as used within the decision tree (see Table S20).

**Table S20 Parameters used to estimate annual costs in the live birth Markov models for each anomaly and for a live birth without an anomaly.**

| **Parameter** | **Mean (SE)** | **Distribution type** | **Parameters** | **Source** |
| --- | --- | --- | --- | --- |
| *Proportion of women who suffer the loss of their infant and who develop levels of psychological symptoms significant enough to warrant healthcare intervention* | | | | |
| Probability of significant maternal psychological symptoms in year 1 after the death of an infant | 0.594 (0.042) | Beta | α=82, β=138-α | Meert et al. 2011^84^ |
| Probability of significant maternal psychological symptoms in year 2 after the death of an infant | 0.384 (0.041) | Beta | α=53, β=138-α | Meert et al. 2011^84^ |
| Years for a mother’s significant psychological symptoms to diminish following the death of an infant | 12.500 (4.317) | Uniform | Min = 5 years  Max = 20 years | Author assumption |
| *Probability of symptom diagnosis and annual cost of treatment* | | | | |
| Probability of psychological symptoms diagnosed and treated | 0.645 (0.055) | -- | α=49, β=76-α | McCrone et al.^83^ |
| Annual cost of treatment for significant psychological symptoms | 2440.17 | -- | -- | McCrone et al.^83^  Jones and Burns 2021^82^ |
| *Proportion of women raising an infant with an anomaly and who develop levels of psychological symptoms significant enough to warrant healthcare intervention* | | | | |
| Annual probability of significant psychological symptoms in mother of a child with a genetic anomaly or a neurodevelopmental disability. Years 1-20. | 0.267 (0.079) | Beta | α=8, β=30-α | Swanepoel et al. 2018^68^ |
| Probability of significant psychological symptoms in mother of a child with a structural anomaly only. Year 1* | 0.301 (0.053) | Beta | α=22, β=73-α | Solberg et al. 2011^72^ |
| Proportion of mothers of a child with LUTO suffering significant psychological symptoms from Year 4 onwards | 0.056*model cycle number | -- | -- | Biard et al. 2005^62^  Berte et al. 2018^63^ |

Abbreviation: SE standard error

*The probability is reduced by a constant amount in years 2 and 3, and by year 4 takes on a value of 0.

For women having a live birth, those with babies affected by acrania and alobar holoprosencephaly, were assumed to suffer the loss of their infant soon after birth. For these women costs were modelled as described above for mothers suffering the loss of their child.

Mothers not losing their infant during a model cycle may still suffer distress as a result of the psychological, practical, and economic challenges associated with raising a child with a major congenital anomaly.^66-68^ As for utilities, such determinations were made by anomaly type (described below). In all maternal Markov models, for mothers predicted to have clinically significant negative psychological symptoms each year, we again assumed that a proportion (64.5%) would seek help and receive treatment at an expected annual cost of £2,440 (Table S20).

For mothers of babies surviving with a non-lethal structural anomaly co-existing with a genetic anomaly (a major cardiac anomaly, an omphalocele, an encephalocele, or a LUTO), we again assumed that the genetic anomaly would exert the greater impact each year. Based upon published data from a study of mother of infants with Down’s syndrome, we estimated that 26.7% of these women would likely suffer with clinically significant psychological symptoms each year (see Table S20).^68, 85^ Given the permanence of the condition, this proportion remained fixed during each model cycle.

As discussed for utilities, the five non-lethal anomalies within the protocol (a major cardiac anomaly, an omphalocele, an encephalocele, a LUTO and gastroschisis) can occur without a genetic anomaly. They are usually managed surgically following birth and this can also place a considerable burden upon mothers.^67, 69^ Using data from a study of mothers of infants born with a severe cardiac anomaly, we modelled that during the first year, 30% would suffer with clinically significant psychological symptoms (see Table S20).^72^ This proportion was also used for the other isolated ‘surgical’ anomalies in the absence of comparable published data. We assumed symptoms in mothers of infants with a major cardiac anomaly, an omphalocele, and gastroschisis, would diminish at a constant rate over three years. We assumed the same for isolated LUTO, but beyond four years we modelled that mothers whose infants develop renal failure (5.6% per year out to six years) would suffer clinically significant levels of depression (Table S20).^62, 63^

For mothers of the 40% of infants with an isolated encephalocele and no neurodevelopmental disability, costs were estimated as described for isolated major cardiac anomalies, omphalocele and gastroschisis. For mothers of infants with encephalocele and neurodevelopmental disability, we assumed the proportion with clinically significant psychological symptoms each year to be the same as for women raising an infant with a genetic anomaly (26.7%, Table S20)^68^

In the Markov model for a live birth with a genetic anomaly alone, we again assumed the proportion of women reaching clinically significant levels of depression each year to be 26.7%.^68^ Finally, and for women delivering babies unaffected by a congenital anomaly, costs were based upon the proportion estimated to suffer with post-natal depression each year (see the corresponding utility section above and Table S16).^65^

## Stillbirth maternal Markov model

As for utilities, 35% of women were assumed to experience significant psychological symptoms in the year following a stillbirth.^35, 36^ Symptoms diminished year on year at a constant rate so that by year five, no women required treatment.

## Second trimester termination maternal Markov model

We assumed the same annual proportions of women with significant psychological symptoms each year as used when estimating longer-term utilities following this outcome.

## Second trimester fetal loss with genetic testing maternal Markov model

The psychological impact for mothers of a second trimester fetal loss with genetic testing and the associated costs, were estimated, as for a second trimester termination.

## Spontaneous miscarriage maternal Markov model

Costs were estimated based on the assumption that 15% of women would still be suffering with moderate anxiety and depression for the first 28 weeks in the Markov model (see Markov model utility section above). Thereafter we assumed no further costs.

## First trimester termination maternal Markov model

Costs following first trimester terminations were estimated as per second trimester terminations.

## First trimester fetal loss with genetic testing maternal Markov model

Costs following first trimester fetal losses with genetic testing were estimated as per second trimester fetal losses with genetic testing.

# Implementing first trimester anomaly screening in the model

## First trimester screening outcome probabilities with the protocol

Table S21 below shows the anomaly-specific first trimester true positive detection probabilities considered achievable with a first trimester anomaly screening protocol. A comparison with current practice detection rates in Table S2 shows the likelihood of detection with the protocol to be higher for all eight anomalies.

**Table S21 First trimester screening outcomes achievable with protocol**

| **Anomaly type,**  **Protocol screening performance in first trimester** | **Mean (SE)*** | **Distribution type and parameters** | **Source** |
| --- | --- | --- | --- |
| Major cardiac anomaly |  |  |  |
| T1 TP – with protocol‡ | 0.3296 (0.0804) | Beta, α=10.94, β=33.18 – α† | Karim et al. 2022^2^ |
| T1 FP – sonographer | 0.00008 (0.0000178) | Beta, α=20.20, β=252,472.17 – α† | Karim et al. 2022^2^ / expert opinion |
| T1 FP - fetal medicine | 0.00004 (0.0000178) | Beta, α=5.05, β=126,240.64 – α† | Karim et al. 2022^2^ / expert opinion |
| Acrania |  |  |  |
| T1 TP – with protocol | 0.9796 (0.0080) | Beta, α=304.90, β=311.25 – α† | Karim et al. 2024^1^ |
| T1 FP – sonographer | 0.00001 (0.000005) | Beta, α=4.0, β=399,995 – α† | Karim et al. 2024^1^ / expert opinion |
| T1 FP - fetal medicine | 0.00000 | -- | Karim et al. 2024^1^ / expert opinion |
| Exomphalos |  |  |  |
| T1 TP – with protocol | 0.9568 (0.0130) | Beta, α=233.06, β=243.58 – α† | Karim et al. 2024^1^ |
| T1 FP – sonographer§ | 0.0018 (0.0000181) | Beta, α=9,872.00, β=5,484,446.97 – α† | Karim et al. 2024^1^ / expert opinion |
| T1 FP - fetal medicine§ | 0.0015 (0.0000181) | Beta, α=6,857.62, β=4,571,745.89 – α† | Karim et al. 2024^1^ / expert opinion |
| Gastroschisis |  |  |  |
| T1 TP – with protocol | 0.9595 (0.0177) | Beta, α=118.05, β=123.04 – α† | Karim et al. 2024^1^ |
| T1 FP – sonographer | 0.00002 (0.0000072) | Beta, α=7.72, β=385,793.75 – α† | Karim et al. 2024^1^ / expert opinion |
| T1 FP - fetal medicine | 0.000005 (0.0000072) | Beta, α=0.482, β=96,449.14 – α† | Karim et al. 2024^1^ / expert opinion |
| Alobar holoprosencephaly |  |  |  |
| T1 TP – with protocol | 0.9175 (0.0251) | Beta, α=109.32, β=119.15 – α† | Karim et al. 2024^1^ |
| T1 FP – sonographer | 0.00002 | Beta, α=6.25, β=312,492.75 – α† | Karim et al. 2024^1^ / expert opinion |
| T1 FP - fetal medicine | 0.000005 (0.000008) | Beta, α=0.391, β=78,123.61 – α† | Karim et al. 2024^1^ / expert opinion |
| Lower Urinary Tract Obstruction |  |  |  |
| T1 TP – with protocol | 0.6613 (0.0500) | Beta, α=58.59, β=88.59 – α† | Karim et al. 2024^1^ |
| T1 FP – sonographer§ | 0.0009 (0.000056) | Beta, α=285.06, β=286,730.51 – α† | Karim et al. 2024^1^ / expert opinion |
| T1 FP - fetal medicine§ | 0.0005 (0.000056) | Beta, α=79.68, β=159,358.06 – α† | Karim et al. 2024^1^ / expert opinion |
| Encephalocele |  |  |  |
| T1 TP – with protocol | 0.8990 (0.0373) | Beta, α=57.77, β=64.26 – α† | Karim et al. 2024^1^ |
| T1 FP – sonographer | 0.00001 (0.0000052) | Beta, α=3.70, β=369,817.79– α† | Karim et al. 2024^1^ / expert opinion |
| T1 FP - fetal medicine | 0.000001 (0.0000052) | Beta, α=0.037, β=36,981.21 – α† | Karim et al. 2024 ^1^/ expert opinion |
| Body Stalk Anomaly |  |  |  |
| T1 TP – with protocol | 0.9859 (0.0106) | Beta, α=120.99, β=122.72 – α† | Karim et al. 2024^1^ |
| T1 FP – sonographer | 0.00001 (0.0000065) | Beta, α=2.37, β=236,683.02 – α† | Karim et al. 2024^1^ / expert opinion |
| T1 FP - fetal medicine | 0.00000 | - | Karim et al. 2024^1^ / expert opinion |

Abbreviations: T1 first trimester, TP true positive, FP false positive, SE standard error

*Using systematic review data standard errors could only be estimated for the FP rate of screening overall (sonographer plus fetal medicine FPs) for each anomaly. These estimates were thus used to reflect uncertainty around both sonographer and fetal medicine FP rates in the model.

†Estimated from mean and standard error using methods of moments approach.

‡Based on 4 chamber view protocol (No doppler).

§Anomaly is present at the time of screening but spontaneously resolves as the pregnancy progresses and so is considered a T1 false positive finding. The difference between T1 sonographer and T1 fetal medicine FPs is accounted for by some anomalies resolving between being identified by a sonographer and being screened by a fetal medicine specialist.

It is acknowledged that for certain anomalies, a first trimester screening protocol may lead to an increase in sonographer false positive findings when compared with current practice. A comparison of Tables S2 and S21 shows such increases were modelled for major cardiac anomaly, alobar holoprosencephaly, and encephalocele. Finally, and guided by expert opinion, we assumed that the fetal medicine false positive rates in both arms of the model would be the same.

## Additional screening costs

Two additional costs associated with anomaly screening were included in the model. Firstly, we estimated the costs of sonographer training required to ensure proficiency in assessing parts of the fetal anatomy affected by the anomalies in the protocol. Guided by experts, we assumed five days of training (a mix of initial training plus later consolidation sessions) provided at an NHS Trust level by a local clinical lead. With an estimated 3,000 NHS sonographers, the majority of whom are employed at Band 7 at a cost of £65 per working hour (inclusive of salary, on-costs, overheads and indirect costs), total training costs are estimated at £7,312,500 (3,000 sonographers x 37.5 sonographer hours for training x £65 per hour).^82, 86, 87^ If provided by a Consultant in fetal medicine (£123 per working hour inclusive of salary, on-costs, overheads and indirect costs), then the estimated cost of delivering this training is £964,013 (209 NHS Trusts x 37.5 consultant hours for training x £123 per hour). Total training costs are estimated at £8,276,513.

Whilst training is fundamental, it is an ‘up-front’ cost, with the knowledge gained used when screening all subsequent women. To include this cost within the analysis therefore, we assumed that around 6,163,070 women (based upon 616,307 live births and stillbirths in England and Wales in 2020)^88^ would present for first trimester screening over the next 10 years. Dividing the total training cost by this figure produced a training cost per scan of £1.34.

The second cost component is the need for additional screening time to assess the fetal anatomy. Based upon expert opinion and findings from our nationwide site survey on current first trimester screening practices, we assumed an additional 10 minutes of screening time would be needed.^89^ The cost of this extra sonographer time at £10.80 per scan (£65/60 * 10) was added to the training cost per scan (£1.34) and entered into the screening protocol arm of the model. To reflect uncertainty around this estimate, we used a gamma distribution with mean £12.14 and standard error of £7. These moments produced a wide sampling distribution with cost estimates of £2.51 and £29.21 for the 2.5th and 97.5th percentiles respectively (equivalent to additional scanning times of 1 minute and 26 minutes respectively).

## Utility impact

The utility impact of first trimester anomaly screening was determined by the screening performance probabilities shown in Table S21, which altered the proportions of women moving along the screening outcome sub-trees and thus exposed to the screening and pregnancy outcomes and associated utility implications described in sub-sections 7.1 to 7.4 above. A small transitory utility increment (0.01 for 8 weeks) was also included in the model for all women accepting the invitation for first trimester anomaly screening and receiving a negative scan result.

# Deterministic Sensitivity Analyses

The various one-way deterministic sensitivity analyses conducted included:

1. An increase in the additional screening time required from 10 to 20 minutes
2. The assumption that maternal utility levels recovered to approximately population norm values, five years following the loss of a child
3. The assumption that maternal utility levels recovered to approximately population norm values, 20 years following the loss of a child
4. The assumption that the maternal utility impact of a termination is the same in the first and second trimesters (the additional utility decrement for second trimester termination is removed)
5. The utility increment reflecting the reassurance provided by a negative first trimester anomaly screen was set to 0 (utility increment is removed)
6. The utility increment reflecting the reassurance provided by a negative first trimester anomaly screen was set to 0.01
7. The utility increment reflecting the reassurance provided by a negative first trimester anomaly screen was set to 0.02

Table S22 below shows the results of these analyses.

**Table S22 Deterministic sensitivity analysis results for mean maternal healthcare cost, QALY differences and cost-effectiveness**

| Analysis Description | Mean cost difference (95% CI) | Mean QALY difference  (95% CI) | Incremental cost per QALY gained | Probability T1 anomaly screening is cost-effective at £20,000 per QALY |
| --- | --- | --- | --- | --- |
| Base-case results | £11  (£1 to £29) | 0.002065  (0.00056 to 0.00358) | £5,270 | 95.45% |
| 1. An increase in the additional screening time assumed to be required from 10 to 20 minutes | £22  (£10 to £38) | 0.002065  (0.00056 to 0.00358) | £10,514 | 83.70% |
| 2. The duration over which a woman’s utility recovers to underlying norm levels following the live birth and then loss of her infant is set to 20 years. | £11  (£1 to £28) | 0.002337  (0.00082 to 0.00389) | £4,580 | 97.88% |
| 3. The duration over which a woman’s utility recovers to underlying norm levels following the live birth and then loss of her infant is set to 5 years. | £11  (£1 to £29) | 0.001754  (0.00039 to 0.00312) | £6,326 | 91.04% |
| 4. The additional utility decrements assigned to women undergoing a second trimester termination are removed. | £11  (£1 to £29) | 0.001961  (0.00048 to 0.00347) | £5,552 | 93.86% |
| 5. The temporary reassurance utility increment assigned to women following a negative anomaly scan is fixed at 0 (i.e. no reassurance received). | £11  (£1 to £29) | 0.000674  (0.00020 to 0.00134) | £16,147 | 62.92% |
| 6. The temporary reassurance utility increment assigned to women following a negative anomaly scan is fixed at 0.01. | £11  (£1 to £29) | 0.002061  (0.00159 to 0.00273) | £5,281 | 99.59% |
| 7. The temporary reassurance utility increment assigned to women following a negative anomaly scan is fixed at 0.02. | £11  (£1 to £29) | 0.003448  (0.00298 to 0.00413) | £3,157 | 100.00% |

# Value of Information (VoI) Analysis

We followed the approach recommended by the ISPOR Value of Information Analysis Emerging Good Practices Task Force to undertake our VoI analysis.^90^

Results utilised the net-benefit statistic. Details of this metric have been reported elsewhere, but in brief, the incremental net monetary benefit (INMB) is estimated by first monetising the mean QALY gain from an intervention (here maternal QALYs).^91, 92^ This is done by multiplying the estimated mean QALY gain by a value reflecting society’s willingness to pay for a QALY (here we used £20,000). From this monetary gain, the additional costs of the intervention are subtracted and if the resulting figure is positive, the additional benefits are greater than the additional costs and the intervention is considered cost-effective. Figure S7 below shows the distribution of INMB calculated using the cost and QALY results from each of the 10,000 runs of the model.

**Figure S7 Incremental net monetary benefit comparing first trimester anomaly screening with usual practice.**

**
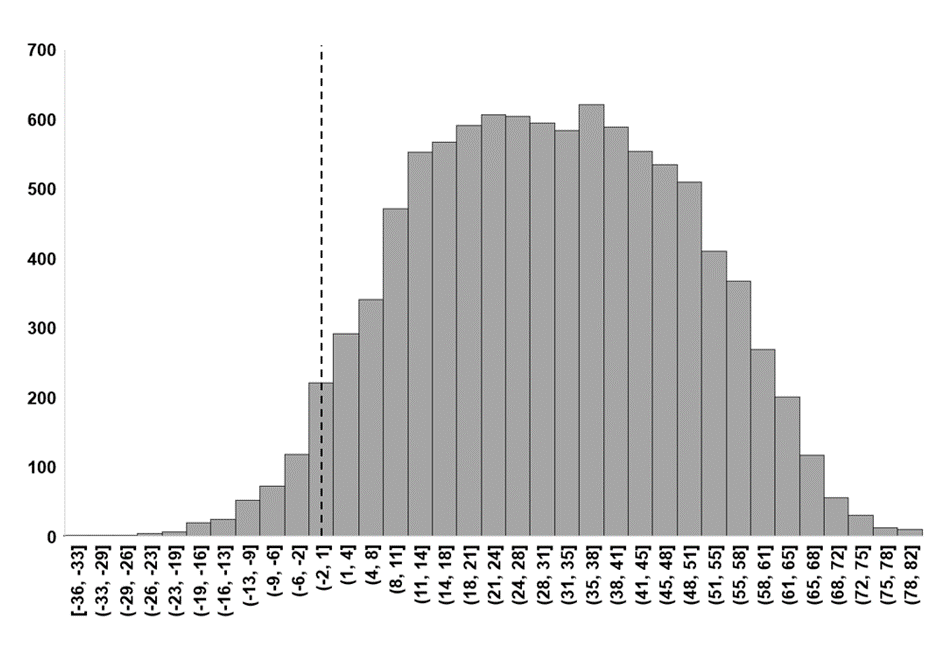
**

Positive values indicate first trimester anomaly screening is cost-effective.

The figure shows how the proportion of model simulations generating INMBs less than zero (to the left of the dashed vertical line) and thus suggesting first trimester structural anomaly screening would not be cost-effective, is low.

To determine whether conducting more research to further reduce uncertainty was potentially worthwhile, we first estimated the expected value of perfect information (EVPI) and the expected value of partial perfect information (EVPPI). For a full explanation of EVPI and EVPPI, see Wilson.^91^ We took a non-parametric approach to EVPI using the INMB results generated from the 10,000 runs of the model, directly. In simple terms, EVPI is the probability of being ‘wrong’ (the proportion of the distribution in Figure S7 falling to the left of the vertical line) multiplied by the average consequence of being wrong (estimated from the negative INMB values to the left of the vertical line in Figure S7). We estimated EVPI per person and for the entire population to benefit from screening (616,307 women per year) over 20 years (the duration for which the decision question around first trimester screening was assumed to remain relevant). Population EVPI values were compared with the expected costs of research to establish whether further research was potentially worthwhile.

We estimated EVPPI to identify whether research efforts should be concentrated on studies to remove uncertainty on a group of parameters. Parameters were grouped as shown in Table S23. EVPPI was estimated using the Sheffield Accelerated Value of Information (SAVI) tool that implements a non-parametric regression method using a generalized additive model (GAM) for groups of up to five parameters and Gaussian process regression for groups with five or more parameters.^93^ Population EVPPIs were compared with the expected costs of research for each group of parameters to determine whether research was potentially valuable.

**Table S23 Parameter groupings for the EVPPI analysis**

| **Group of parameters** | **Description** | **Number of parameters included** |
| --- | --- | --- |
| Anomaly characteristics | Anomaly prevalences, and co-existence with genetic anomalies at first and second trimester screening points | 17 |
| Additional screening cost | Additional cost per anomaly scan, (staff training and extra scanning time) | 1 |
| Maternal psychological symptoms | Probability of maternal psychological symptoms with each of the modelled pregnancy outcomes | 19 |
| Pregnancy outcomes | Anomaly specific probabilities for each pregnancy outcome e.g. first and second trimester terminations, miscarriages, stillbirths etc. | 57 |
| Screening performance | First and second trimester anomaly specific screening performance probabilities e.g. true positives, and sonographer and fetal medicine false positives. | 58 |
| Screening and testing acceptance | Probabilities of accepting first trimester anomaly screening, and of accepting genetic diagnostic testing. | 2 |
| Maternal utility (screening) | Utility increment for reassurance following a negative anomaly scan and utility decrement after a false positive scan result is corrected. | 2 |
| Maternal utility (all other) | Underlying maternal utility levels and utility decrements for all other screening and pregnancy outcomes | 19 |

# Infant Markov models

A further set of Markov models was developed to simulate the long-term prognosis, and expected healthcare costs and QALYs of live born infants in each arm of the decision tree. In total, 13 separate models were constructed covering live births with each type of anomaly (with and without genetic involvement genetically associated anomalies) (n=11), the live birth of an unaffected child (n=1), and the live birth of a child with a genetic anomaly alone (n=1). These models were structured as per the maternal Markov models (see Figure S6) with all infants starting in the alive health state. Daily cycle lengths were used for lethal anomalies (acrania and alobar holoprosencephaly) resulting in death in the days or weeks following birth, and annual cycles were used for unaffected babies and those born with anomalies that can be managed surgically and have greater life expectancy. The model time horizon was 20 years.

Transitions from the alive to the deceased health state within each infant model were based upon the same anomaly specific infant annual mortality risks used in the maternal Markov models (Table S15). Daily mortality risks were obtained for the models for acrania and alobar holoprosencephaly.^55, 58^ Full details of the studies and data informing the costs and utilities feeding into these models are available from the authors upon request. Briefly, underlying utility for each cycle in the alive health state of a model, was informed by Health Utilities Index 3 (HUI3) reference scores in a population of Canadian children.^94^ Within each model, underlying utility levels were then decremented for quality of life impact of the specific anomaly being modelled. Decrements were taken from a UK study estimating utilities (using the HUI3) for 2,236 children with a range of childhood conditions and were applied for varying durations as determined by the prognosis of the anomaly being modelled.^95^ Costs included in each model were informed by the published literature and captured the costs of initial and subsequent corrective surgeries (when applicable), ongoing healthcare needs, and end of life care (for lethal anomalies).

Each model was run 10,000 times (each time sampling a set of parameter estimates from the distributions entered) and the expected infant healthcare costs and QALYs associated with each type of live birth were extracted. These outputs are shown in Table S24. These costs and QALYs were then used as model input parameters and were attached as secondary pay-offs (using gamma distributions) to their corresponding live birth endpoints in the decision tree model. Decision tree pathways culminating in the loss of a baby were assigned zero infant healthcare costs and QALYs.

**Table S24** **Expected 20-year mean discounted infant healthcare costs and QALYs per live birth by anomaly type, and for a healthy infant (used as payoffs within the decision tree)**

| **Anomaly Type** | **Mean (SE) 20-year Cost** | **Mean (SE) 20-year QALY** |
| --- | --- | --- |
| Major cardiac anomaly + genetic anomaly | £119,874 (£5,299) | 4.173 (0.767) |
| Major cardiac anomaly – genetic anomaly | £77,933 (£5,270) | 9.643 (0.231) |
| Acrania | £3,575 (£245) | 0.000 (0.000) |
| Omphalocele/Exomphalos + genetic anomaly | £59,635 (£6,323) | 1.538 (0.581) |
| Omphalocele/Exomphalos – genetic anomaly | £57,335 (£539) | 11.784 (0.345) |
| Gastroschisis | £90,381 (£268) | 12.285 (0.240) |
| Alobar holoprosencephaly | £15,368 (£-) | 0.000 (0.000) |
| LUTO + genetic anomaly | £90,404 (£7,287) | 3.249 (0.766) |
| LUTO – genetic anomaly | £57,008 (£6,876) | 9.894 (0.585) |
| Encephalocele + genetic anomaly | £67,346 (£3,591) | 2.778 (0.573) |
| Encephalocele – genetic anomaly | £57,117 (£3,379) | 4.858 (0.623) |
| Body Stalk Anomaly | NA | NA |
| Genetic anomaly alone | £57,195 (£191) | 4.168 (0.746) |
| Infant without anomaly | £25,159 (£-) | 13.241 (0.156) |

Abbreviations: LUTO lower urinary tract obstruction, SE standard error. NA not applicable as the model simulated no live births with body stalk anomaly.

# Members of the Assessing Clinical and Cost Effectiveness of Prenatal first-Trimester anomaly Screening (ACCEPTS) group

**Clinical and study design team**: Aris T Papageorghiou (PI, University of Oxford, UK), Zarko Alfirevic (Liverpool Women’s NHS Foundation Trust, UK), Trish Chudleigh (Cambridge University Hospitals NHS Foundation Trust, UK), Hilary Goodman (Hampshire Hospital NHS Foundation Trust, UK), Christos Ioannou (Oxford University Hospitals NHS Trust, UK), Heather Longworth (Liverpool Women’s NHS Foundation Trust, UK), Jehan N Karim (University of Oxford, UK), Kypros H Nicolaides (Fetal Medicine Research Institute, King’s College Hospital, UK), Pranav Pandya (University College London Hospitals NHS Foundation Trust, UK), Gordon Smith (University of Cambridge, UK), Basky Thilaganathan (St George's University Hospitals NHS Foundation Trust, UK) and Jim Thornton (University of Nottingham, UK).

**Health economics team**: Oliver Rivero-Arias (Lead, University of Oxford, UK), Helen Campbell (University of Oxford, UK), Ed Juszczak (University of Oxford, UK), Louise Linsell (University of Oxford, UK) and Ed Wilson (University of Exeter, UK).

**Qualitative research**: Lisa Hinton (University of Cambridge, UK)

**Patient Voice**: Jane Fisher (Lead, Antenatal Results and Choices, UK), Elizabeth Duff (National Childbirth Trust, UK), Anne Rhodes (Tiny Tickers, UK), Gil Yaz (SHINE UK).

# References

1. Karim J, Di Mascio D, Roberts N, Papageorghiou AT. Detection of non-cardiac fetal abnormalities by ultrasound at 11-14 weeks: systematic review and meta-analysis. Ultrasound in obstetrics & gynecology : the official journal of the International Society of Ultrasound in Obstetrics and Gynecology. 2024;doi: 10.1002/uog.27649.

2. Karim JN, Bradburn E, Roberts N, Papageorghiou AT. First-trimester ultrasound detection of fetal heart anomalies: systematic review and meta-analysis. Ultrasound in obstetrics & gynecology : the official journal of the International Society of Ultrasound in Obstetrics and Gynecology. 2022;59(1):11-25.

3. Karim J, Gordijn S, Papageorghiou AT. OC18.08: Developing a first trimester anomaly screening protocol for the UK: a consensus procedure. Ultrasound in Obstetrics & Gynecology. 2021;58(S1):54-5.

4. European network of population based registries for the epidemiological surveillance of congenital anomalies EUROCAT. Prevalence charts and tables for congenital fetal anomalies (UK) 2011-2018 [Internet]. European Commission - European Platform on Rare Disease Registration. 2021 [cited 3rd January 2022]. Available from: <https://eu-rd-platform.jrc.ec.europa.eu/eurocat/eurocat-data/prevalence_en>.

5. Public Health England. National Congenital Anomaly and Rare Disease Registration Service: Congenital anomaly statistics 2019. London: Public Health England; 2021 September 2021.

6. Spencer K, Spencer CE, Power M, Dawson C, Nicolaides KH. Screening for chromosomal abnormalities in the first trimester using ultrasound and maternal serum biochemistry in a one-stop clinic: a review of three years prospective experience. BJOG : an international journal of obstetrics and gynaecology. 2003;110(3):281-6.

7. Malin G, Tonks AM, Morris RK, Gardosi J, Kilby MD. Congenital lower urinary tract obstruction: a population-based epidemiological study. BJOG : an international journal of obstetrics and gynaecology. 2012;119(12):1455-64.

8. European network of population based registries for the epidemiological surveillance of congenital anomalies EUROCAT. Prenatal Detection Rates Charts and Tables 2015-2019 [Internet]. European Platform on Rare Disease Registration. 2022 [cited 3rd January 2022]. Available from: <https://eu-rd-platform.jrc.ec.europa.eu/eurocat/eurocat-data/prenatal-screening-and-diagnosis_en>.

9. Syngelaki A, Guerra L, Ceccacci I, Efeturk T, Nicolaides KH. Impact of holoprosencephaly, exomphalos, megacystis and increased nuchal translucency on first-trimester screening for chromosomal abnormalities. Ultrasound in Obstetrics & Gynecology. 2017;50(1):45-8.

10. Liao AW, Sebire NJ, Geerts L, Cicero S, Nicolaides KH. Megacystis at 10-14 weeks of gestation: chromosomal defects and outcome according to bladder length. Ultrasound in Obstetrics & Gynecology. 2003;21(4):338-41.

11. Public Health England. National Congenital Anomaly and Rare Disease Registration Service: Congenital anomaly statistics 2018. London: Public Health England; 2020 27 July 2021.

12. Salomon LJ, Sotiriadis A, Wulff CB, Odibo A, Akolekar R. Risk of miscarriage following amniocentesis or chorionic villus sampling: systematic review of literature and updated meta-analysis. Ultrasound in obstetrics & gynecology : the official journal of the International Society of Ultrasound in Obstetrics and Gynecology. 2019;54(4):442-51.

13. Hartge DR, Niemeyer L, Axt-Fliedner R, Krapp M, Gembruch U, Germer U, et al. Prenatal detection and postnatal management of double outlet right ventricle (DORV) in 21 singleton pregnancies. Journal of Maternal-Fetal & Neonatal Medicine. 2012;25(1):58-63.

14. Eleftheriades M, Tsapakis E, Sotiriadis A, Manolakos E, Hassiakos D, Botsis D. Detection of congenital heart defects throughout pregnancy; impact of first trimester ultrasound screening for cardiac abnormalities. Journal of Maternal-Fetal & Neonatal Medicine. 2012;25(12):2546-50.

15. Orlandi E, Rossi C, Perino A, Musicò G, Orlandi F. Simplified first‐trimester fetal cardiac screening (four chamber view and ventricular outflow tracts) in a low‐risk population. Prenatal diagnosis. 2014;34(6):558-63.

16. Sainz JA, Gutierrez L, García-Mejido J, Ramos Z, Bonomi MJ, Fernández-Palacín A, et al. Early fetal morphological evaluation (11–13+ 6 weeks) accomplished exclusively by transabdominal imaging and following routine midtrimester fetal ultrasound scan recommendations. Since when can it be performed? The Journal of Maternal-Fetal & Neonatal Medicine. 2018;33(7):1140-50.

17. Minnella GP, Crupano FM, Syngelaki A, Zidere V, Akolekar R, Nicolaides KH. Diagnosis of major heart defects by routine first-trimester ultrasound examination: association with increased nuchal translucency, tricuspid regurgitation and abnormal flow in ductus venosus. Ultrasound in obstetrics & gynecology : the official journal of the International Society of Ultrasound in Obstetrics and Gynecology. 2020;55(5):637-44.

18. Syngelaki A, Chelemen T, Dagklis T, Allan L, Nicolaides KH. Challenges in the diagnosis of fetal non-chromosomal abnormalities at 11-13 weeks. Prenat Diagn. 2011;31(1):90-102.

19. Grande M, Arigita M, Borobio V, Jimenez JM, Fernandez S, Borrell A. First-trimester detection of structural abnormalities and the role of aneuploidy markers. Ultrasound in Obstetrics & Gynecology. 2012;39(2):157-63.

20. Liao Y, Wen H, Ouyang S, Yuan Y, Bi J, Guan Y, et al. Routine first-trimester ultrasound screening using a standardized anatomical protocol. American Journal of Obstetrics and Gynecology. 2021;224(4):396. e1-. e15.

21. Kagan KO, Staboulidou I, Syngelaki A, Cruz J, Nicolaides KH. The 11-13-week scan: diagnosis and outcome of holoprosencephaly, exomphalos and megacystis. Ultrasound in obstetrics & gynecology : the official journal of the International Society of Ultrasound in Obstetrics and Gynecology. 2010;36(1):10-4.

22. Murphy A, Platt LD. First-trimester diagnosis of body stalk anomaly using 2- and 3-dimensional sonography. Journal of Ultrasound in Medicine. 2011;30(12):1739-43.

23. Daskalakis G, Sebire NJ, Jurkovic D, Snijders RJ, Nicolaides KH. Body stalk anomaly at 10-14 weeks of gestation. Ultrasound in Obstetrics & Gynecology. 1997;10(6):416-8.

24. Morris JK, Savva GM. The risk of fetal loss following a prenatal diagnosis of trisomy 13 or trisomy 18. American Journal of Medical Genetics. 2008;Part A. 146(7):827-32.

25. Garne E, Stoll C, Clementi M. Evaluation of prenatal diagnosis of congenital heart diseases by ultrasound: experience from 20 European registries. Ultrasound in Obstetrics and Gynecology: The Official Journal of the International Society of Ultrasound in Obstetrics and Gynecology. 2001;17(5):386-91.

26. Garne E, Loane M, Dolk H, De Vigan C, Scarano G, Tucker D, et al. Prenatal diagnosis of severe structural congenital malformations in Europe. Ultrasound in obstetrics & gynecology : the official journal of the International Society of Ultrasound in Obstetrics and Gynecology. 2005;25(1):6-11.

27. Draper ES GI, Smith LK, Kurinczuk JJ, Smith PW, Boby T, Fenton AC, Manktelow BN, on behalf of the MBRRACE-UK Collaboration. MBRRACE-UK Perinatal Mortality Surveillance Report, UK Perinatal Deaths for Births from January to December 2017. Leicester; 2019.

28. Office for National Statistics. National Life Tables: England and Wales 2017-2019 [Internet]. 2020 [cited 13 June 2021]. Available from: <https://www.ons.gov.uk/peoplepopulationandcommunity/birthsdeathsandmarriages/lifeexpectancies/datasets/nationallifetablesenglandandwalesreferencetables/current>

29. Fuller AE, Horváth-Puhó E, Ray JG, Ehrenstein V, Sørensen HT, Cohen E. Mortality Among Parents of Children With Major Congenital Anomalies. Pediatrics. 2021;147(5).

30. Kind P. HG, Macran S. UK population norms for the EQ-5D. Discussion Paper 172. York; 1999.

31. Kuppermann M, Norton ME, Thao K, O'Leary A, Nseyo O, Cortez A, et al. Preferences regarding contemporary prenatal genetic tests among women desiring testing: implications for optimal testing strategies. Prenat Diagn. 2016;36(5):469-75.

32. Kaasen A, Helbig A, Malt UF, Næs T, Skari H, Haugen G. Maternal psychological responses during pregnancy after ultrasonographic detection of structural fetal anomalies: A prospective longitudinal observational study. PLoS One. 2017;12(3):e0174412.

33. Sobocki P, Ekman M, Agren H, Krakau I, Runeson B, Mårtensson B, et al. Health-related quality of life measured with EQ-5D in patients treated for depression in primary care. Value in health : the journal of the International Society for Pharmacoeconomics and Outcomes Research. 2007;10(2):153-60.

34. Farren J, Mitchell-Jones N, Verbakel JY, Timmerman D, Jalmbrant M, Bourne T. The psychological impact of early pregnancy loss. Human reproduction update. 2018;24(6):731-49.

35. Redshaw M RR, Henderson J. . Listening to Parents After Stillbirth or the Death of Their Baby After Birth. Oxford: National Perinatal Epidemiology Unit, University of Oxford; 2014.

36. Heazell AEP, Siassakos D, Blencowe H, Burden C, Bhutta ZA, Cacciatore J, et al. Stillbirths: economic and psychosocial consequences. Lancet. 2016;387(10018):604-16.

37. Thomas GM, Roberts J, Griffiths FE. Ultrasound as a technology of reassurance? How pregnant women and health care professionals articulate ultrasound reassurance and its limitations. Sociology of health & illness. 2017;39(6):893-907.

38. Garcia J, Bricker L, Henderson J, Martin MA, Mugford M, Nielson J, et al. Women's views of pregnancy ultrasound: a systematic review. Birth (Berkeley, Calif). 2002;29(4):225-50.

39. Clement S WJ, Sikorski J. Women’s experiences of antenatal ultrasound scans. In: S. C, editor. Psychological Perspectives on Pregnancy and Childbirth. Edinburgh: Churchill Livingstone; 1998. p. 7-24.

40. Karim JC, R.; Davidson, LM.; Maiz, N.; Fisher, J.; Rivero-Arias, O.; Hinton, L.; Papageorghiou, AT. Acceptability of the first trimester anomaly scan amongst parents in the UK. . BJOG: Int J Obstet Gy. 2021;128:271-81.

41. Korenromp MJ, Christiaens GCML, van den Bout J, Mulder EJH, Hunfeld JAM, Bilardo CM, et al. Long-term psychological consequences of pregnancy termination for fetal abnormality: a cross-sectional study. Prenatal Diagnosis. 2005;25(3):253-60.

42. Davies V, Gledhill J, McFadyen A, Whitlow B, Economides D. Psychological outcome in women undergoing termination of pregnancy for ultrasound-detected fetal anomaly in the first and second trimesters: a pilot study. Ultrasound in obstetrics & gynecology : the official journal of the International Society of Ultrasound in Obstetrics and Gynecology. 2005;25(4):389-92.

43. Heckerling PS, Verp MS. Amniocentesis or chorionic villus sampling for prenatal genetic testing: a decision analysis. Journal of clinical epidemiology. 1991;44(7):657-70.

44. Heckerling PS, Verp MS. A cost-effectiveness analysis of amniocentesis and chorionic villus sampling for prenatal genetic testing. Medical care. 1994;32(8):863-80.

45. Heckerling PS, Verp MS, Hadro TA. Preferences of pregnant women for amniocentesis or chorionic villus sampling for prenatal testing: comparison of patients' choices and those of a decision-analytic model. Journal of clinical epidemiology. 1994;47(11):1215-28.

46. Deverill M, Robson S. Women's preferences in screening for Down syndrome. Prenat Diagn. 2006;26(9):837-41.

47. Marteau TM, Cook R, Kidd J, Michie S, Johnston M, Slack J, et al. The psychological effects of false-positive results in prenatal screening for fetal abnormality: a prospective study. Prenat Diagn. 1992;12(3):205-14.

48. Baillie C. SJ, Hewison J., Mason G. Ultrasound screening for chromosomal abnormality: Women’s reactions to false positive results. British Journal of Health Psychology. 2000;5:377-94.

49. Chung EH, Lim SL, Havrilesky LJ, Steiner AZ, Dotters-Katz SK. Cost-effectiveness of prenatal screening methods for congenital heart defects in pregnancies conceived by in-vitro fertilization. Ultrasound in obstetrics & gynecology : the official journal of the International Society of Ultrasound in Obstetrics and Gynecology. 2021;57(6):979-86.

50. Odibo AO, Coassolo KM, Stamilio DM, Ural SH, Macones GA. Should all pregnant diabetic women undergo a fetal echocardiography? A cost-effectiveness analysis comparing four screening strategies. Prenat Diagn. 2006;26(1):39-44.

51. Vandenbussche FP, De Jong-Potjer LC, Stiggelbout AM, Le Cessie S, Keirse MJ. Differences in the valuation of birth outcomes among pregnant women, mothers, and obstetricians. Birth (Berkeley, Calif). 1999;26(3):178-83.

52. Christiansen DM. Posttraumatic stress disorder in parents following infant death: A systematic review. Clinical psychology review. 2017;51:60-74.

53. Morris S, Fletcher K, Goldstein R. The Grief of Parents After the Death of a Young Child. Journal of clinical psychology in medical settings. 2019;26(3):321-38.

54. Tennant PW, Pearce MS, Bythell M, Rankin J. 20-year survival of children born with congenital anomalies: a population-based study. Lancet. 2010;375(9715):649-56.

55. Baird PA, Sadovnick AD. Survival in infants with anencephaly. Clinical pediatrics. 1984;23(5):268-71.

56. Springett A, Draper ES, Rankin J, Rounding C, Tucker D, Stoianova S, et al. Birth prevalence and survival of exomphalos in england and wales: 2005 to 2011. Birth defects research Part A, Clinical and molecular teratology. 2014;100(9):721-5.

57. Bradnock TJ, Marven S, Owen A, Johnson P, Kurinczuk JJ, Spark P, et al. Gastroschisis: one year outcomes from national cohort study. BMJ (Clinical research ed). 2011;343:d6749.

58. Bullen PJ, Rankin JM, Robson SC. Investigation of the epidemiology and prenatal diagnosis of holoprosencephaly in the North of England. Am J Obstet Gynecol. 2001;184(6):1256-62.

59. Song J, Floyd FJ, Seltzer MM, Greenberg JS, Hong J. Long-term Effects of Child Death on Parents' Health Related Quality of Life: A Dyadic Analysis. Family relations. 2010;59(3):269-82.

60. D’Souza R. Exploring Methodologic Challenges in the Conduct of Patient Preference Studies in Obstetrics. A thesis submitted in conformity with the requirements for the degree of University of Toronto; 2019.

61. Da Silva SL, Jeelani Y, Dang H, Krieger MD, McComb JG. Risk factors for hydrocephalus and neurological deficit in children born with an encephalocele. Journal of neurosurgery Pediatrics. 2015;15(4):392-8.

62. Biard JM, Johnson MP, Carr MC, Wilson RD, Hedrick HL, Pavlock C, et al. Long-term outcomes in children treated by prenatal vesicoamniotic shunting for lower urinary tract obstruction. Obstetrics and gynecology. 2005;106(3):503-8.

63. Berte N, Vrillon I, Larmure O, Gomola V, Ayav C, Mazeaud C, et al. Long-term renal outcome in infants with congenital lower urinary tract obstruction. Progres en urologie : journal de l'Association francaise d'urologie et de la Societe francaise d'urologie. 2018;28(12):596-602.

64. Wu Y, Al-Janabi H, Mallett A, Quinlan C, Scheffer IE, Howell KB, et al. Parental health spillover effects of paediatric rare genetic conditions. Quality of life research : an international journal of quality of life aspects of treatment, care and rehabilitation. 2020;29(9):2445-54.

65. Heron J, O'Connor TG, Evans J, Golding J, Glover V. The course of anxiety and depression through pregnancy and the postpartum in a community sample. Journal of affective disorders. 2004;80(1):65-73.

66. Alkan F, Sertcelik T, Yalın Sapmaz S, Eser E, Coskun S. Responses of mothers of children with CHD: quality of life, anxiety and depression, parental attitudes, family functionality. Cardiology in the young. 2017;27(9):1748-54.

67. Woolf-King SE, Anger A, Arnold EA, Weiss SJ, Teitel D. Mental Health Among Parents of Children With Critical Congenital Heart Defects: A Systematic Review. Journal of the American Heart Association. 2017;6(2).

68. Swanepoel M, Haw T. A pilot study evaluating depression in mothers with children diagnosed with Down syndrome in state health care. Journal of intellectual disability research : JIDR. 2018;62(11):952-61.

69. Hinton L, Locock L, Long AM, Knight M. What can make things better for parents when babies need abdominal surgery in their first year of life? A qualitative interview study in the UK. BMJ open. 2018;8(6):e020921.

70. Arnold HE, Baxter KJ, Short HL, Travers C, Bhatia A, Durham MM, et al. Short-term and family-reported long-term outcomes of simple versus complicated gastroschisis. The Journal of surgical research. 2018;224:79-88.

71. Carpenter JL, Wiebe TL, Cass DL, Olutoye OO, Lee TC. Assessing quality of life in pediatric gastroschisis patients using the Pediatric Quality of Life Inventory survey: An institutional study. Journal of pediatric surgery. 2016;51(5):726-9.

72. Solberg Ø, Dale MT, Holmstrøm H, Eskedal LT, Landolt MA, Vollrath ME. Emotional reactivity in infants with congenital heart defects and maternal symptoms of postnatal depression. Archives of women's mental health. 2011;14(6):487-92.

73. Solberg Ø, Dale MT, Holmstrøm H, Eskedal LT, Landolt MA, Vollrath ME. Long-term symptoms of depression and anxiety in mothers of infants with congenital heart defects. Journal of pediatric psychology. 2011;36(2):179-87.

74. Mahapatra AK. Giant encephalocele: a study of 14 patients. Pediatric neurosurgery. 2011;47(6):406-11.

75. Date I, Yagyu Y, Asari S, Ohmoto T. Long-term outcome in surgically treated encephalocele. Surgical neurology. 1993;40(2):125-30.

76. Lo BW, Kulkarni AV, Rutka JT, Jea A, Drake JM, Lamberti-Pasculli M, et al. Clinical predictors of developmental outcome in patients with cephaloceles. Journal of neurosurgery Pediatrics. 2008;2(4):254-7.

77. Winterberg PD, Garro R. Long-Term Outcomes of Kidney Transplantation in Children. Pediatric clinics of North America. 2019;66(1):269-80.

78. Kaimal AJ, Norton ME, Kuppermann M. Prenatal Testing in the Genomic Age: Clinical Outcomes, Quality of Life, and Costs. Obstetrics and gynecology. 2015;126(4):737-46.

79. Le QA, Doctor JN, Zoellner LA, Feeny NC. Minimal clinically important differences for the EQ-5D and QWB-SA in Post-traumatic Stress Disorder (PTSD): results from a Doubly Randomized Preference Trial (DRPT). Health and quality of life outcomes. 2013;11:59.

80. NHS England. National Schedule of NHS Costs 2019/20 [Internet]. NHS England. 2021 [cited 27 October 2021]. Available from: <https://www.england.nhs.uk/national-cost-collection/#nccdata2>.

81. Campbell HE, Kurinczuk JJ, Heazell A, Leal J, Rivero-Arias O. Healthcare and wider societal implications of stillbirth: a population-based cost-of-illness study. BJOG : an international journal of obstetrics and gynaecology. 2018;125(2):108-17.

82. Jones K, Burns, A. Unit Costs of Health and Social Care 2021. Canterbury: University of Kent; 2021.

83. McCrone P. DS, Patel A., Knapp M., Lawton-Smith S. . Paying the Price - The cost of mental health care in England to 2026. London: The Kings Fund; 2008.

84. Meert KL, Shear K, Newth CJ, Harrison R, Berger J, Zimmerman J, et al. Follow-up study of complicated grief among parents eighteen months after a child's death in the pediatric intensive care unit. Journal of palliative medicine. 2011;14(2):207-14.

85. Senses Dinc G, Cop E, Tos T, Sari E, Senel S. Mothers of 0-3-year-old children with Down syndrome: Effects on quality of life. Pediatrics international : official journal of the Japan Pediatric Society. 2019;61(9):865-71.

86. NHS Employers. National job profiles: NHS Employers; 2022 [Available from: <https://www.nhsemployers.org/articles/national-job-profiles>.

87. Professional Standards Authority. Right-touch assurance for sonographers based on risk of harm arising from practice. Report to Health Education England. London; 2019.

88. Office for National Statistics. Births in England and Wales: 2020 Live births, stillbirths and the intensity of childbearing, measured by the total fertility rate [Internet]. 2020 [cited 27th March 2022]. Available from: <https://www.ons.gov.uk/peoplepopulationandcommunity/birthsdeathsandmarriages/livebirths/bulletins/birthsummarytablesenglandandwales/2020>.

89. Karim J, Pandya P, McHugh A, Papageorghiou AT. OC23.06: Significant variation in practice for first trimester anatomy assessment: results from a nationwide survey. Ultrasound in Obstetrics & Gynecology. 2019;54:60-1.

90. Fenwick E, Steuten L, Knies S, Ghabri S, Basu A, Murray JF, et al. Value of Information Analysis for Research Decisions-An Introduction: Report 1 of the ISPOR Value of Information Analysis Emerging Good Practices Task Force. Value in health : the journal of the International Society for Pharmacoeconomics and Outcomes Research. 2020;23(2):139-50.

91. Wilson ECF. A Practical Guide to Value of Information Analysis. PharmacoEconomics. 2015;33(2):105-21.

92. Paulden M. Calculating and Interpreting ICERs and Net Benefit. PharmacoEconomics. 2020;38(8):785-807.

93. Strong M, Oakley JE, Brennan A. Estimating multiparameter partial expected value of perfect information from a probabilistic sensitivity analysis sample: a nonparametric regression approach. Med Decis Making. 2014;34(3):311-26.

94. Pogany L, Barr RD, Shaw A, Speechley KN, Barrera M, Maunsell E. Health status in survivors of cancer in childhood and adolescence. Quality of life research : an international journal of quality of life aspects of treatment, care and rehabilitation. 2006;15(1):143-57.

95. Petrou S, Kupek E. Estimating preference-based health utilities index mark 3 utility scores for childhood conditions in England and Scotland. Med Decis Making. 2009;29(3):291-303.
